# Microbial diversity on Icelandic glaciers and ice caps

**DOI:** 10.3389/fmicb.2015.00307

**Published:** 2015-04-20

**Authors:** Stefanie Lutz, Alexandre M. Anesio, Arwyn Edwards, Liane G. Benning

**Affiliations:** ^1^Cohen Laboratories, School of Earth and Environment, University of LeedsLeeds, UK; ^2^Bristol Glaciology Centre, School of Geographical Sciences, University of BristolBristol, UK; ^3^Institute of Biological, Environmental and Rural Sciences, Aberystwyth UniversityAberystwyth, UK; ^4^Interdisciplinary Centre for Environmental Microbiology, Aberystwyth UniversityAberystwyth, UK; ^5^GFZ German Research Centre for Geosciences, Helmholtz Centre PotsdamPotsdam, Germany

**Keywords:** snow algae, Iceland, glaciers, microbial diversity, bacteria, archaea, sequencing, albedo

## Abstract

Algae are important primary colonizers of snow and glacial ice, but hitherto little is known about their ecology on Iceland's glaciers and ice caps. Due do the close proximity of active volcanoes delivering large amounts of ash and dust, they are special ecosystems. This study provides the first investigation of the presence and diversity of microbial communities on all major Icelandic glaciers and ice caps over a 3 year period. Using high-throughput sequencing of the small subunit ribosomal RNA genes (16S and 18S), we assessed the snow community structure and complemented these analyses with a comprehensive suite of physical-, geo-, and biochemical characterizations of the aqueous and solid components contained in snow and ice samples. Our data reveal that a limited number of snow algal taxa (*Chloromonas polyptera*, *Raphidonema sempervirens* and two uncultured *Chlamydomonadaceae)* support a rich community comprising of other micro-eukaryotes, bacteria and archaea. *Proteobacteria* and *Bacteroidetes* were the dominant bacterial phyla. Archaea were also detected in sites where snow algae dominated and they mainly belong to the *Nitrososphaerales*, which are known as important ammonia oxidizers. Multivariate analyses indicated no relationships between nutrient data and microbial community structure. However, the aqueous geochemical simulations suggest that the microbial communities were not nutrient limited because of the equilibrium of snow with the nutrient-rich and fast dissolving volcanic ash. Increasing algal secondary carotenoid contents in the last stages of the melt seasons have previously been associated with a decrease in surface albedo, which in turn could potentially have an impact on the melt rates of Icelandic glaciers.

## Introduction

Glaciers and ice sheets cover about 10% of the Earth's surface and are the largest freshwater reservoir. They are a critical component of the Earth's climate system and with temperatures rising globally, melting rates are increasing affecting freshwater availability and sea level rise (Meier et al., 2007). Glacial surfaces have not been considered to harbor much life until recently (Hodson et al., 2008; Anesio and Laybourn-Parry, 2012). They are considered an extreme environment yet they contain species of all three domains of life including bacteria, archaea, fungi, protozoa, and even invertebrates (Anesio and Laybourn-Parry, 2012). Among glacial surface habitats, cryoconite holes (cyanobacteria dominated water-filled holes formed by the preferential melt of organic and inorganic dark particles) have been by far the more extensively studied habitats (Cameron et al., 2012; Edwards et al., 2014). However, the largest proportion (>90%) of glacial surfaces is covered by snow and increasingly by bare ice toward the end of the melting season. Snow algae (*Chlorophyta*) are the most prolific and colorfully striking microbial species colonizing snow and ice surfaces. First described by the ancient Greek Aristotle (Gentz-Werner, 2007), snow algae have been known for a long time and they have been studied in many polar and alpine cryospheric settings including Greenland (Lutz et al., 2014), Svalbard (Müller et al., 2001; Leya et al., 2004), the European Alps (Remias et al., 2005), the Rocky Mountains (Thomas and Duval, 1995), Antarctica (Fujii et al., 2010; Remias et al., 2013), Alaska (Takeuchi, 2013) and the Himalayans (Yoshimura et al., 2006). We have recently shown that they are important primary colonizers and net primary producers supporting other snow and ice microbial communities as carbon and nutrient sources (Lutz et al., 2014). As part of their life cycle and as a mechanism of protection from high irradiation, snow algal species produce red pigments (carotenoids). Through this protective reaction, algal blooms color snow and ice surfaces and cause a darkening of glacial surfaces which in turn leads to a decrease in surface albedo (Thomas and Duval, 1995; Yallop et al., 2012; Takeuchi, 2013; Benning et al., 2014; Lutz et al., 2014). Such a decrease of albedo may speed up melting processes even further. This is of special interest in Iceland where glaciers have been shown to be retreating very fast (Staines et al., 2014) and where albedo is also affected by the presence of volcanic dust and ash on snow and ice surfaces.

Currently, not a single description of snow algae from any of the glaciers or ice caps in Iceland is available in the literature and no Icelandic snow algal species are available in cryogenic culture collections. Thus, we do not know if they are present, and if so, if they are abundant or what their bio-geographical distribution or ecological role might be. This is despite the fact that anecdotal evidence from scientists working on Iceland's glaciers and ice caps (e.g., personal communication from Glaciology Prof. Magnús Tumi Guðmundsson, University of Iceland) suggests that occasionally in the late summer “reddish snow” patches can be observed. Icelandic glaciers represent a special case of glacial ecosystems due to their vicinity to active volcanoes and thus constant input of fresh ash through dust or eruptions. The very abundant dark ash that covers most snow and ice fields on Iceland's glaciers and ice caps in the summer melting season is most likely also the reason why so far colored snow algae have not been described. The darkness of volcanic ash contributes to the darkening of Icelandic glacial surfaces and their faster melting (Guðmundsson et al., 2005; Möller et al., 2014). Possibly, this effect also extends the active growth season of snow algae due to earlier and prolonged availability of liquid water. The highly soluble volcanic ash (Ritter, 2007; Jones and Gislason, 2008) is an important source of essential nutrients (e.g., N, P, Fe) and thus could be a good substrate for snow algal growth, which may further enhance the negative effect on surface albedo.

With this study we aimed to identify the presence of snow and ice algae on Icelandic glacial surfaces. Furthermore, we wanted to detail their associated microbial communities, and finally place the communities on all major Icelandic glaciers and ice caps in the context of variations in biogeography and physico–chemical parameters of snow and ice.

## Materials and methods

### Field site, sampling, and measurements

**Figure 1:**
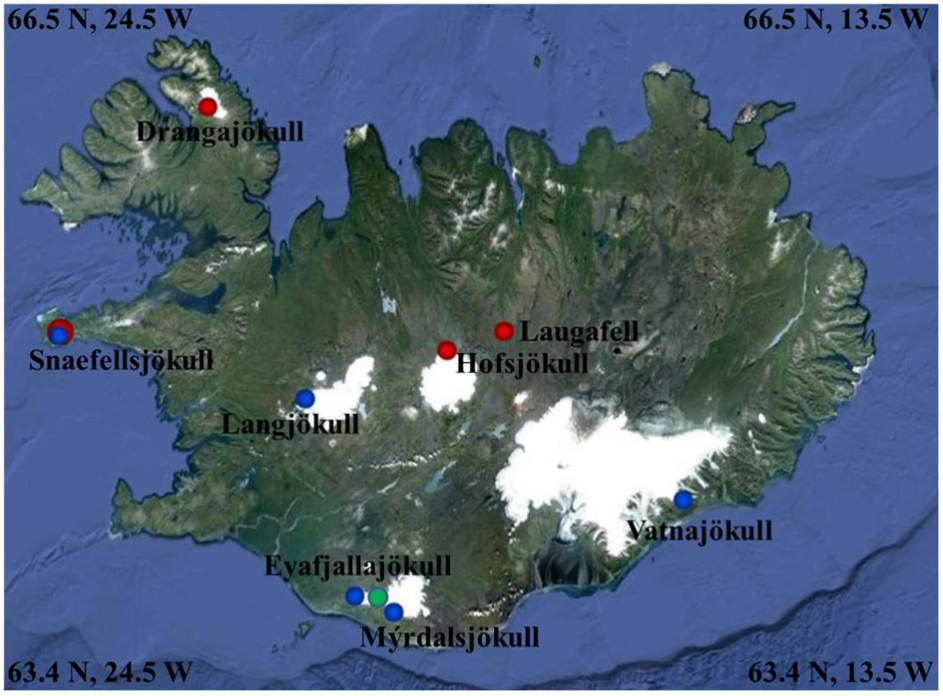
**Map showing the 2012 (red dots), 2013 (blue dots), and 2014 (green dot) sampling sites (further details see Table 1)**. Image Source Google Earth (June 2013).

A total of 33 snow and 1 ice samples (labeled with “ICE” for Iceland, followed by the collection year and sample number: ICE-12_1–7, ICE-13_1–24 and ICE-14_1–3; Table 1) was collected from seven glaciers and one ice cap in Iceland (see Figure 1 and Table 1 for details). Snow fields in the terminus areas of the western glacier Snaefellsjökull, the northern glacier Drangajökull, the central glacier Hofsjökull, as well as a large permanent snow field near Laugafell in the Central Highlands were sampled at the end of July in 2012. In early June 2013 we sampled the terminus areas of the southern glaciers Vatnajökull, Eyafjallajökull, Mýrdalsjökull, and Solheimajökull and the western glaciers Snaefellsjökull and Langjökull. Finally, we sampled three snow fields that covered fresh lava fields from the 2010 eruption of Eyafjallajökull at the end of August in 2014 in order to also assess how and if fresh microbial colonization had occurred. It is worth noting that in 2012 and 2014 melting had been very advanced leading to thin snow covers on the termini of all glaciers and smaller permanent snow fields. However, microbial colonization was well-developed at all sites. In contrast, the samples in 2013 were collected in early June, when melting had just been initiated and thick snow packs were still present at all sites and microbial colonization was less prominent or widely distributed. Nevertheless, at each site, regardless of years and stage of the melting season, we collected where possible two adjacent samples: one clean snow sample (no macroscopically visible particles) and one red snow sample (with visible particles). The exceptions were Solheimajökull, sampled in 2013, where snow patches were only present at the edges of deep crevasses and thus only bare, gray ice was sampled and Eyafjallajökull sampled from 2014, where only red snow and no “clean” snow could be found at the late stage in the melt season. It is important to note that all samples that are labeled “red snow” or “gray ice” in Table 1 always contained high loads of volcanic ash or dust debris, while the samples termed “clean snow” did not contain ash, dust or any macroscopically visible biomass and filtering of the clean snow did not result in enough biomass for genomic or other analyses of the particulates.

**Table 1:** **Overview of samples, locations, coordinates and field measurements**.

**Sample label**	**Glacier**	**Sample description**	**Collection date**	**GPS location [UTM]**	**Elevation [m]**	**pH**	**Snow temp. [°C]**	**PAR [W/m^2^]**	**UV-A [W/m^2^]**	**UV-B [W/m^2^]**	**Albedo [%]**
ICE-12_1	Snaefellsjökull	Red snow	26/07/2012	27W 0371148 E, 7190818 N	695						
ICE-12_2	Drangajökull	Red snow	27/07/2012	27W 0442009 E, 7334293 N	230	5.27	0.3				
ICE-12_3	Drangajökull	Red snow	27/07/2012	27W 0442125 E, 7334250 N	196	6.15	0				
ICE-12_4	Laugafell snow field	Red snow	29/07/2013	27W 0632892 E, 7222179 N	908	4.96		237	21.2	1.24	42
ICE-12_5	Laugafell snow field	Clean snow	29/07/2013	27W 0632892 E, 7222179 N	908	5.68	0.1	254			39
ICE-12_6	Hofsjökull	Red snow	29/07/2013	27W 0600998 E, 7206978 N	906	6.44	0.5	104			
ICE-12_7	Hofsjökull	Red snow	29/07/2013	27W 0600998 E, 7206978 N	906	6.47	0	98			19
ICE-13_1	Snaefellsjökull	Red snow	02/06/2013	27W 0372788 E, 7188065 N	461	5.46	0	100	20.8	0.61	64
ICE-13_2	Snaefellsjökull	Red snow	02/06/2013	27W 0372427E, 7188287 N	366	5.07	0.2				
ICE-13_3	Eyjafjallajökull	Clean snow	07/06/2013	27V 0563957 E, 7056674 N	1156	5.59	0.1	356			78
ICE-13_4	Eyjafjallajökull	Red snow	07/06/2013	27V 0563563 E, 7056695 N	1121	5.10	0.2	316			73
ICE-13_5	Eyjafjallajökull	Red snow	07/06/2013	27V 0559557 E, 7056046 N	736	5.50	0	94			33
ICE-13_6	Eyjafjallajökull	Red snow	07/06/2013	27V 0559557 E, 7056046 N	736	5.55	0	89			65
ICE-13_7	Mýrdalsjökull	Clean snow	07/06/2013	27V 0587893 E, 7049973 N	925	5.20	1	245			89
ICE-13_8	Mýrdalsjökull	Red snow	07/06/2013	27V 0588165 E, 7049601 N	923	5.86	1	150	18.6	0.42	72
ICE-13_9	Mýrdalsjökull	Red snow	07/06/2013	27V 0588165 E, 7049601 N	923	5.96	0	118			55
ICE-13_10	Solheimajökull	Gray ice	07/06/2013	27V 0582585 E, 7046897 N	244			127			42
ICE-13_11	Vatnajökull	Clean snow	08/06/2013	28W 0459058 E, 7125767 N	767	5.98	0	173			80
ICE-13_12	Vatnajökull	Gray ice	08/06/2013	28W 0459058 E, 7125451 N	695			136	19	0.06	18
ICE-13_13	Vatnajökull	Red snow	08/06/2013	28W 0459058 E, 7125451 N	695	7.08	0	137			27
ICE-13_14	Vatnajökull	Red snow	08/06/2013	28W 0459058 E, 7125451 N	695	6.04	0	148			42
ICE-13_15	Vatnajökull	Red snow	08/06/2013	28W 0459058 E, 7125451 N	695	5.83	0	151			47
ICE-13_16	Langjökull	Red snow	10/06/2013	27W 0521378 E, 7168876 N	812	6.53	0	52	6.42	0.21	60
ICE-13_17	Langjökull	Clean snow	10/06/2013	27W 0521378 E, 7168876 N	812	6.26	0	54			67
ICE-13_18	Langjökull	Red snow	10/06/2013	27W 0521378 E, 7168876 N	812	5.93	0	88			43
ICE-13_19	Langjökull	Red snow	10/06/2013	27W 0521378 E, 7168876 N	812	5.89	0	96			47
ICE-13_21	Snaefellsjökull	Red snow	12/06/2013	27W 0370323 E, 7188655 N	705	5.31	0	117			57
ICE-13_22	Snaefellsjökull	Clean snow	12/06/2013	27W 0370323 E, 7188655 N	705	5.84	0	122			71
ICE-13_23	Snaefellsjökull	Red snow	12/06/2013	27W 0370323 E, 7188655 N	705	6.19	0	170			73
ICE-13_24	Snaefellsjökull	Red snow	12/06/2013	27W 0370323 E, 7188655 N	705	5.96	0	195			51
ICE-14_1	Eyjafjallajökull	Red snow	28/08/2014	27V 0577039 E, 7057638 N	1004	7.73	0				
ICE-14_1	Eyjafjallajökull	Red snow	28/08/2014	27V 0577174 E, 7057468 N	1024	7.78	0				
ICE_14_3	Eyjafjallajökull	Red snow	28/08/2014	27V 0577101 E, 7057803 N	1010	7.91	0				

*Conductivity in all samples was 0 μS/cm*.

At each sampling point prior to sample collection, snow temperature, pH and conductivity were measured in the field using a daily calibrated multi-meter (Hanna instruments, HI 98129). Irradiation was measured using a radiometer with specific PAR, UV-A and UV-B sensors (SolarLight, PMA2100). Surface albedo was calculated by taking the ratio of reflected to incident radiation (400–700 nm range) as previously described (Lutz et al., 2014). Snow samples were collected either in sterile 50 mL centrifuge tubes (red snow) or large sterile *Whirl-Pak*® bags (clean snow) and in 250 mL pre-ashed (450°C >4 h) glass jars for all organic analyses. The snow samples were slowly melted at room temperature over a ~ 6 h period. All samples were processed (filtered, acidified, etc.) within max 6–8 h post collection in order to preserve them for various analyses in the home laboratory. All DNA and filtered organic samples were flash-frozen in liquid nitrogen and returned to Leeds in a cryo-shipper after which they were stored at −80°C until further processing. All processed inorganic samples were stored cold (4°C, in the dark) until analyzed.

### Bio- and geochemical analyses

Several of the methods used to analyze solutions and solid samples described below are equivalent to the methods employed and explained in detail in Lutz et al. (2014). Here we briefly summarize all standard aqueous and solid analyses and explain in detail those methods that are new compared to our previous work. For anion analysis by ion chromatography (IC, Dionex, 5% precision) and cation analysis by inductively coupled plasma mass spectrometry (ICP-MS, Agilent, 3% precision), samples were filtered through 0.2 μM cellulose-acetate filters into either pre-acidified (Aristar grade HNO_3_) Nalgene HDPE bottles (cations) or into un-acidified 15 mL centrifuge tubes (anions). For dissolved organic carbon (DOC), PO_4_ and organic particulate analysis, samples collected in ashed glass jars were filtered through ashed 0.7 μm glass fiber filters (GFF) directly into pre-acidified (Aristar grade HCl) vials using glass syringes and metal filter holders. PO_4_ was analyzed by segmented flow-injection analyses (AutoAnalyser3, Seal Analytical, 5% precision), while DOC was analyzed on a total organic carbon analyzer (TOC, Shimadzu TOC-V, 3% precision). The GFF filters containing particulates were preserved in pre-ashed aluminum foil for pigment and fatty acid analyses. Pigment compositions (chlorophylls and carotenoids) were analyzed using high-pressure liquid chromatography (HPLC, Agilent 1220 Infinity, 5% precision) after extraction in dimethylformamide and quantified using pigment standards (chlorophylls: Sigma, carotenoids: Carotenature). Fatty acids were extracted in dichloromethane:methanol (2:1, v:v) in two steps and extracts were combined, followed by transesterification in 3 M methanolic HCl for 20 min at 65°C and three consecutive extractions in isohexane. Tricosanoic acid methyl ester (Sigma) was used as an internal standard and a 37 component FAME mix (Supelco) as external standard. The extracts were separated by gas chromatography (Thermo Scientific, Trace1300, 5% precision), spectra were recorded on a mass spectrometer (ISQ Single Quadrupole) and quantified on a flame ionization detector (FID). Particulates were also collected on 0.2 μm polycarbonate filters for mineralogical analysis by X-ray diffraction (XRD, D8 Bruker). Total carbon (TC), nitrogen (TN) and sulfur (TS) and nitrogen isotopes were analyzed by pyrolysis at 1500°C (Vario Pyro Cube, Elementar Inc.) followed by mass spectrometry (Isoprime Mass Spectrometer, 0.1% precision). For imaging by light microscopy (LM, Leica DM750) unconcentrated samples were preserved in 2.5% glutaraldehyde and images recorded through a 63× objective. The hydrogeochemical modeling software *PHREEQC* (Parkhurst, 1995, using the LLNL database) was used to determine the saturation indexes for our aqueous solutions.

### DNA sequencing

All samples contained low amounts of biomass and therefore, red snow samples from the same glacier and same collection year were pooled in order to obtain a sufficient quantity of DNA for sequencing (**Table 6**). Total DNA was extracted using the PowerSoil® DNA Isolation kit (MoBio Laboratories). 16S rRNA genes were amplified using bacterial primers 27F (5′-AGAGTTTGATCMTGGCTCAG) and 357R (5′-CTGCTGCCTYCCGTA) (tagged with the Ion Torrent adapter sequences and MID barcode) spanning the V1–V2 hypervariable regions. 18S rRNA genes were amplified using the eukaryotic primers 528F (5′-GCGGTAATTCCAGCTCCAA) and 706R (5′-AATCCRAGAATTTCACCTCT) (Cheung et al., 2010) (tagged with the Ion Torrent adapter sequences and MID barcode) spanning the V4–V5 hypervariable region. Polymerase chain reactions (PCR) were performed using Platinum® PCR SuperMix High Fidelity according to manufacturer's protocols. Initial denaturation at 95°C for 5 min was followed by 30 cycles of denaturation at 95°C for 30 s, annealing at 60°C for 30 s and elongation at 72°C for 30 s. Final elongation was at 72°C for 7 min. Archaeal 16S rRNA genes were amplified following a nested PCR approach. The first PCR reaction was carried out using primers 20F and 915R. Initial denaturation at 95°C for 5 min was followed by 35 cycles of denaturation at 95°C for 30 s, annealing at 62°C for 30 s and elongation at 72°C for 180 s. Final elongation was at 72°C for 10 min. The PCR product was used as template for the second PCR reaction with primers 21F (5′-TCCGGTTGATCCYGCCGG) and 519R (5′- GWATTACCGCGGCKGCTG) (tagged with the Ion Torrent adapter sequences and MID barcode) spanning the V1–V2 hypervariable region. Initial denaturation at 95°C for 5 min was followed by 30 cycles of denaturation at 95°C for 30 s, annealing at 60°C for 30 s and elongation at 72°C for 30 s. Final elongation was at 72°C for 7 min. All PCRs were carried out in triplicates to reduce amplification bias and in reaction volumes of 1 × 25 and 2 × 12.5 μl. All pre-amplification steps were done in a laminar flow hood with DNA-free certified plastic ware and filter tips. The pooled amplicons were purified with AMPure XP beads (Agencourt^©^) with a bead to DNA ratio of 0.6 to remove nucleotides, salts and primers and analyzed on the Agilent 2100 Bioanalyser (Agilent Technologies) with the High Sensitivity DNA kit (Agilent Technologies) and quality, size and concentration were determined. Sequencing was performed on an Ion Torrent Personal Genome Machine using the Ion Xpress™ Template Kit and the Ion 314^TM^ chip following manufacturer's protocols. The only exceptions were the archaeal amplicons of samples ICE-14_1, ICE-14_2 and ICE-14_3 which were sequenced on an Ion 316^TM^ chip. The raw sequence data was processed in QIIME (Caporaso et al., 2010). Barcodes and adapter sequences were removed from each sequence. Filtering of sequences was performed using an average cutoff of Q20 over a 350 bp range. Reads shorter than 200 bp were removed. OTUs were picked *de novo* using a threshold of 99, 97, and 95% identity. Taxonomic identities were assigned for representative sequences of each OTU using the reference databases Greengenes for bacteria and archaea. The Silva database (DeSantis et al., 2006; extended with additional 223 sequences of cryophilic algae kindly provided by Dr. Thomas Leya from the CCCryo—Culture Collection of Cryophilic Algae, Fraunhofer IZI-BB) was used for eukaryotes. Data were aligned using PyNAST and a 0.80 confidence threshold. Singletons were excluded from the analysis. For bacterial sequence matching, plant plastids were removed from the data set prior to further analysis. For eukaryotic sequence matching *Chloroplastida* were pulled out of the data set and stored in a separate OTU table. In order to focus upon algal diversity, sequences matching *Embryophyta* (e.g., moss, fern) were removed from the data set. For archaea, sequences matching bacteria were removed. Finally, for diversity analyses samples were rarefied to the smallest sequence number and Shannon indices were calculated in QIIME. A matrix of each OTU table representing relative abundance was imported into Past3 (Hammer et al., 2012) for multivariate statistical analyses (principal component analysis, PCA). Representative sequences of the major algal species found in all samples were imported into Geneious (7.1.3., Biomatters) for phylogenetic tree building based on neighbor-joining. Sequences have been deposited to the European Nucleotide Archive (ENA) under accession number PRJEB8832.

## Results

### Physico-, geochemical, and biochemical analyses

Snow temperatures at each collection site varied only over a narrow range between 0 and 1.0°C (Table 1). The pH was slightly acidic for most sites and there were no clear differences between red algal (4.96–6.53) or clean snow (5.20–6.26) sites. Only the samples collected in 2014 from snow fields on fresh volcanic lava and that had high contents of fresh volcanic ash inputs from the 2010 Eyafjallajökull eruption showed a more alkaline pH (7.73–7.91). This is not surprising since fresh volcanic glass is highly reactive and buffers any waters in contact with it to a pH between 7.5 and 8 (Oelkers and Gislason, 2001; Gislason and Oelkers, 2003). The albedo values differed from clean snow (76% ± 8) to sites with algal growth (56% ± 14; Table 1). Aqueous geochemical analysis (Table 2) revealed low values (<ppm) for all cations and anions in our samples. Geochemical modeling confirmed that our solutions were undersaturated with respect to most solid phases except for Fe oxides (goethite, hematite) and As-hydroxides (boehmite, diaspore, gibbsite, Table S7). However, values for DOC varied dramatically and ranged from 15 to 200 μM (Table 2). The total carbon contents (TC, in % of total dry filtered particulate weight) were below 2% in all sites with one exception (ICE-13_1, Snaefellsjökull) where a TC content of 7.6% was found (Table 3). In all analyzed samples the total nitrogen contents were below 0.3% and total sulfur below 0.1%. There were no large variations in N and S among the sample sites. Carbon to nitrogen (C/N) ratios varied over a broad range from 1.7 (C/N) ratios varied over a broad range from 1.7 (ICE-13_8, Mýrdalsjökull) to 28.5 (ICE-13_1, Snaefellsjökull). Nitrogen isotopes were overall negative and ranged from −11.2 to −4.2‰.

**Table 2:** **Combined organic (DOC) and inorganic aqueous chemical data for the samples collected in 2012 and 2013**.

**Sample**	**DOC [μM C]**	**PO^3−^_4_ [nM P]**	**NO^−^_3_**	**SO^2−^_4_**	**Cl^−^**	**Al**	**Ba**	**Ca**	**Cr**	**Cu**	**Fe**	**K**	**Mg**	**Mn**	**Na**	**Ni**	**Pb**	**S**	**Si**	**Sr**	**Zn**
ICE-12_1			<	171	243	3	1	15	<	<	5	14	10	1	124	<	<		76	<	2
ICE-12_2			<	<	135	560	7	200	2	1	337	55	131	5	214	1	<		626	2	22
ICE-12_3			<	<	114	17	7	22	1	1	17	38	11	<	99	<	<		40	<	25
ICE-12_4			<	181	275	4	13	26	<	<	3	44	13	1	234	<	<		483	<	30
ICE-12_5			99	<	125	6	13	41	<	<	6	34	4	<	57	<	<		24	<	31
ICE-12_6			<	<	100	12	11	21	7	1	40	33	15	2	98	3	<		264	<	40
ICE-12_7			<	<	275	101	22	166	<	1	84	73	113	5	231	<	<		734	1	30
ICE-13_1	94	117.9	371	127	255	1.4	<	45.3	<	<	4.6	<	15.1	1.4	209	<	<	<	63.3	0.49	1.6
ICE-13_2	200	89.2	<	<	176	1.4	<	37.3	<	<	5.1	<	10.4	1.3	132	<	<	<	72.5	0.38	2
ICE-13_3		92.7	<	<	147	0.59	<	4.8	<	<	8.9	<	2.8	0.24	89.3	<	<	<	10.1	<	0.28
ICE-13_4	82	119.8	<	<	363	1.7	<	18.4	<	<	1.3	29.8	11.8	0.84	245	<	<	<	36.9	0.26	0.19
ICE-13_5	33	84.9	<	127	377	6.4	<	86.6	<	<	12.9	32.3	46.4	4.9	266	<	<	<	193	0.68	0.22
ICE-13_6	86	78.6	<	<	275	1.7	<	19.2	<	<	3.2	28.5	13.5	1	145	<	<	<	54.3	0.27	0.24
ICE-13_7		43.5	<	128	539	0.68	<	1.1	<	<	0.3	<	0.8	0.05	3.3	<	<	<	10.8	<	2.3
ICE-13_8	58	172.0	<	<	488	4.7	<	94.5	<	<	9.1	28.7	34.2	0.95	398	<	<	<	94.5	0.43	0.22
ICE-13_9	15	296.3	<	<	156	14.7	<	132	<	<	29.7	30.8	54.5	2.2	156	<	<	<	330	0.57	<
ICE-13_10			<	145	95	1.9	<	20.1	<	<	2.1	15.4	7.5	0.72	126	<	<	20.5	21.1	0.14	1.6
ICE-13_11	201	74.7	100	<	0	0.51	<	5.9	<	<	0.4	<	2.2	0.06	41.2	<	<	<	20.2	<	1.1
ICE-13_12	92	230.4	<	<	0	11.4	0.14	89.2	6.2	0.18	14.5	<	30.8	3.1	76.1	<	0.02	<	63.4	0.57	1.8
ICE-13_13	50	89.3	<	<	274	0.98	<	31	<	<	1.1	<	9.9	0.44	198	<	<	<	20.3	0.17	0.7
ICE-13_14	37	308.5	<	<	371	4.6	<	84.2	<	<	8.8	43.1	53.9	2.6	188	<	<	<	306	0.45	0.22
ICE-13_15	33	262.7	<	<	100	5.8	<	48	<	<	11.9	<	28.9	1.4	98.7	<	<	<	185	0.23	<
ICE-13_16	54	53.9	3499	<	0	1.2	<	12.8	<	<	3.1	<	5.4	0.57	47	<	<	<	35.8	0.12	0.96
ICE-13_17	26	448.9	225	<	72	1.4	<	<	<	<	1.1	16.4	0.8	0.05	67.8	<	<	<	10.5	<	0.89
ICE-13_18	149	78.3	<	<	79	8.3	<	20.6	<	0.39	2.1	17.6	6.5	0.36	70.5	<	<	<	69.8	0.21	0.76
ICE-13_19	41	62.8	<	<	486	8.1	<	24.5	<	0.32	2.1	<	7.8	0.33	385	<	0.56	<	64.7	0.23	1
ICE-13_21	58	54.5	<	<	718	2.1	<	26	<	<	9.1	18.6	14.4	0.95	523	<	0.03	<	18.8	0.26	3.6
ICE-13_22	103	85.8	<	<	881	1.1	<	12.5	<	<	1.3	<	18.7	0.08	658	<	0.02	18.8	10.1	0.18	0.61
ICE-13_23	59	30.2	456	<	742	0.52	<	3.9	0.27	<	2.3	12.5	2.5	0.1	577	<	<	<	23.2	<	0.29
ICE-13_24	58	145.8	468	145	611	1.8	<	7.9	<	<	7.7	<	11.4	1	386	<	<	16.4	38.3	0.19	0.12

*All values except DOC and PO^3−^_4_ are given in ppb; NO^3−^, SO^2−^_4_, and Cl^−^ all determined by IC, all others analyzed by ICP-MS; limit of detection (LOD) for IC: NO^−^_3_ = 96 ppb, Cl^−^ = 72 ppb, SO^2−^_4_ = 121 ppb, LOD's for ICP-MS: Al, Ba, Co, Cr, Cu, Fe, Mg, Ni, Si, Sr, Zn = 0.1 ppb, Bi, Cd, Mn, Pb = 0.01 ppb, Ca, Na = 1 ppb, K,P,S = 10 ppb, no value indicates no measurement available for the respective sample; note Co was < LOD in all samples and Co was < LOD in all samples except in ICE-13_19 (0.07)*.

**Table 3:** **Total carbon (TC_(s)_), total nitrogen (TN_(s)_), and total sulfur (TS_(s)_) (all based on % of dry weight of sample) as well as the nitrogen isotope values from the analyzed particulates in the 2012 and 2013 collected red snow and gray ice samples that contained enough particulate material for analyses; listed are also the solid C/N_(s)_ ratio calculated from TC and TN values**.

**Sample ID**	**Glacier**	**TC_(s)_ [%]**	**TN_(s)_ [%]**	**TS_(s)_ [%]**	**C/N_(s)_**	**d^15^N_(s)_ [‰]**
ICE-12_7	Hofsjökull	0.64	0.12	0.05	5.4	−11.2
ICE-13_1	Snaefellsjökull	7.62	0.27	0.09	28.5	
ICE-13_2	Snaefellsjökull	1.69	0.08	0.08	20.7	
ICE-13_4	Eyafjallajökull	0.08	0.01	0.02	6.4	
ICE-13_5	Eyafjallajökull	1.27	0.07	0.09	18.4	−4.2
ICE-13_6	Eyafjallajökull	0.64	0.03	0.07	19.0	−7.0
ICE-13_8	Mýrdalsjökull	0.02	0.01	0.04	1.7	
ICE-13_9	Mýrdalsjökull	0.06	0.01	0.06	4.3	
ICE-13_10	Solheimajökull	0.25	0.01	0.08	19.4	
ICE-13_12	Vatnajökull	0.19	0.03	0.09	7.4	−6.2
ICE-13_14	Vatnajökull	0.09	0.02	0.05	3.6	−7.5
ICE-13_15	Vatnajökull	0.07	0.01	0.05	6.3	
ICE-13_16	Langjökull	0.22	0.02	0.05	10.0	−5.7
ICE-13_18	Langjökull	0.30	0.04	0.03	8.3	
ICE-13_21	Snaefellsjökull	1.20	0.10	0.08	12.2	−4.6
ICE-13_24	Snaefellsjökull	1.08	0.10	0.07	10.7	−3.9

The fatty acid distribution was similar in all analyzed snow samples and characterized by predominance of saturated C16 and C18 fatty acids (up to 100%; Table 5 and Table S1). The most abundant unsaturated fatty acids were C16:1, C18:1, C18:2, C18:3. Among these C18:1 was the most prominent fatty acid and the highest proportion of unsaturated fatty acids was found on Drangajökull and Hofsjökull (63–80%), the two glaciers sampled late in the 2012 season. Pigment analysis revealed that chlorophylls (Chl a and Chl b) made up the largest proportion in all samples with a range of 31–100% of total pigments, followed by secondary carotenoids (between 0 and 69%) and primary carotenoids (violaxanthin, zeaxanthin, lutein, β-carotene; up to 8%). Samples in 2012 and 2014 were collected later in the melting season and thus not surprisingly showed higher secondary carotenoids contents (up to 69%). The only secondary carotenoid identified was astaxanthin and the trans-configuration of astaxanthin was prevalent over the cis-configuration and astaxanthin mono esters could also be identified.

The mineralogical analysis of the particulates revealed that the dominant minerals in all samples were quartz, plagioclase (albite, anorthite), and pyroxene with some contributions from clays, basaltic glass and hematite (Figure S1). Hematite was one of the main supersaturated mineral phases in our solutions as shown by the geochemical modeling (Table S7). This bulk mineralogical composition varied little among all collected samples and matches the typical mineralogy of the fresh ash (Jones and Gislason, 2008) and dust from the prime rocks in Iceland, which can be basaltic to rhyolitic (Jakobsson et al., 2008).

### Species composition

**Figure 2:**
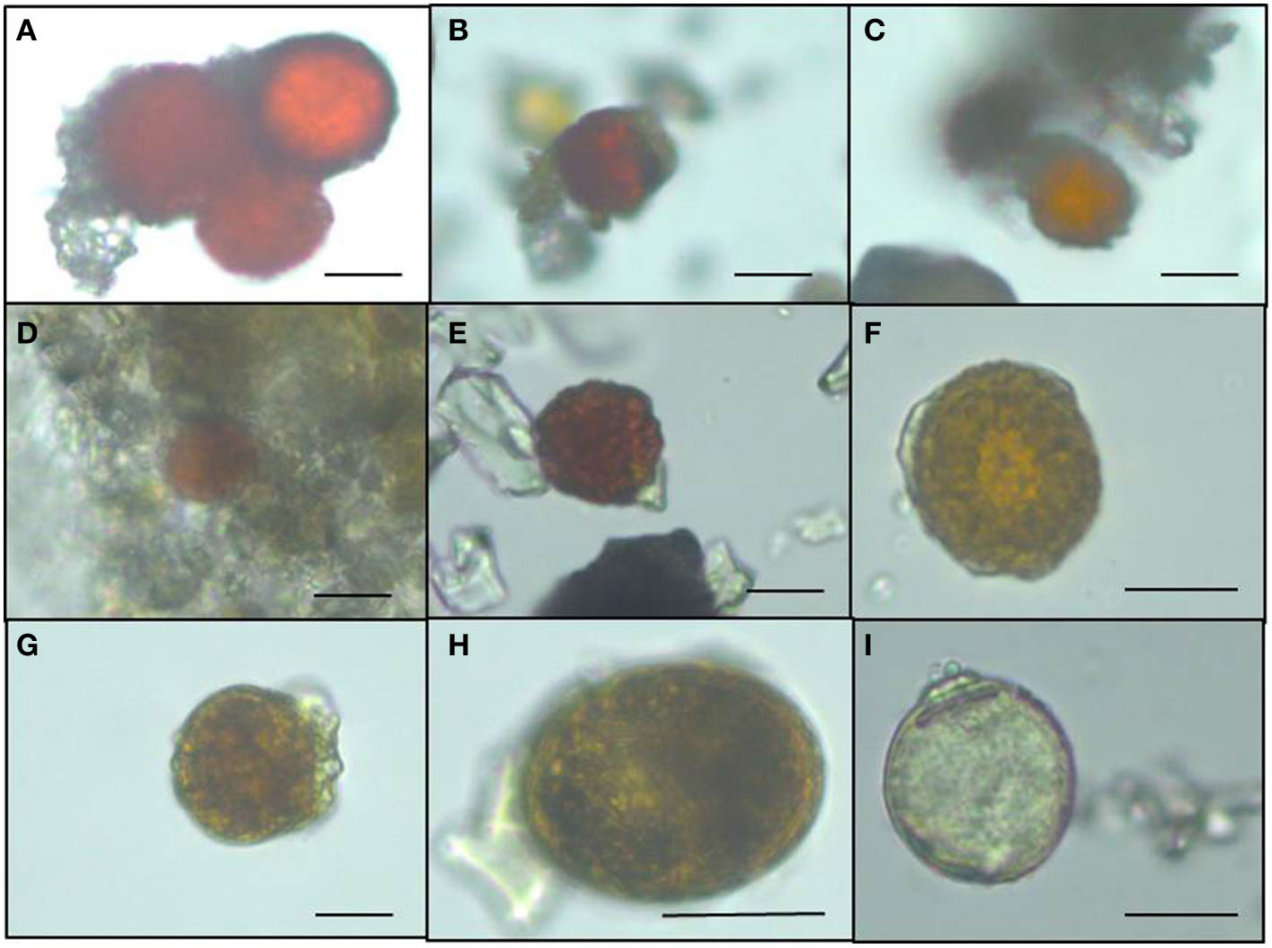
**Light microscopy images of snow algae from the different sampling sites revealing the more red pigmented algae collected in 2012 compared to the less red pigmented algae sampled in 2013. (A)** Drangajökull (ICE-12_3), **(B)** Laugafell (ICE-12_4), **(C)** Hofsjökull (ICE-12_6), **(D)** Snaefellsjökull (ICE-13_2), **(E)** Eyafjallajökull (ICE-13_5), **(F)** Mýrdalsjökull (ICE-13_8), **(G)** Vatnajökull (ICE-13_14), **(H)** Langjökull (ICE-13_16), **(I)** Snaefellsjökull (ICE-13_21).

**Figure 3:**
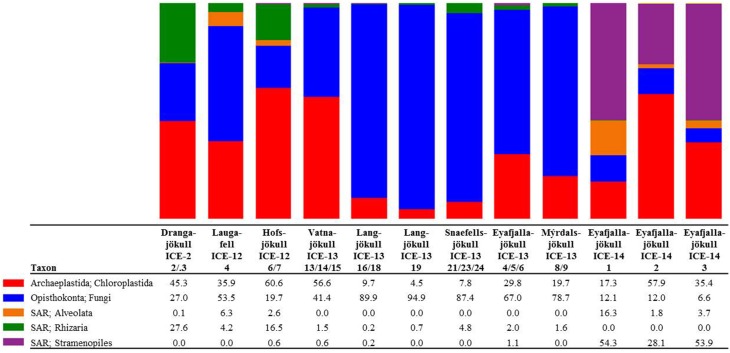
**Distribution of 97% clustered OTUs aligned and assigned to eukaryotes**. Values are the relative abundance of the taxa in percentage of total sequences and figure shows taxa with OTUs of a minimum total observation count of 0.1%. It is important to note that values are rounded to one digit; therefore, the abundance of a taxon with a value of 0.0 in one sample can range between 0.00 and 0.04%. A full OTU table can be found in the SI.

The presence of snow algae was confirmed in all collected samples by light microscopy (Figure 2). Samples from 2012, collected in the late melt season showed overall more red pigmentation, whereas in 2013 samples (collected at the beginning of the melt season) contained more green and yellow pigmented cells (Figure 2 and Table 4). Since algal species identification by microscopy can be deceiving due to various morphological changes during their life stages, targeted DNA sequencing was carried out to reveal algal species composition as well as the full microbial diversity (other micro-eukaryotes, bacteria, archaea) associated with snow algal sites. From all sequences that were amplified with the 18S rRNA primers, a total of 108,790 sequences (12 samples in total; Table 6) passed the QIIME quality pipeline (quality score >20) corresponding to 2811 operational taxonomic units that clustered at 97% sequence identity. Clustering of OTUs at 99, 97, or 95% sequence similarity resulted in differences for OTUs counts (Table S2), however not for taxa assignments and relative abundance of taxa (Tables S3–S5) and therefore, a 97% similarity was chosen to be most representative for all further analyses. OTUs aligned and assigned to our extended Silva database (see Methods) on all phylogenetic levels revealed differences between the eight sampling sites. *Chloroplastida* (green algae) and *Fungi* made up the largest proportion of eukaryotic sequences followed by *Alveolata* (Figure 3). All samples collected in 2013 (except Vatnajökull) showed a much higher abundance of sequences assigned to *Fungi* (67.0–94.9% of total sequences as opposed to 4.5–29.8% of total sequences in 2012, except Laugafell), with the most abundant class represented by the *Microbotryomycetes* (*Basidomycota*) [see full OTU tables in the Supplementary Information (SI) files]. Samples collected in 2012 and 2014 (except Eyafjallajökull, ICE-14_1) showed a higher abundance of C*hloroplastida* (35.4–60.6%; compared to fungi: 6.6–53.5%) and also the presence of *Stramenopiles* (e.g., *Chrysophyceae;* Eyafjallajökull sampled in 2014), *Rhizaria* (e.g., *Cercozoa*; Drangajökull and Hofsjökull sampled in 2012) and *Alveolata* (e.g., *Ciliophora*; Laugafell sampled in 2012 and Eyafjallajökull in 2014). Shannon indices (Table 6) for all eukaryotes varied over a broad range from H′ = 3.75 (Eyafjallajökull, ICE-14_2) to H′ = 6.02 (Laugafell).

**Table 4:** **Pigment composition of red snow samples that contained enough particulate material for analysis**.

**Sample ID**	**Glacier**	**Chl a [μg/L]**	**Chl b [μg/L]**	**Vio [μg/L]**	**Zea [μg/L]**	**Lut [μg/L]**	**β-Car [μg/L]**	**trans- Ast [μg/L]**	**cis-Ast mono esters [μg/L]**	**trans-Ast mono esters [μg/L]**	**Total chlorophylls [%]**	**Total primary carotenoids [%]**	**Total secondary carotenoids [%]**
ICE-12_1	Snaefellsjökull	10,528	1739			552		980		142	88	4	8
ICE-12_3	Drangajökull	4673	12,207		34			580	545		94	0	6
ICE-12_4	Laugafell		5816								100	0	0
ICE-12_7	Hofsjökull		8306					502			94	0	6
ICE-13_5	Eyjafjallajökull	3079				255				4	92	8	0
ICE-13_8	Mýrdalsjökull	2504									100	0	0
ICE-13_15	Vatnajökull	8123	40	67							99	1	0
ICE-13_16	Langjökull	8280								1	100	0	0
ICE-13_21	Snaefellsjökull	20,120		18		250				240	98	1	1
ICE-13_23	Snaefellsjökull	16,016		13		257	13			338	96	2	2
ICE-14_1	Eyjafjallajökull	62	34							157	31	0	69
ICE-14_2	Eyjafjallajökull	87	46							28	78	0	22
ICE-14_3	Eyjafjallajökull	138	69							31	83	0	17

*Individual pigments were quantified in ug/L and reported as total chlorophylls, total primary carotenoids and total secondary carotenoids in % of total pigments*.

**Table 5:** **Fatty acid composition of the red snow samples collected in 2012 and 2013. Fatty acid compounds are reported as percentage of total fatty acids**.

**Compound**	**Glacier**	**C16:0**	**C16:1**	**C18:0**	**C18:1**	**C18:2**	**C18:3**	**SFA**	**MUFA**	**PUFA**	**UFA**	**Ratio SFA/UFA**
ICE-12_2	Drangajökull	20	8	0	58	7	7	20	67	13	80	0.2
ICE-12_3	Drangajökull	18	8	10	52	5	4	31	60	9	69	0.4
ICE-12_5	Laugafell	16	3	57	18	0	0	73	20	0	20	3.6
ICE-12_6	Hofsjökull	18	2	18	53	5	3	37	55	7	63	0.6
ICE-12_7	Hofsjökull	14	4	3	43	27	0	22	48	31	78	0.3
ICE-13_1	Snaefellsjökull	16	16	17	13	6	3	40	29	9	39	1.0
ICE-13_2	Snaefellsjökull	19	12	12	14	11	6	39	26	17	43	0.9
ICE-13_4	Eyafjallajökull	94	0	0	6	0	0	94	6	0	6	16.0
ICE-13_5	Eyafjallajökull	25	8	16	9	12	12	52	17	27	44	1.2
ICE-13_6	Eyafjallajökull	21	2	12	13	10	14	41	15	29	44	0.9
ICE-13_8	Mýrdalsjökull	42	0	56	0	0	0	100	0	0	0	
ICE-13_9	Mýrdalsjökull	38	0	38	16	9	0	75	16	9	25	3.1
ICE-13_12	Vatnajökull	22	5	12	20	6	16	40	25	35	60	0.7
ICE-13_14	Vatnajökull	26	5	11	0	14	29	49	5	44	50	0.98
ICE-13_15	Vatnajökull	26	0	17	16	4	5	60	16	9	25	2.4
ICE-13_16	Langjökull	24	2	10	15	14	15	45	18	36	53	0.8
ICE-13_18	Langjökull	25	4	30	16	9	6	65	20	15	35	1.9
ICE-13_19	Langjökull	33	2	49	5	3	3	88	7	6	12	7.0
ICE-13_21	Snaefellsjökull	27	3	9	21	7	16	44	22	29	51	0.9
ICE-13_24	Snaefellsjökull	28	2	12	16	7	7	61	17	14	31	2.0

Most prominent fatty acids (full table see SI) are reported as well as total saturated (SFA), total monounsaturated (MUFA), total polyunsaturated (PUFA), total unsaturated (UFA) fatty acids, and the ratios of saturated to unsaturated fatty acids.

**Table 6:** **Number of sequences (seqs) for the pooled red snow samples for each glacier and the respective Shannon diversity index (H′)**.

**Glacier**	**Pooled**	**Eukaryotes**					**Bacteria**				**Archaea**		
	**samples**	**Raw seqs**	**Seqs after QC**	**H′ euk**	**Seqs assigned to algae**	**H′ algae**	**Raw seqs**	**Seqs after QC**	**Seqs assigned to bac**	**H′ bac**	**Raw seqs**	**Seqs after QC**	**Seqs assigned to arch**
Drangajökull	ICE-12_2/3	5474	2013	5.55	714	4.50	6383	2333	513	5.13	9206	1443	4^*^
Laugafell	ICE-12_4	1908	597	6.02	89	4.47	2533	818	120	5.27	18323	6294	540
Hofsjökull	ICE-12_6/7	6523	2168	5.61	792	3.88	3105	1336	339	5.38	18956	623	33
Vatnajökull	ICE-13_13/14/15	9948	3438	5.02	66	4.51	2689	770	14	5.06	4034	646	210
Langjökull	ICE-13_16/18	318	104	5.65	340	4.26	2398	913	111	5.29	5675	643	334
Langjökull	ICE-13_19	7273	3065	4.99	38	4.26	6541	2153	444	5.14	14680	4129	77
Snaefellsjökull	ICE-13_21/23/24	5123	1588	5.51	41	4.04	5850	2032	486	3.97	9800	3072	338
Eyafjallajökull	ICE-13_4/5/6	2391	1072	5.14	37	4.21	12075	2897	55	5.27	10862	2268	0^*^
Mýrdalsjökull	ICE-13_8/9	2479	809	5.14	8	n.d.	406	97	2^*^	n.d.	14760	5426	3^*^
Eyafjallajökull	ICE-14_1	34082	23959	5.11	3357	1.74	3842	1884	938	4.53	85138	66535	65727
Eyafjallajökull	ICE-14_2	8716	6962	3.75	3307	1.07	3875	2145	1158	4.64	841	809	558
Eyafjallajökull	ICE-14_3	24555	17196	4.96	4460	1.81	13230	6843	3390	4.59	28203	22350	21572

* Removed from analysis due to low sequence numbers, n.d., not determined due to low sequence numbers.

**Figure 4:**
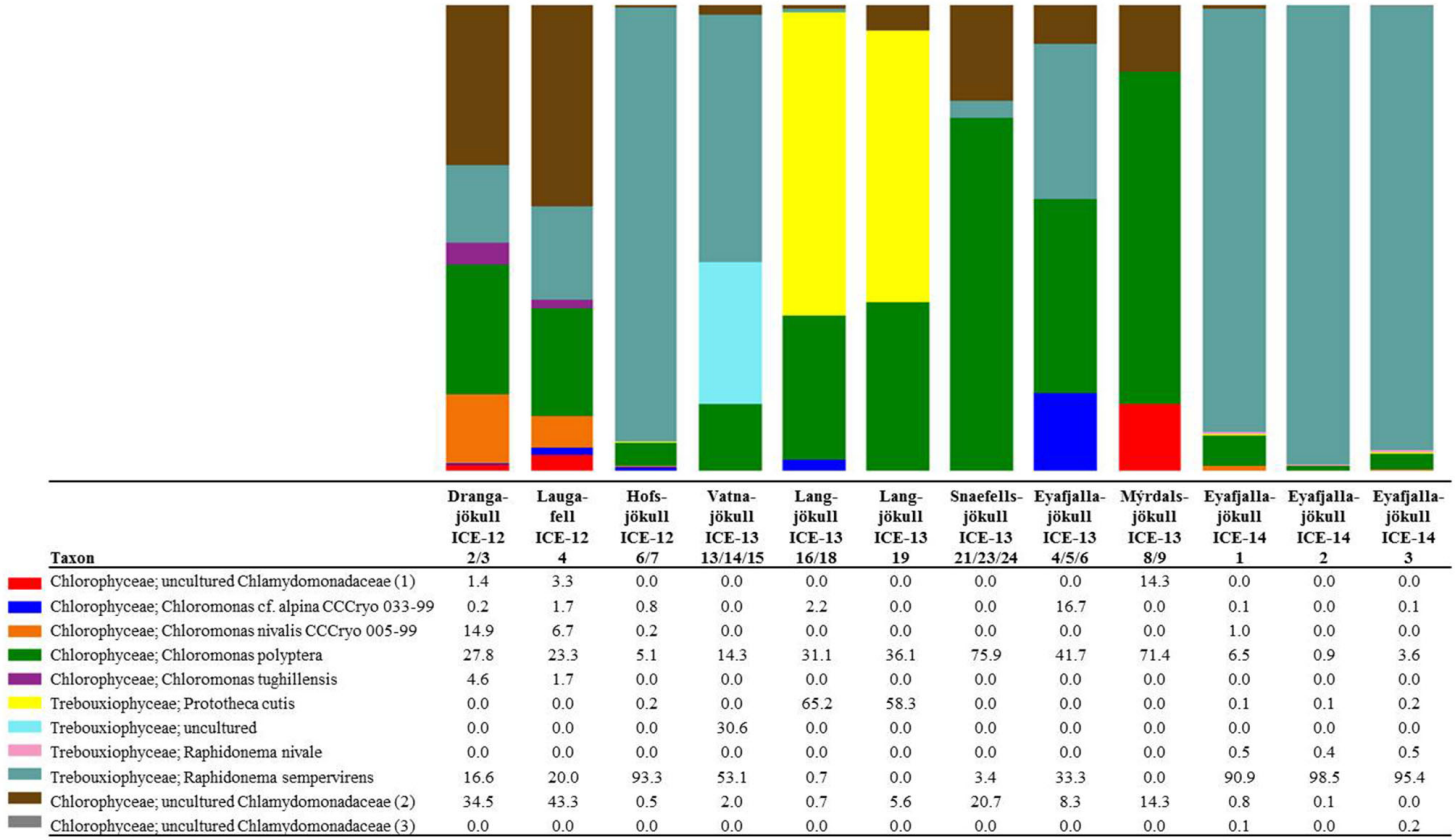
**Distribution of 97% clustered OTUs aligned and assigned to known algal species**. Values are the relative abundance of the taxa in percentage of total sequences and figure shows taxa with OTUs of a minimum total observation count of 0.05%. It is important to note that values are rounded to one digit; therefore, the abundance of a taxon with a value of 0.0 in one sample can range between 0.00 and 0.04%. A full OTU table can be found in the SI.

**Figure 5:**
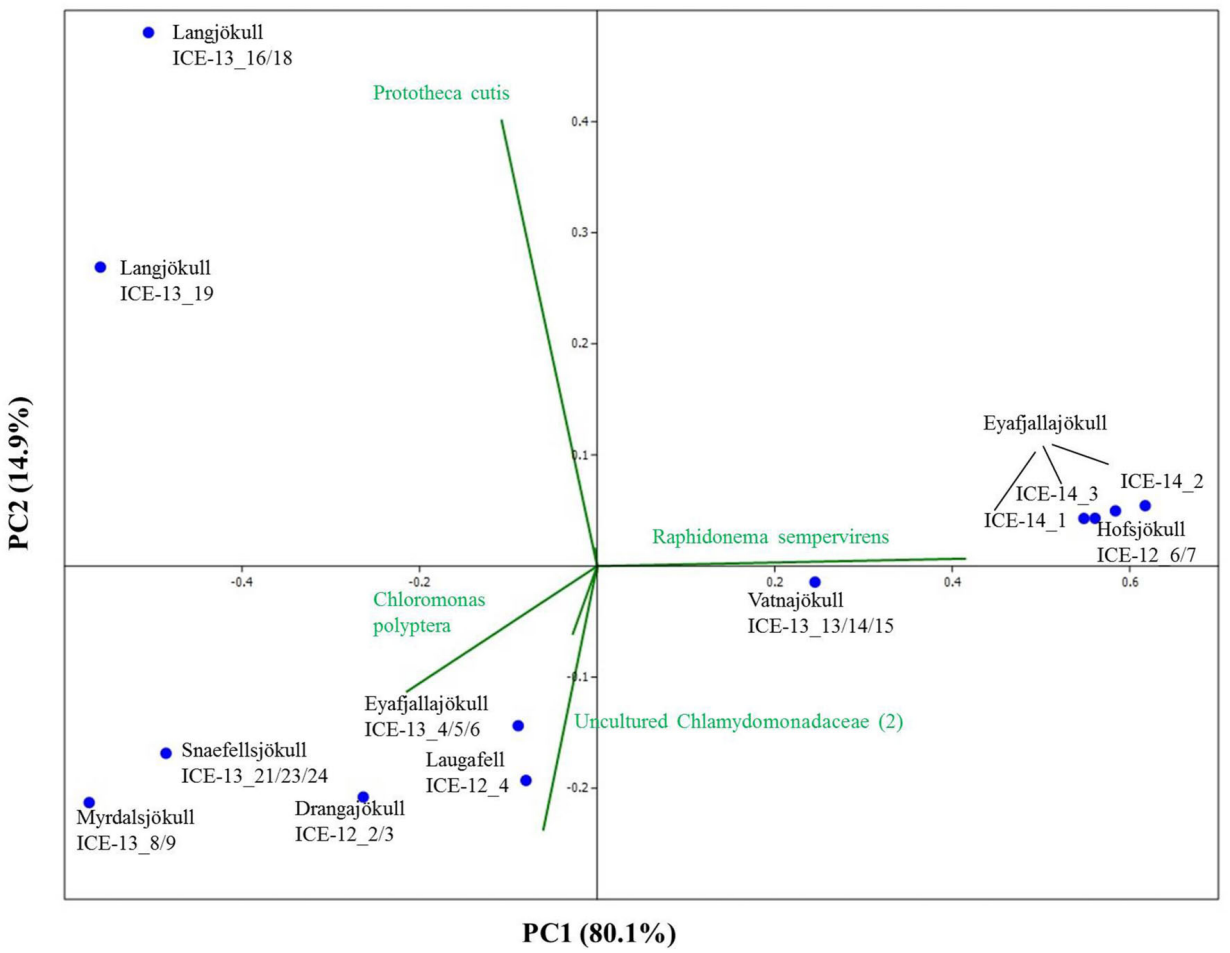
**Principal component analysis of algal species revealing taxonomic distance between sampling sites and species causing separation**. However, taxonomic distance cannot be explained by increasing geographic distance or collection time.

In order to investigate algal relative abundance all sequences corresponding to *Chloroplastida* were filtered from the main OTU table (Figure 4) with 567 OTUs remaining. Sequences matching *Embryophyta* showed low abundance with <7% for all samples with Vatnajökull being the exception and a value of 22% of total eukaryotic sequences. All sequences matching *Embryophyta* were removed from further analyses. The most abundant genera of algae belong to the *Chlamydomonadaceae* with *Chloromonas polyptera* being the dominant taxon. Two uncultured *Chlamydomonadaceae* species were also highly abundant and based on their 18S rRNA sequences they shared the highest sequence similarity (89–93% similarity) with other *Chloromonas* species found in our samples (Figure S2). The *Trebouxiaceae* were represented by *Raphidonema sempervirens* as the dominant taxon. Other *Chloromonas* species with intermediate abundance (up to 16.7%) were *Chr. nivalis*, *Chr. alpina* and *Chr. tughillensis*. Relative abundance of *Chlamydomonas*, *Ancylonema*, and *Mesotaenium*, that are typically described on glacial surfaces worldwide, was very low (<0.1%). In the Langjökull sample we also found a high number of sequences matching *Prototheca cutis*, a newly discovered pathogenic algae (Satoh et al., 2010), that may be derived from sledge dog feces that was abundant close to our sampling site. Full OTU tables can be found in the SI files. Shannon indices (Table 6) for algal species did not reveal large differences between sites (H′ = 3.88–4.51). The exceptions were the three samples collected from Eyafjallajökull in 2014, which showed a much lower diversity in the algae species (H′ = 1.07–1.81). The PCA analysis of the algal species (Figure 5) revealed taxonomic distance between sampling sites, however, separation was not caused by increasing geographic distance or collection time.

**Figure 6:**
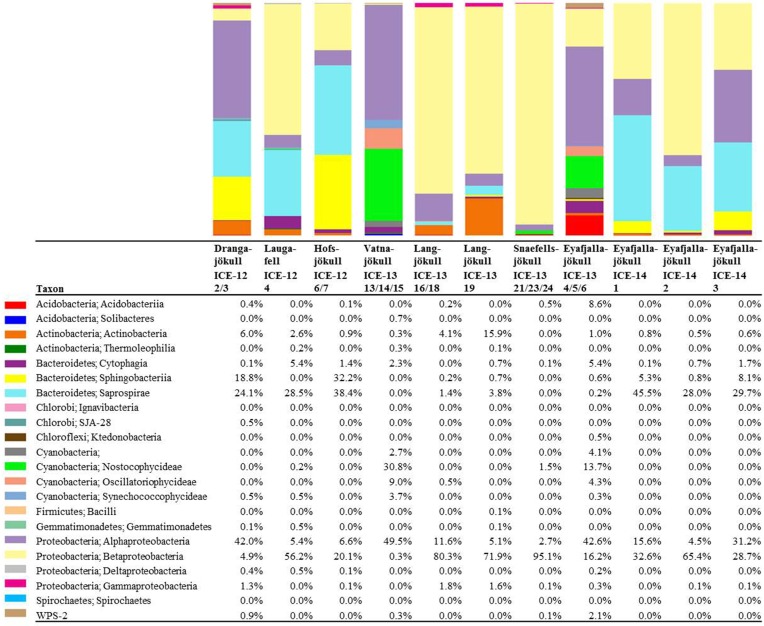
**Distribution of 97% clustered OTUs aligned and assigned to known bacterial species**. Values are the relative abundance of the taxa in percentage of total sequences and figure shows taxa with >0.01% abundance. It is important to note that values are rounded to one digit; therefore, the abundance of a taxon with a value of 0.0 in one sample can range between 0.00 and 0.04%. A full OTU table can be found in the SI.

**Figure 7:**
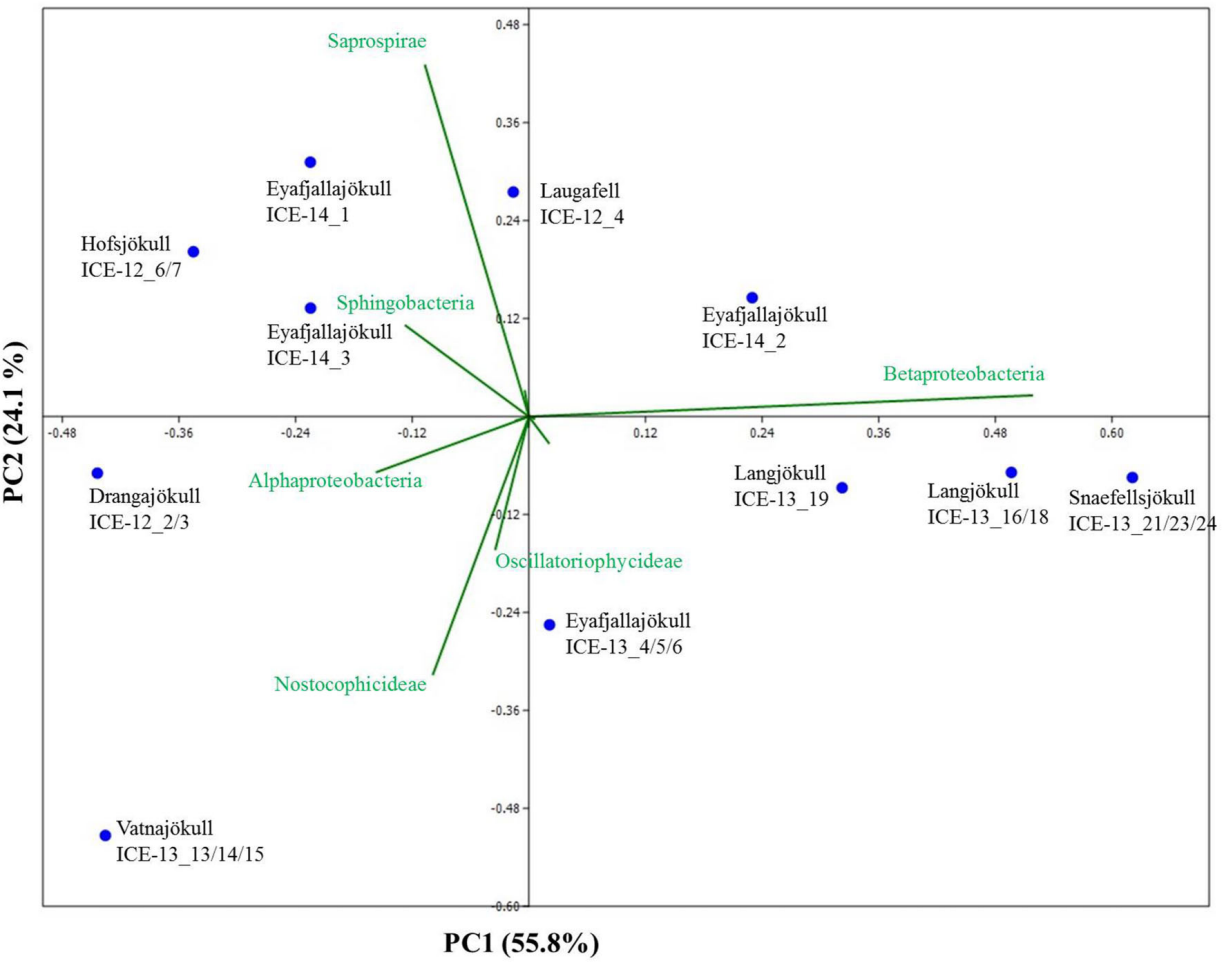
**Principal component analysis of bacterial species revealing taxonomic distance between sampling sites and species causing separation**. Samples collected in 2012 and 2014 cluster together due to a higher relative abundance of *Bacteriodetes* (*Sphingobacteria*, *Saprospirae*), whereas samples collected in 2013 contain higher proportions of *Betaproteobacteria* and *Cyanobacteria* (*Nostocophicidae*, *Oscillatoriophycideae*).

Bacterial primer amplification resulted in 24,221 sequences (12 samples in total) passing the QIIME quality pipeline corresponding to 1733 operational taxonomic units clustered at 97% sequence identity. Again similar values were derived when the relative abundance of taxa for OTUs were clustered at different similarities of 99, 97, and 95% (Table S5). OTUs aligned and assigned to the Greengenes database revealed differences between the eight sampling sites. The most abundant bacterial phyla were *Proteobacteria*, *Bacteriodetes*, and *Cyanobacteria* (see Figure 6). Within the *Proteobacteria*, *Betaproteobacteria* were most abundant followed by *Alphaproteobacteria*. *Betaproteobacteria* were present in high abundance on Snaefellsjökull (95.1%) in 2012, Langjökull in 2013 (80.3 and 71.9%) and Eyafjallajökull in 2014 (28.7–65.4%). In contrast, *Alphaproteobacteria* were most abundant on Vatnajökull (49.5%) and Eyafjallajökull in 2013 (42.6%), and Drangajökull (42.0 %) in 2012. Within the *Bacteriodetes*, the *Sphingobacteria*, and *Saprospirae* were the most abundant representative classes in the samples collected in 2012 and 2014. *Sphingobacteria* showed higher relative abundance on Hofsjökull (32.2%) and on Drangajökull (18.8%) whereas *Saprospirae* were more present on Hofsjökull (38.4%), in the three samples collected from Eyafjallajökull in 2014 (28.0–45.5%), Laugafell (28.5%) and Drangajökull 24.1%). *Cyanobacteria* (*Nostocophycideae* and *Oscillatoriophycideae*) were strongly represented only on Eyafjallajökull (74.0%), Vatnajökull (56.4%), and Langjökull (24.4%) collected in 2013. The Shannon indices for most bacterial samples (Table 6) varied over a narrow range (H′ = 5.13–5.38) and showed the same trend as for algae with similar values for all glaciers. Exceptions were again the three samples collected from Eyafjallajökull in 2014 (4.52–4.64) and the pooled Snaefellsjökull sample collected in 2013, which had the lowest bacterial diversity index among all bacterial samples (H′ = 3.97). PCA analysis (Figure 7) showed samples collected in 2012 and 2014 clustering together due to higher relative abundance of *Bacteriodetes* (*Sphingobacteria, Saprospirae*), whereas samples collected in 2013 clustered together due to higher proportions of *Betaproteobacteria* and *Cyanobacteria* (*Nostocophicidae, Oscillatoriophycideae*).

Archaea were detected in most snow samples. For samples collected in 2013 and 2014 ~ 80% of all sequences could not be assigned to archaeal species after passing the QIIME quality pipeline and were removed from the analysis. Samples with only very few sequences left (*n* < 10) were completely removed from the analysis and only six samples were further analyzed. For samples collected in 2014 and sequenced on a #316v2 chip (see Methods), we gained 114,182 raw sequences and 89,694 sequences passed the QIIME quality pipeline. For both sequencing runs and independent from the large variation in sequence numbers, the most striking feature is that the archaeal species diversity is very low and dominated by 1–2 species only. The dominant phyla (>98% of all sequences) on most glaciers (Laugafell, Vatnajökull, Langjökull, Snaefellsjökull) belong to the *Nitrososphaerales* (*Thaumarchaeota*). Only on Hofsjökull (ICE-12_6/7) *Methanosarcinales* (*Euryarchaeota*) were found in higher abundance (71.6%) than *Nitrososphaerales*, and in one of the 2014 Eyafjallajökull samples (ICE-14_2) the *Cenarchaeales* (*Thaumarchaeota*) were found to make up 18.1% of the abundance besides the dominant *Nitrososphaerales*.

## Discussion

### Microbial diversity

To our knowledge, this is the first time that microbial diversity in general and snow algae in particular have been described on Icelandic glaciers and ice caps.

#### Eukaryotic communities

Snow algae were present and abundant on all studied glaciers and ice caps. The algal species diversity was in all cases very low and only four phylotypes with highest sequence similiarity to *Chloromonas polyptera*, *Raphidonema sempervirens*, and two uncultured *Chlamydomonadaceae* comprised >95% of the total sequences in all our samples. This is in agreement with Leya (2004) who also described low algal diversity, with 2–3 species making up >95% of the snow algal community, on glaciers from Svalbard. It is worth noting however, that all available 18S rRNA gene data targeting snow algae are based on culture-dependent studies and clone libraries entailing a high degree of bias and a limited sequencing depth, respectively. Therefore, a direct comparison with the few previous studies that targeted snow algae communities (e.g., Leya et al., 2004; Remias et al., 2013) is difficult although all suggest low diversity. Furthermore, it is also well-known that snow algae can dramatically change their morphologies during their life cycles (Müller et al., 2001). This makes classifications and inter-study comparisons based on microscopy very challenging and over the course of the last decades many snow algal species have been re-classified in some cases even several times (personal communication from Dr. Thomas Leya). For this reason, the most notable snow algal taxon *Chlamydomonas nivalis* needs to be treated as a collective taxon and not as a single species (Leya et al., 2004).

The most dominant species in our samples, *Chloromonas polyptera* has so far only been described from coastal Antarctic snow fields in the vicinity of penguin rockeries where this species has been identified based on microscopy and clone library sequencing of the 18S rRNA gene (Remias et al., 2013). This taxon is known to share many cryophilic strategies with the more famous snow algae species *Chloromonas nivalis* (Remias et al., 2013). These strategies include the formation of cyst stages and accumulation of the protective carotenoid astaxanthin. The two uncultured *Chlamydomonadaceae* species (labeled as *Chlamydomonadaceae* 1 and *Chlamydomonadaceae* 2 in Figure 4) are abundant in the Alps and are also known for the formation of cyst stages (personal communication Thomas Leya). However, not much is known about their physiology, since culturing efforts have not been successful so far. Based on their 18S rRNA they show the highest sequence similarity (89–93% similarity) with other *Chloromonas* species found in our samples (Figure S2).

The second most abundant species *Raphidonema sempervirens* (Figure 4) is better known as a typical permafrost algae and is not a true snow algae species. Laboratory experiments (Leya et al., 2009) also demonstrated that *Raphidonema sempervirens* does not share one of the main cryophilic properties of true snow algae, i.e., the production of secondary carotenoids (e.g., astaxanthin). Yet, in culture and under optimal conditions *Raphidonema sempervirens* is able to produce significant amounts of primary carotenoids (xanthophylls; Leya et al., 2009). In our samples, we only detected relatively minor amounts of the xanthophylls violaxantin and lutein (Table 4) besides chlorophyll and the secondary carotenoid astaxantin. However, analyses of natural snow algae samples revealed that pigment distributions are most often highly variable and dependent on sampling time and location. For example, in Lutz et al. (2014) we have shown that the pigment composition on a single glacier dramatically changed during a 2 week melting season. Furthermore, Stibal and Elster (2005) have suggested that *Raphidonema sempervirens* is likely introduced onto glacial surfaces by wind rather than through *in-situ* propagation. Thus, despite its high abundance in some of our samples (e.g., 93.3% in Hofsjökull in 2012 and 90.9–98.5% in Eyafjallajökull in 2014) it remains unclear whether this species plays an active role in the ecology of Icelandic glaciers and elsewhere.

All samples collected early in the melt season in 2013 (except Vatnajökull) showed a much higher relative abundance of sequences assigned to *Fungi* (67.0–94.9% of total sequences), with the most abundant class represented by the *Microbotryomycetes* (*Basidomycota*), in comparison to *Chloroplastida* (4.5–29.8%). The higher relative abundance of fungi in our samples could be due to the earlier sampling time (beginning of June in 2013 compared to end of July in 2012 and end of August in 2014) and thus before the onset of melting, which initiates the bloom of snow algal communities. In contrast, samples collected in 2012 and 2014 showed a higher relative abundance of *Chloroplastida* (35.4–60.6%; fungi: 6.6–53.5%). They also confirmed the presence of *Stramenopiles* (e.g., *Chrysophyceae*; Eyafjallajökull 2014 samples), *Rhizaria* (e.g., *Cercozoa*; Drangajökull and Hofsjökull 2012 samples) and *Alveolata* (e.g., *Ciliophora*; Laugafell and Eyafjallajökull in 2014), which were only found in considerable abundances where snow algal sequences were also abundant. Their presence may support the importance of snow algal communities as primary colonizers, producers of organic carbon and nutrient sources for other microbial communities.

#### Bacterial communities

A comparison with previous bacterial studies of snow is easier since more 16S rRNA gene studies have been published so far. However, these mostly targeted relatively fresh spring snow (Larose et al., 2010) or clean summer snow (Cameron et al., 2014). In our study, we targeted bacteria in summer snow that were associated with snow algal communities. Yet, considering that algal diversity is limited to very few taxa, we could not find a match between bacterial and algal species composition (Figures 5, 7). Likewise for algae, bacterial species compositions from samples collected late in the melt season (August 2012 and 2014) compared to early in the season (June 2013) suggest a likely seasonal effect. Specifically, *Betaproteobacteria* were more abundant in sequence data from earlier in the melt season (2013 samples) whereas *Bacteroidetes* were more abundant later in the season (2012 and 2014 samples). In other studies a high relative abundance of *Proteobacteria* and *Bacteroidetes* has often been found in snow and ice samples not associated with algal blooms. For example, a high abundance of *Proteobacteria* was found in snow in Greenland (Cameron et al., 2014), in snow and ice in China (Segawa et al., 2014), in spring snow in Svalbard (Larose et al., 2010), in snow, slush and surface ice in Svalbard (Hell et al., 2013), and in cryoconite holes in the Alps and Svalbard (Edwards et al., 2013, 2014). Furthermore, in previous studies *Bacteroidetes* also showed a higher relative abundance in cryoconite holes (Edwards et al., 2013, 2014).

#### Archaeal communities

We were also able to confirm the presence of archaea in our samples. Currently, only very few studies have documented the presence of archaea in glacial environments. They have been found in a glacial stream in Austria (Battin et al., 2001), in subglacial sediments in Canada (Boyd et al., 2011) and in cryoconite holes in Antarctica (Cameron et al., 2012) and Svalbard (Zarsky et al., 2013). Our study revealed a very limited diversity, with *Nitrososphaerales* (*Thaumarchaeota*) and *Methanosarcinales (Euryarchaeota)* being the only archaeal taxa present (Table 7), consistent with the earlier studies. Cameron et al. (2012) similarly found a limited number of taxa affiliated with *Thaumarchaeaota* and *Methanobacteriaceae* restricted to Antarctic cryoconite. Cameron et al. (2014) reported similar low archaeal diversity in four snow samples collected between 1.6 and 9.5 km from the margin of the Greenland Ice Sheet. Therefore, when taken into consideration jointly, these studies offer a consensus that the apparent diversity of Archaea on glacier surfaces is low. *Nitrososphaerales* may play an important role in nitrogen cycling contributing toward ammonia oxidation and nitrification (Tourna et al., 2011; Zarsky et al., 2013; Stieglmeier et al., 2014). However, in order to fully explore such links further detailed analyses are needed.

**Table 7:** **Distribution of 97% clustered OTUs aligned and assigned to archaea in analyzed red snow samples, revealing a dominance of the two phyla *Nitrososphaerales* (*Thaumarchaeota*) and *Methanosarcinales (Euryarchaeota)***.

**Taxon**	**Laugafell**	**Hofsjökull**	**Vatnajökull**	**Langjökull**	**Langjökull**	**Snaefellsjökull**	**Eyafjallajökull**	**Eyafjallajökull**	**Eyafjallajökull**
	**ICE-12 4/5**	**ICE-12 6/7**	**ICE-13 11–15**	**ICE-13 16–18**	**ICE-13 19**	**ICE-13 21–24**	**ICE-14 1**	**ICE-14 2**	**ICE-14 3**
Crenarchaeota; MBGA	0.0	0.0	0.0	0.0	0.0	0.2	0.0	0.0	0.0
Crenarchaeota; Thaumarchaeota; Cenarchaeales; Cenarchaeaceae	2.8	0.7	7.7	0.3	1.1	4.2	0.0	0.0	0.0
Crenarchaeota; Thaumarchaeota; Cenarchaeales; SAGMA-X	0.0	0.0	0.0	0.0	0.5	0.0	0.0	18.1	0.0
Crenarchaeota; Thaumarchaeota; Nitrososphaerales; Nitrososphaeraceae	96.5	27.6	92.0	99.7	98.1	95.6	100.0	78.6	99.9
Euryarchaeota; Methanobacteria; Methanobacteriales; MSBL1	0.0	0.0	0.0	0.0	0.0	0.0	0.0	0.0	0.0
Euryarchaeota; Methanomicrobia; Methanosarcinales; Methanosarcinaceae	0.0	71.6	0.0	0.0	0.0	0.0	0.0	3.2	0.0

It is important to note that values are rounded to one digit; therefore, the abundance of a taxon with a value of 0.0 in one sample can range between 0.00 and 0.04%.

### Environmental parameters

In order to investigate the environmental parameters controlling snow algal species distribution we analyzed a large suite of physical and chemical parameters in all collected snow samples. We quantified aqueous nutrient and trace metal contents as well as particulate nutrient abundances. Our geochemical modeling (Table S7) showed that nutrients and trace metals varied over a narrow range, and were in equilibrium with the nutrient-rich and fast dissolving ubiquitously present volcanic ash (Dagsson-Waldhauserova et al., 2015) which likely supports snow algal communities to thrive. However, we could not establish any relevant differences between sites of red and clean snow. Spijkerman et al. (2012) also could not find a relation between dissolved and particulate nutrients in red snow samples in Svalbard.

Nitrogen is often the most important nutrient for microbial growth. Particulate d^15^N results showed throughout negative values ranging from −11.2 to −3.9‰ suggesting an atmospheric nitrogen source for all samples. This indicates that the source of nitrogen is very similar for all glaciers and not a selecting factor for snow algal and bacterial distribution. Other studies have identified fecal pellets from bird colonies as the main primary source, which would lead to more recycled nitrogen and thus more positive nitrogen isotopic values (Fujii et al., 2010). Analysis of the particulate carbon to nitrogen ratios (C/N) revealed nitrogen limiting conditions (C/N > 6.6, Redfield ratio) for Langjökull, Snaefellsjökull and Eyafjallajökull and non-limiting conditions (C/N < 6.6) for Hofsjökull, Mýrdalsjökull and Vatnajökull. Although in this study particulate carbon was analyzed as total carbon (TC), the largest proportion is likely to be organic carbon since no carbonates were found in the XRD analysis and overall carbonates are highly unlikely in basaltic rocks. Nevertheless, overall the total carbon and nitrogen contents and C/N ratios in our solid samples are similar to most values measured in other glacial communities such as snow algae in Svalbard (C/N: 16–33; Spijkerman et al., 2012), cryoconite holes on a Himalayan Glacier (C: 2.7%, N: 0.27%, C/N: 10; Takeuchi et al., 2001) and in cryoconite holes in Svalbard (C: up to 4%, N: up to 0.4%, C/N 12.5; Stibal et al., 2006).

We could not identify patterns for any of the analyzed environmental parameters to explain differences in species composition between glaciers. However, we may not have captured all parameters and there may be trends for overall biomass. Furthermore, the extend of melting and stage in the melt season at the time of collection may play a more important role and needs to be investigated in future studies.

### Metabolic inventory

Snow algae have evolved a well-adapted physiology and metabolism in order to thrive in glacial environments (Remias et al., 2005; Leya et al., 2009). Fatty acids play an essential role as structural elements of their membranes and as storage compounds (Thompson, 1996). The relative composition of fatty acids depends on environmental factors such as temperature, irradiation and nutrient availability (Piorreck et al., 1984; Roessler, 1990), but also varies between species (Spijkerman et al., 2012). In our samples we found mainly the two common saturated C16 and C18 fatty acids, but also unsaturated C16 and C18 compounds. Temperature is one of the main factors that affect the fatty acid composition, with a general trend toward increasing unsaturation with decreasing temperatures. However, in this study, temperature effects can be neglected since measured snow temperatures varied by less than 1°C (Table 1). Therefore, we contend differences in fatty acid abundance more likely originate from varying nutrient concentrations. Piorreck et al. (1984) found a positive correlation between nitrogen concentrations and fatty acid production of lab cultures of green algae and they showed that a high production of polyunsaturated fatty acids (PUFA) occurred at high N concentrations, whereas at lower concentrations there was a shift toward a higher relative abundance of C16:0 and C18:1. Spijkerman et al. (2012) also reported an increase in C18:1 production of lab cultures with decreasing nitrogen concentrations. One explanation may be a metabolism shifted toward nitrogen free non-protein compounds with nitrogen deficiencies.

Fatty acids are also often linked to pigments and astaxanthin has been shown to be associated with higher amounts of C18:1 fatty acids (Řezanka et al., 2008). The same trend could be found in our samples with higher astaxanthin contents in samples collected in 2012 toward the end of the melt season and also the highest relative abundance of C18:1. Astaxanthin is one of the main pigments causing an intensive red coloration of snow algal cells. Samples collected in 2012 showed overall more red pigmentation, potentially due to the collection date being toward the end of the melt season and longer exposure periods to stress (e.g., irradiation), whereas in 2013 samples were collected earlier in the season and showed more green and yellow pigmented cells (see Figure 2 and Table 4). Astaxanthin was primarily found in samples collected in the 2012 and 2014 field campaigns, which were carried out later in the melt season after longer periods of higher irradiation. This also matches our findings in Greenland where we followed pigment development over a three-week period and found increasing amounts of astaxanthin while the melting progressed (Lutz et al., 2014). In their samples from Antarctica, Remias et al. (2013) found much higher secondary carotenoid contents (51%) for *Chloromonas polyptera*, which was also one of the dominant species in our study. The lower secondary carotenoid content in our samples that were dominated by *Chloromonas polyptera* could be due to lower stress levels in Iceland (e.g., less excessive irradiation) or the high content of *Raphidonema sempervirens* contributing to the total pigment content and which is not known to produce these pigments (Leya et al., 2009). It is important to mention that a contribution of pigmentation derived from *Embryophyta* (mainly Chl a) to the total analyzed pigment composition cannot be excluded.

The pigmentation may also be linked to the observed decrease in albedo from clean snow (76% ± 8) to sites where we observed algal colonization (56% ± 14). In Iceland, the most common considered component of albedo change is the volcanic ash and the combination of black ash and colored algae affect albedo measurements dramatically. A quantitative evaluation of the algal contribution to the observed decrease in albedo is still lacking and needs to be further investigated. However, the observed reduction in albedo at our algal sampling sites (Table 1) matches our previous observations in Greenland using the same approach (Lutz et al., 2014).

In conclusion, we show that snow algae are abundant on all major Icelandic glaciers and ice caps with a rich community comprising of other micro-eukaryotes, bacteria, and archaea. Snow algal pigmentation and volcanic ash are causing a reduction of surface albedo, which in turn could potentially have an impact on the melt rates of Icelandic glaciers.

### Conflict of interest statement

The authors declare that the research was conducted in the absence of any commercial or financial relationships that could be construed as a potential conflict of interest.
