# Supplementary material for: Microbial diversity on Icelandic glaciers and ice caps
Source: Front Microbiol. 2015 Apr 20;6:307. doi: 10.3389/fmicb.2015.00307 (PMC4403510; doi:10.3389/fmicb.2015.00307)
Supplement: Supplementary file 1 [file DataSheet1.DOCX]

**Supplementary Information**

Table S1: Fatty acid composition of red snow samples. Fatty acid compounds are reported as percentage of total fatty acids. b=branched, A = Alkane.

| Compound | C14:0 | C15 b | C15:0 | C16:4 | C15  b | C16:1 | C16:1 | C16:1 | C16:0 | C18:3 | C18:4 | C18:2 | C18:3 | C18:1 | C18:1 | C21  A | C18:0 | C20:5 | C23 A | C20:0 | C21:0 | C22:0 | C26  A | C27  A | C24:0 |
| --- | --- | --- | --- | --- | --- | --- | --- | --- | --- | --- | --- | --- | --- | --- | --- | --- | --- | --- | --- | --- | --- | --- | --- | --- | --- |
| ICE-12_2 | 0 | 0 | 0 | 0 | 0 | 8 | 0 | 0 | 20 | 0 | 0 | 7 | 7 | 46 | 12 | 0 | 0 | 0 | 0 | 0 | 0 | 0 | 0 | 0 | 0 |
| ICE-12_3 | 0 | 0 | 0 | 0 | 0 | 8 | 0 | 0 | 18 | 0 | 0 | 5 | 4 | 47 | 5 | 0 | 10 | 0 | 0 | 2 | 0 | 0 | 0 | 0 | 0 |
| ICE-12_5 | 0 | 0 | 0 | 0 | 0 | 0 | 3 | 0 | 16 | 0 | 0 | 0 | 0 | 18 | 0 | 7 | 57 | 0 | 0 | 0 | 0 | 0 | 0 | 0 | 0 |
| ICE-12_6 | 2 | 0 | 0 | 0 | 0 | 2 | 0 | 0 | 18 | 0 | 0 | 5 | 3 | 50 | 2 | 0 | 18 | 0 | 0 | 0 | 0 | 0 | 0 | 0 | 0 |
| ICE-12_7 | 3 | 0 | 2 | 0 | 0 | 4 | 0 | 0 | 14 | 0 | 4 | 27 | 0 | 43 | 0 | 0 | 3 | 0 | 0 | 0 | 0 | 0 | 0 | 0 | 0 |
| ICE-13_1 | 0 | 6 | 6 | 0 | 0 | 16 | 0 | 0 | 16 | 0 | 0 | 6 | 3 | 8 | 5 | 0 | 17 | 0 | 0 | 0 | 0 | 0 | 0 | 0 | 0 |
| ICE-13_2 | 1 | 0 | 0 | 0 | 0 | 12 | 0 | 0 | 19 | 0 | 0 | 11 | 6 | 8 | 6 | 1 | 12 | 0 | 1 | 2 | 0 | 3 | 0 | 2 | 2 |
| ICE-13_4 | 0 | 0 | 0 | 0 | 0 | 0 | 0 | 0 | 94 | 0 | 0 | 0 | 0 | 6 | 0 | 0 | 0 | 0 | 0 | 0 | 0 | 0 | 0 | 0 | 0 |
| ICE-13_5 | 5 | 0 | 0 | 0 | 0 | 8 | 0 | 0 | 25 | 0 | 2 | 12 | 12 | 9 | 0 | 0 | 16 | 0 | 0 | 3 | 0 | 4 | 0 | 0 | 0 |
| ICE-13_6 | 3 | 0 | 0 | 0 | 0 | 2 | 0 | 0 | 21 | 2 | 4 | 10 | 13 | 13 | 0 | 7 | 12 | 1 | 2 | 3 | 0 | 3 | 0 | 0 | 0 |
| ICE-13_8 | 2 | 0 | 0 | 0 | 0 | 0 | 0 | 0 | 42 | 0 | 0 | 0 | 0 | 0 | 0 | 0 | 56 | 0 | 0 | 0 | 0 | 0 | 0 | 0 | 0 |
| ICE-13_9 | 0 | 0 | 0 | 0 | 0 | 0 | 0 | 0 | 38 | 0 | 0 | 9 | 0 | 16 | 0 | 0 | 38 | 0 | 0 | 0 | 0 | 0 | 0 | 0 | 0 |
| ICE-13_12 | 0 | 0 | 0 | 5 | 0 | 5 | 0 | 0 | 22 | 0 | 4 | 6 | 16 | 20 | 0 | 0 | 12 | 4 | 0 | 0 | 0 | 3 | 0 | 0 | 3 |
| ICE-13_14 | 3 | 0 | 0 | 0 | 0 | 5 | 0 | 0 | 26 | 0 | 0 | 14 | 29 | 0 | 0 | 0 | 11 | 1 | 0 | 3 | 0 | 4 | 0 | 0 | 2 |
| ICE-13_15 | 2 | 0 | 0 | 0 | 0 | 0 | 0 | 0 | 26 | 0 | 0 | 4 | 5 | 16 | 0 | 3 | 17 | 0 | 4 | 5 | 0 | 6 | 1 | 4 | 4 |
| ICE-13_16 | 4 | 0 | 0 | 0 | 0 | 2 | 0 | 0 | 24 | 2 | 5 | 14 | 13 | 15 | 0 | 0 | 10 | 2 | 0 | 2 | 2 | 2 | 0 | 0 | 2 |
| ICE-13_18 | 2 | 0 | 0 | 0 | 0 | 4 | 0 | 0 | 25 | 0 | 0 | 9 | 6 | 16 | 0 | 0 | 30 | 0 | 0 | 2 | 1 | 3 | 0 | 0 | 2 |
| ICE-13_19 | 2 | 0 | 0 | 0 | 0 | 2 | 0 | 0 | 33 | 0 | 0 | 3 | 3 | 5 | 0 | 0 | 49 | 0 | 0 | 2 | 0 | 2 | 0 | 0 | 0 |
| ICE-13_21 | 1 | 0 | 0 | 6 | 1 | 1 | 0 | 1 | 27 | 0 | 0 | 7 | 16 | 21 | 0 | 0 | 9 | 0 | 0 | 3 | 0 | 4 | 0 | 0 | 2 |
| ICE-13_24 | 8 | 0 | 0 | 0 | 0 | 2 | 0 | 0 | 28 | 0 | 0 | 7 | 7 | 16 | 0 | 2 | 12 | 0 | 2 | 4 | 0 | 6 | 0 | 1 | 3 |

Table S2: Counts of OTUs matching eukaryotes, algae (*Chloroplastida*) and bacteria, clustered at 99%, 97% and 95% similarities after removal of singletons.

|  | 99 % | 97 % | 95 % |
| --- | --- | --- | --- |
| All eukaryotes | 4,170 | 2,811 | 1,901 |
| Chloroplastida | 807 | 567 | 395 |
| Bacteria | 1,591 | 1,733 | 1,500 |

Table S3: Distribution of OTUs clustered at 99%, 97% and 95% similarities aligned and assigned to eukaryotes. Values are the relative abundance of the taxa in percentage of total sequences and table shows taxa with >0.1% abundance.

|  | Drangajokull | | | Laugafell | | | Hofsjokull | | | Vatnajokull | | | Langjokull | | | Langjokull | | |
| --- | --- | --- | --- | --- | --- | --- | --- | --- | --- | --- | --- | --- | --- | --- | --- | --- | --- | --- |
|  | **ICE12.2.3** | | | **ICE12.4** | | | **ICE12.6.7** | | | **ICE13.14.15** | | | **ICE13.16.18** | | | **ICE13.19** | | |
| OTU similarity | **99%** | **97%** | **95%** | **99%** | **97%** | **95%** | **99%** | **97%** | **95%** | **99%** | **97%** | **95%** | **99%** | **97%** | **95%** | **99%** | **97%** | **95%** |
| Archaeplastida; Chloroplastida | 32.3 | 45.3 | 46.3 | 32.6 | 35.9 | 30.6 | 63.9 | 60.6 | 56.3 | 59.9 | 56.6 | 57.3 | 2.6 | 9.7 | 10.2 | 1.8 | 4.5 | 5.3 |
| Opisthokonta; Fungi | 29.8 | 27.0 | 23.4 | 54.7 | 53.5 | 46.8 | 17.9 | 19.7 | 18.3 | 39.7 | 41.4 | 41.7 | 97.4 | 89.9 | 89.1 | 98.2 | 94.9 | 94.7 |
| SAR; Alveolata | 0.2 | 0.1 | 0.1 | 11.6 | 6.3 | 5.9 | 1.6 | 2.6 | 2.6 | 0.0 | 0.0 | 0.0 | 0.0 | 0.0 | 0.0 | 0.0 | 0.0 | 0.0 |
| SAR; Rhizaria | 37.7 | 27.6 | 30.2 | 1.2 | 4.2 | 16.7 | 16.4 | 16.5 | 21.7 | 0.0 | 1.5 | 0.4 | 0.0 | 0.2 | 0.6 | 0.0 | 0.7 | 0.0 |
| SAR; Stramenopiles | 0.0 | 0.0 | 0.0 | 0.0 | 0.0 | 0.0 | 0.2 | 0.6 | 1.1 | 0.4 | 0.6 | 0.5 | 0.0 | 0.2 | 0.1 | 0.0 | 0.0 | 0.0 |
| Zeuk77; uncultured eukaryote | 0.0 | 0.0 | 0.0 | 0.0 | 0.0 | 0.0 | 0.0 | 0.0 | 0.0 | 0.0 | 0.0 | 0.0 | 0.0 | 0.0 | 0.0 | 0.0 | 0.0 | 0.0 |

|  | Snaefellsjokull | | | Eyafjallajokull | | | Mýrdalsjokull | | | Eyafjallajokull | | | Eyafjallajokull | | | Eyafjallajokull | | |
| --- | --- | --- | --- | --- | --- | --- | --- | --- | --- | --- | --- | --- | --- | --- | --- | --- | --- | --- |
|  | **ICE13.21.24** | | | **ICE13.4.5.6** | | | **ICE13.8.9** | | | **ICE14.1** | | | **ICE14.2** | | | **ICE14.3** | | |
| OTU similarity | **99%** | **97%** | **95%** | **99%** | **97%** | **95%** | **99%** | **97%** | **95%** | **99%** | **97%** | **95%** | **99%** | **97%** | **95%** | **99%** | **97%** | **95%** |
| Archaeplastida; Chloroplastida | 8.1 | 7.8 | 5.5 | 35.9 | 29.8 | 27.5 | 21.4 | 19.7 | 20.3 | 21.1 | 17.3 | 16.4 | 66.0 | 57.9 | 55.4 | 43.9 | 35.4 | 33.2 |
| Opisthokonta; Fungi | 91.9 | 87.4 | 87.9 | 63.4 | 67.0 | 70.0 | 78.6 | 78.7 | 76.6 | 12.2 | 12.1 | 12.0 | 10.5 | 12.0 | 11.5 | 4.7 | 6.6 | 7.7 |
| SAR; Alveolata | 0.0 | 0.0 | 0.2 | 0.0 | 0.0 | 0.0 | 0.0 | 0.0 | 0.0 | 19.6 | 16.3 | 15.6 | 1.5 | 1.8 | 1.9 | 4.2 | 3.7 | 3.6 |
| SAR; Rhizaria | 0.0 | 4.8 | 6.4 | 0.0 | 2.0 | 0.5 | 0.0 | 1.6 | 3.1 | 0.0 | 0.0 | 0.5 | 0.0 | 0.0 | 1.1 | 0.0 | 0.0 | 0.3 |
| SAR; Stramenopiles | 0.0 | 0.0 | 0.0 | 0.7 | 1.1 | 2.0 | 0.0 | 0.0 | 0.0 | 46.3 | 54.3 | 55.4 | 21.5 | 28.1 | 29.7 | 47.0 | 53.9 | 54.7 |
| Zeuk77; uncultured eukaryote | 0.0 | 0.0 | 0.0 | 0.0 | 0.0 | 0.0 | 0.0 | 0.0 | 0.0 | 0.8 | 0.0 | 0.1 | 0.4 | 0.2 | 0.3 | 0.2 | 0.4 | 0.5 |

Table S4: Distribution of OTUs clustered at 99%, 97% and 95% similarities aligned and assigned to algal sequences (*Chloroplastida*). Values are the relative abundance of the taxa in percentage of total sequences and table shows taxa with >0.05% abundance.

|  | Drangajökull | | | Laugafell | | | Hofsjökull | | | Vatnajökull | | | Langjökull | | | Langjökull | | |
| --- | --- | --- | --- | --- | --- | --- | --- | --- | --- | --- | --- | --- | --- | --- | --- | --- | --- | --- |
|  | **ICE12.2.3** | | | **ICE12.4** | | | **ICE12.6.7** | | | **ICE13.14.15** | | | **ICE13.16.18** | | | **ICE13.19** | | |
| OTU similarity | **99%** | **97%** | **95%** | **99%** | **97%** | **95%** | **99%** | **97%** | **95%** | **99%** | **97%** | **95%** | **99%** | **97%** | **95%** | **99%** | **97%** | **95%** |
| Chlorophyceae; Chloromonas cf. alpina CCCryo 033-99 | 0.0 | 0.2 | 0.5 | 0.0 | 1.7 | 2.6 | 0.5 | 0.8 | 1.0 | 0.0 | 0.0 | 1.5 | 3.4 | 2.2 | 2.7 | 0.0 | 0.0 | 0.0 |
| Chlorophyceae; Chloromonas nivalis CCCryo 005-99 | 3.2 | 14.9 | 1.4 | 0.0 | 6.7 | 6.5 | 0.0 | 0.2 | 0.3 | 0.0 | 0.0 | 0.0 | 0.0 | 0.0 | 0.0 | 0.0 | 0.0 | 2.6 |
| Chlorophyceae; Chloromonas polyptera | 32.8 | 27.8 | 43.3 | 21.1 | 23.3 | 29.9 | 4.5 | 5.1 | 4.8 | 25.0 | 14.3 | 13.6 | 33.3 | 31.1 | 32.7 | 52.4 | 36.1 | 28.2 |
| Chlorophyceae; Chloromonas tughillensis | 2.5 | 4.6 | 5.7 | 0.0 | 1.7 | 1.3 | 0.0 | 0.0 | 0.0 | 0.0 | 0.0 | 0.0 | 0.0 | 0.0 | 0.0 | 0.0 | 0.0 | 0.0 |
| Chlorophyceae; uncultured Chlamydomonadaceae | 8.9 | 1.4 | 0.0 | 7.9 | 3.3 | 0.0 | 0.0 | 0.0 | 0.0 | 6.3 | 0.0 | 0.0 | 0.0 | 0.0 | 0.0 | 4.8 | 0.0 | 0.0 |
| Chlorophyceae; uncultured Chlamydomonadaceae | 33.4 | 34.5 | 32.3 | 36.8 | 43.3 | 37.7 | 0.5 | 0.5 | 0.4 | 0.0 | 2.0 | 1.5 | 0.0 | 0.7 | 0.7 | 0.0 | 5.6 | 5.1 |
| Chlorophyceae; uncultured Chlamydomonadaceae | 0.6 | 0.0 | 0.0 | 0.0 | 0.0 | 0.0 | 0.0 | 0.0 | 0.0 | 0.0 | 0.0 | 0.0 | 0.0 | 0.0 | 0.0 | 0.0 | 0.0 | 0.0 |
| Trebouxiophyceae; Chloroidium | 0.0 | 0.0 | 0.0 | 0.0 | 0.0 | 0.0 | 0.0 | 0.0 | 0.0 | 0.0 | 0.0 | 0.0 | 0.0 | 0.0 | 0.0 | 0.0 | 0.0 | 0.0 |
| Trebouxiophyceae; Coccomyxa | 0.0 | 0.0 | 0.0 | 0.0 | 0.0 | 0.0 | 0.0 | 0.0 | 0.0 | 0.0 | 0.0 | 4.5 | 0.0 | 0.0 | 0.0 | 0.0 | 0.0 | 0.0 |
| Trebouxiophyceae; Prototheca cutis | 0.0 | 0.0 | 0.0 | 0.0 | 0.0 | 1.3 | 0.0 | 0.2 | 0.8 | 0.0 | 0.0 | 0.0 | 62.1 | 65.2 | 63.3 | 42.9 | 58.3 | 64.1 |
| Trebouxiophyceae; Raphidonema nivale CCCryo 130-01 | 0.0 | 0.0 | 0.0 | 0.0 | 0.0 | 0.0 | 0.0 | 0.0 | 0.0 | 0.0 | 0.0 | 0.0 | 0.0 | 0.0 | 0.0 | 0.0 | 0.0 | 0.0 |
| Trebouxiophyceae; Raphidonema pyrenoidifera | 0.0 | 0.0 | 0.0 | 0.0 | 0.0 | 0.0 | 0.0 | 0.0 | 0.0 | 0.0 | 0.0 | 0.0 | 0.0 | 0.0 | 0.0 | 0.0 | 0.0 | 0.0 |
| Trebouxiophyceae; Raphidonema sempervirens | 18.5 | 16.6 | 17.0 | 34.2 | 20.0 | 20.8 | 94.6 | 93.3 | 92.7 | 68.8 | 53.1 | 45.5 | 1.1 | 0.7 | 0.7 | 0.0 | 0.0 | 0.0 |
| Trebouxiophyceae; Trebouxia usneae | 0.0 | 0.0 | 0.0 | 0.0 | 0.0 | 0.0 | 0.0 | 0.0 | 0.0 | 0.0 | 0.0 | 15.2 | 0.0 | 0.0 | 0.0 | 0.0 | 0.0 | 0.0 |
| Trebouxiophyceae; uncultured | 0.0 | 0.0 | 0.0 | 0.0 | 0.0 | 0.0 | 0.0 | 0.0 | 0.0 | 0.0 | 30.6 | 18.2 | 0.0 | 0.0 | 0.0 | 0.0 | 0.0 | 0.0 |

**Table S4 continued.**

|  | Snaefellsjökull | | | Eyafjallajökull | | | Mýrdalsjökull | | | Eyafjallajökull | | | Eyafjallajökull | | | Eyafjallajökull | | |
| --- | --- | --- | --- | --- | --- | --- | --- | --- | --- | --- | --- | --- | --- | --- | --- | --- | --- | --- |
|  | **ICE13.21.24** | | | **ICE13.4.5.6** | | | **ICE13.8.9** | | | **ICE14.1** | | | **ICE14.2** | | | **ICE14.3** | | |
| OTU similarity | **99%** | **97%** | **95%** | **99%** | **97%** | **95%** | **99%** | **97%** | **95%** | **99%** | **97%** | **95%** | **99%** | **97%** | **95%** | **99%** | **97%** | **95%** |
| Chlorophyceae; Chloromonas cf. alpina CCCryo 033-99 | 0.0 | 0.0 | 0.0 | 0.0 | 16.7 | 12.1 | 0.0 | 0.0 | 0.0 | 0.1 | 0.1 | 0.0 | 0.0 | 0.0 | 0.0 | 0.1 | 0.1 | 0.0 |
| Chlorophyceae; Chloromonas nivalis CCCryo 005-99 | 0.0 | 0.0 | 0.0 | 0.0 | 0.0 | 3.0 | 0.0 | 0.0 | 12.5 | 1.2 | 1.0 | 0.0 | 0.0 | 0.0 | 0.0 | 0.0 | 0.0 | 0.0 |
| Chlorophyceae; Chloromonas polyptera | 58.8 | 75.9 | 63.6 | 40.0 | 41.7 | 15.2 | 71.4 | 71.4 | 50.0 | 4.7 | 6.5 | 7.9 | 0.8 | 0.9 | 0.9 | 3.1 | 3.6 | 4.0 |
| Chlorophyceae; Chloromonas tughillensis | 0.0 | 0.0 | 0.0 | 0.0 | 0.0 | 0.0 | 0.0 | 0.0 | 0.0 | 0.0 | 0.0 | 0.0 | 0.0 | 0.0 | 0.0 | 0.0 | 0.0 | 0.0 |
| Chlorophyceae; uncultured Chlamydomonadaceae | 5.9 | 0.0 | 0.0 | 0.0 | 0.0 | 0.0 | 14.3 | 14.3 | 0.0 | 0.3 | 0.0 | 0.0 | 0.0 | 0.0 | 0.0 | 0.0 | 0.0 | 0.0 |
| Chlorophyceae; uncultured Chlamydomonadaceae | 23.5 | 20.7 | 21.2 | 20.0 | 8.3 | 3.0 | 14.3 | 14.3 | 37.5 | 0.2 | 0.8 | 1.1 | 0.0 | 0.1 | 0.1 | 0.0 | 0.0 | 0.2 |
| Chlorophyceae; uncultured Chlamydomonadaceae | 0.0 | 0.0 | 0.0 | 0.0 | 0.0 | 0.0 | 0.0 | 0.0 | 0.0 | 0.3 | 0.1 | 0.0 | 0.0 | 0.0 | 0.0 | 0.2 | 0.2 | 0.0 |
| Trebouxiophyceae; Chloroidium | 0.0 | 0.0 | 6.1 | 0.0 | 0.0 | 21.2 | 0.0 | 0.0 | 0.0 | 0.0 | 0.0 | 0.0 | 0.0 | 0.0 | 0.0 | 0.0 | 0.0 | 0.0 |
| Trebouxiophyceae; Coccomyxa | 0.0 | 0.0 | 0.0 | 0.0 | 0.0 | 27.3 | 0.0 | 0.0 | 0.0 | 0.0 | 0.0 | 0.0 | 0.0 | 0.0 | 0.0 | 0.0 | 0.0 | 0.0 |
| Trebouxiophyceae; Prototheca cutis | 5.9 | 0.0 | 6.1 | 0.0 | 0.0 | 0.0 | 0.0 | 0.0 | 0.0 | 0.0 | 0.1 | 0.1 | 0.0 | 0.1 | 0.2 | 0.1 | 0.2 | 0.4 |
| Trebouxiophyceae; Raphidonema nivale CCCryo 130-01 | 0.0 | 0.0 | 0.0 | 0.0 | 0.0 | 0.0 | 0.0 | 0.0 | 0.0 | 0.0 | 0.5 | 0.0 | 0.0 | 0.4 | 0.0 | 0.0 | 0.5 | 0.0 |
| Trebouxiophyceae; Raphidonema pyrenoidifera | 0.0 | 0.0 | 0.0 | 0.0 | 0.0 | 0.0 | 0.0 | 0.0 | 0.0 | 0.0 | 0.0 | 0.0 | 0.0 | 0.0 | 0.1 | 0.0 | 0.0 | 0.1 |
| Trebouxiophyceae; Raphidonema sempervirens | 5.9 | 3.4 | 3.0 | 40.0 | 33.3 | 12.1 | 0.0 | 0.0 | 0.0 | 93.2 | 90.9 | 90.1 | 99.2 | 98.5 | 97.0 | 96.5 | 95.4 | 92.2 |
| Trebouxiophyceae; Trebouxia usneae | 0.0 | 0.0 | 0.0 | 0.0 | 0.0 | 0.0 | 0.0 | 0.0 | 0.0 | 0.0 | 0.0 | 0.0 | 0.0 | 0.0 | 0.0 | 0.0 | 0.0 | 0.0 |
| Trebouxiophyceae; uncultured | 0.0 | 0.0 | 0.0 | 0.0 | 0.0 | 3.0 | 0.0 | 0.0 | 0.0 | 0.0 | 0.0 | 0.0 | 0.0 | 0.0 | 0.0 | 0.0 | 0.0 | 0.0 |

Table S5: Distribution of OTUs clustered at 99%, 97% and 95% similarities aligned and assigned to bacterial sequences. Values are the relative abundance of the taxa in percentage of total sequences and table shows taxa with >0.01% abundance.

|  | Drangajökull | | | Laugafell | | | Hofsjökull | | | Vatnajökull | | | Langjökull | | | Langjökull | | |
| --- | --- | --- | --- | --- | --- | --- | --- | --- | --- | --- | --- | --- | --- | --- | --- | --- | --- | --- |
|  | **ICE12.2.3** | | | **ICE12.4** | | | **ICE12.6.7** | | | **ICE13.14.15** | | | **ICE13.16.18** | | | **ICE13.19** | | |
| OTU similarity | **99%** | **97%** | **95%** | **99%** | **97%** | **95%** | **99%** | **97%** | **95%** | **99%** | **97%** | **95%** | **99%** | **97%** | **95%** | **99%** | **97%** | **95%** |
| Acidobacteria; Acidobacteriia | 0.6 | 0.4 | 0.3 | 0.0 | 0.0 | 0.0 | 0.0 | 0.1 | 0.2 | 0.0 | 0.0 | 0.2 | 0.0 | 0.2 | 0.0 | 0.0 | 0.0 | 0.0 |
| Actinobacteria; Actinobacteria | 10.7 | 6.0 | 5.6 | 0.8 | 2.6 | 2.8 | 1.2 | 0.9 | 1.4 | 0.0 | 0.3 | 1.4 | 3.6 | 4.1 | 3.4 | 26.4 | 15.9 | 14.6 |
| Bacteroidetes; Cytophagia | 0.2 | 0.1 | 0.0 | 10.0 | 5.4 | 4.8 | 1.2 | 1.4 | 1.1 | 0.0 | 2.3 | 1.8 | 0.0 | 0.0 | 0.4 | 0.0 | 0.7 | 0.6 |
| Bacteroidetes; Flavobacteriia | 0.0 | 0.0 | 0.1 | 0.0 | 0.0 | 0.0 | 0.0 | 0.0 | 0.0 | 0.0 | 0.0 | 0.0 | 0.0 | 0.0 | 0.0 | 0.0 | 0.0 | 0.0 |
| Bacteroidetes; Sphingobacteriia | 23.6 | 18.8 | 18.3 | 0.0 | 0.0 | 0.0 | 50.1 | 32.2 | 29.3 | 0.0 | 0.0 | 0.0 | 0.9 | 0.2 | 0.2 | 0.5 | 0.7 | 0.9 |
| Bacteroidetes; Saprospirae | 8.0 | 24.1 | 26.6 | 49.2 | 28.5 | 26.8 | 29.5 | 38.4 | 37.1 | 0.0 | 0.0 | 0.0 | 1.8 | 1.4 | 1.3 | 2.0 | 3.8 | 4.8 |
| Chlorobi; Ignavibacteria | 8.0 | 0.0 | 0.0 | 0.0 | 0.0 | 0.0 | 0.0 | 0.0 | 0.0 | 0.0 | 0.0 | 0.0 | 0.0 | 0.0 | 0.0 | 0.0 | 0.0 | 0.0 |
| Cyanobacteria; | 0.0 | 0.0 | 0.0 | 0.0 | 0.0 | 0.0 | 0.0 | 0.0 | 0.0 | 0.0 | 2.7 | 11.7 | 0.0 | 0.0 | 0.5 | 0.0 | 0.0 | 0.3 |
| Cyanobacteria; Nostocophycideae | 0.0 | 0.0 | 0.0 | 0.0 | 0.2 | 0.2 | 0.0 | 0.0 | 0.0 | 57.1 | 30.8 | 14.6 | 0.0 | 0.0 | 0.0 | 0.0 | 0.0 | 0.0 |
| Cyanobacteria; Oscillatoriophycideae | 0.0 | 0.0 | 0.0 | 0.0 | 0.0 | 0.2 | 0.0 | 0.0 | 0.0 | 21.4 | 9.0 | 16.2 | 0.9 | 0.5 | 0.5 | 0.0 | 0.0 | 0.0 |
| Cyanobacteria; Synechococcophycideae | 0.0 | 0.5 | 0.4 | 0.8 | 0.5 | 0.6 | 0.0 | 0.0 | 0.0 | 0.0 | 3.7 | 5.9 | 0.0 | 0.0 | 0.2 | 0.0 | 0.0 | 0.2 |
| Firmicutes; Bacilli | 0.0 | 0.0 | 0.0 | 0.0 | 0.0 | 0.0 | 0.0 | 0.0 | 0.0 | 0.0 | 0.0 | 0.0 | 0.0 | 0.0 | 0.0 | 0.0 | 0.1 | 0.1 |
| Gemmatimonadetes; Gemmatimonadetes | 0.2 | 0.1 | 0.1 | 0.0 | 0.5 | 0.7 | 0.0 | 0.0 | 0.0 | 0.0 | 0.0 | 0.0 | 0.0 | 0.0 | 0.0 | 0.0 | 0.1 | 0.1 |
| Proteobacteria; Alphaproteobacteria | 46.4 | 42.0 | 41.4 | 0.8 | 5.4 | 8.1 | 6.2 | 6.6 | 7.5 | 21.4 | 49.5 | 44.6 | 3.6 | 11.6 | 12.1 | 3.4 | 5.1 | 6.0 |
| Proteobacteria; Betaproteobacteria | 1.6 | 4.9 | 4.1 | 38.3 | 56.2 | 55.1 | 11.5 | 20.1 | 22.9 | 0.0 | 0.3 | 0.9 | 86.5 | 80.3 | 79.3 | 66.0 | 71.9 | 71.1 |
| Proteobacteria; Gammaproteobacteria | 0.6 | 1.3 | 1.1 | 0.0 | 0.0 | 0.2 | 0.3 | 0.1 | 0.3 | 0.0 | 0.0 | 1.4 | 2.7 | 1.8 | 2.2 | 1.8 | 1.6 | 1.4 |
| Bacteria; WPS-2 | 0.2 | 0.9 | 0.9 | 0.0 | 0.0 | 0.0 | 0.0 | 0.0 | 0.0 | 0.0 | 0.3 | 0.2 | 0.0 | 0.0 | 0.0 | 0.0 | 0.0 | 0.0 |

**Table S5 continued.**

|  | Snaefellsjökull | | | Eyafjallajökull | | | Mýrdalsjökull | | | Eyafjallajökull | | | Eyafjallajökull | | | Eyafjallajökull | | |
| --- | --- | --- | --- | --- | --- | --- | --- | --- | --- | --- | --- | --- | --- | --- | --- | --- | --- | --- |
|  | **ICE13.21.24** | | | **ICE13.4.5.6** | | | **ICE13.8.9** | | | **ICE14.1** | | | **ICE14.2** | | | **ICE14.3** | | |
| OTU similarity | **99%** | **97%** | **95%** | **99%** | **97%** | **95%** | **99%** | **97%** | **95%** | **99%** | **97%** | **95%** | **99%** | **97%** | **95%** | **99%** | **97%** | **95%** |
| Acidobacteria; Acidobacteriia | 0.0 | 0.5 | 0.5 | 5.5 | 8.6 | 2.4 | 0.0 | 0.0 | 0.0 | 0.0 | 0.0 | 0.0 | 0.0 | 0.0 | 0.0 | 0.0 | 0.0 | 0.0 |
| Actinobacteria; Actinobacteria | 0.0 | 0.0 | 0.1 | 3.6 | 1.0 | 0.4 | 0.0 | 8.0 | 4.1 | 0.5 | 0.8 | 0.9 | 0.4 | 0.5 | 0.6 | 0.2 | 0.6 | 0.6 |
| Bacteroidetes; Cytophagia | 0.0 | 0.1 | 0.7 | 0.0 | 5.4 | 2.1 | 0.0 | 12.0 | 8.2 | 0.2 | 0.1 | 0.1 | 0.5 | 0.7 | 0.7 | 2.0 | 1.7 | 1.7 |
| Bacteroidetes; Flavobacteriia | 0.0 | 0.0 | 0.0 | 0.0 | 0.0 | 0.0 | 50.0 | 0.0 | 2.0 | 0.0 | 0.0 | 0.0 | 0.0 | 0.0 | 0.0 | 0.0 | 0.0 | 0.0 |
| Bacteroidetes; Sphingobacteriia | 0.0 | 0.0 | 0.0 | 1.8 | 0.6 | 0.5 | 50.0 | 4.0 | 4.1 | 7.1 | 5.3 | 5.1 | 1.1 | 0.8 | 0.8 | 10.5 | 8.1 | 7.7 |
| Bacteroidetes; Saprospirae | 0.0 | 0.0 | 0.1 | 0.0 | 0.2 | 0.1 | 0.0 | 4.0 | 6.1 | 56.0 | 45.5 | 43.1 | 31.2 | 28.0 | 27.2 | 33.5 | 29.7 | 28.6 |
| Chlorobi; Ignavibacteria | 0.0 | 0.0 | 0.0 | 0.0 | 0.0 | 0.0 | 0.0 | 12.0 | 6.1 | 0.0 | 0.0 | 0.0 | 0.0 | 0.0 | 0.0 | 0.0 | 0.0 | 0.0 |
| Cyanobacteria; | 0.0 | 0.0 | 0.1 | 0.0 | 4.1 | 56.6 | 0.0 | 0.0 | 18.4 | 0.0 | 0.0 | 0.2 | 0.0 | 0.0 | 0.2 | 0.0 | 0.0 | 0.1 |
| Cyanobacteria; Nostocophycideae | 1.0 | 1.5 | 0.1 | 14.5 | 13.7 | 2.8 | 0.0 | 0.0 | 0.0 | 0.0 | 0.0 | 0.0 | 0.0 | 0.0 | 0.0 | 0.0 | 0.0 | 0.0 |
| Cyanobacteria; Oscillatoriophycideae | 0.0 | 0.0 | 1.0 | 23.6 | 4.3 | 3.8 | 0.0 | 0.0 | 0.0 | 0.0 | 0.0 | 0.0 | 0.0 | 0.0 | 0.0 | 0.0 | 0.0 | 0.0 |
| Cyanobacteria; Synechococcophycideae | 0.0 | 0.0 | 0.5 | 0.0 | 0.3 | 1.2 | 0.0 | 0.0 | 0.0 | 0.0 | 0.0 | 0.0 | 0.0 | 0.0 | 0.0 | 0.0 | 0.0 | 0.0 |
| Firmicutes; Bacilli | 0.0 | 0.0 | 0.0 | 0.0 | 0.0 | 0.0 | 0.0 | 8.0 | 6.1 | 0.0 | 0.0 | 0.0 | 0.0 | 0.0 | 0.0 | 0.0 | 0.0 | 0.0 |
| Gemmatimonadetes; Gemmatimonadetes | 0.0 | 0.0 | 0.0 | 0.0 | 0.0 | 0.0 | 0.0 | 0.0 | 0.0 | 0.0 | 0.0 | 0.0 | 0.0 | 0.0 | 0.0 | 0.0 | 0.0 | 0.0 |
| Proteobacteria; Alphaproteobacteria | 0.0 | 2.7 | 2.9 | 10.9 | 42.6 | 17.9 | 0.0 | 20.0 | 22.4 | 14.0 | 15.6 | 15.6 | 3.6 | 4.5 | 4.9 | 30.3 | 31.2 | 31.4 |
| Proteobacteria; Betaproteobacteria | 99.0 | 95.1 | 92.8 | 40.0 | 16.2 | 8.1 | 0.0 | 24.0 | 12.2 | 22.2 | 32.6 | 34.6 | 63.0 | 65.4 | 65.5 | 23.4 | 28.7 | 29.7 |
| Proteobacteria; Gammaproteobacteria | 0.0 | 0.1 | 0.9 | 0.0 | 0.3 | 2.7 | 0.0 | 0.0 | 6.1 | 0.0 | 0.0 | 0.0 | 0.1 | 0.1 | 0.1 | 0.1 | 0.1 | 0.1 |
| Bacteria; WPS-2 | 0.0 | 0.1 | 0.1 | 0.0 | 2.1 | 0.8 | 0.0 | 0.0 | 0.0 | 0.0 | 0.0 | 0.0 | 0.0 | 0.0 | 0.0 | 0.0 | 0.0 | 0.0 |

Table S6: Distribution of OTUs clustered at 99%, 97% and 95% similarities aligned and assigned to archaeal sequences. Values are the relative abundance of the taxa in percentage of total sequences.

|  | Laugafell | | | Hofsjökull | | | Vatnajökull | | | Langjökull | | | Langjökull | | | Snaefellsjökull | | |
| --- | --- | --- | --- | --- | --- | --- | --- | --- | --- | --- | --- | --- | --- | --- | --- | --- | --- | --- |
|  | **ICE12.4** | | | **ICE12.6.7** | | | **ICE13.14.15** | | | **ICE13.16.18** | | | **ICE13.16.19** | | | **ICE13.21.24** | | |
| OTU similarity | **99%** | **97%** | **95%** | **99%** | **97%** | **95%** | **99%** | **97%** | **95%** | **99%** | **97%** | **95%** | **99%** | **97%** | **95%** | **99%** | **97%** | **95%** |
| Crenarchaeota; MBGA | 0.2 | 0.0 | 1.7 | 0.0 | 0.0 | 0.0 | 0.0 | 0.0 | 0.0 | 0.0 | 0.0 | 2.1 | 1.3 | 0.0 | 0.1 | 0.0 | 0.2 | 0.4 |
| Crenarchaeota; Thaumarchaeota; Cenarchaeales; Cenarchaeaceae | 0.6 | 2.8 | 3.3 | 0.0 | 0.7 | 1.8 | 1.4 | 7.7 | 8.6 | 0.3 | 1.1 | 1.5 | 0.0 | 2.4 | 3.3 | 0.6 | 4.2 | 6.0 |
| Crenarchaeota; Thaumarchaeota; Cenarchaeales; SAGMA-X | 0.0 | 0.0 | 0.1 | 0.0 | 0.0 | 0.0 | 0.0 | 0.0 | 0.0 | 0.0 | 0.5 | 0.0 | 0.0 | 0.0 | 1.6 | 0.0 | 0.0 | 0.0 |
| Crenarchaeota; Thaumarchaeota; Nitrososphaerales; Nitrososphaeraceae | 99.3 | 96.5 | 91.7 | 33.3 | 27.6 | 29.4 | 98.6 | 92.0 | 91.3 | 99.7 | 98.1 | 92.3 | 98.7 | 97.6 | 94.2 | 99.4 | 95.6 | 93.1 |
| Euryarchaeota; Methanobacteria; Methanobacteriales; MSBL1 | 0.0 | 0.0 | 0.0 | 0.0 | 0.0 | 0.0 | 0.0 | 0.0 | 0.0 | 0.0 | 0.0 | 0.0 | 0.0 | 0.0 | 0.0 | 0.0 | 0.0 | 0.0 |
| Euryarchaeota; Methanomicrobia; Methanosarcinales; Methanosarcinaceae | 0.0 | 0.0 | 0.0 | 66.7 | 71.6 | 68.9 | 0.0 | 0.0 | 0.0 | 0.0 | 0.0 | 0.0 | 0.0 | 0.0 | 0.0 | 0.0 | 0.0 | 0.0 |

|  | Eyafjallajökull | | | Eyafjallajökull | | | Eyafjallajökull | | |
| --- | --- | --- | --- | --- | --- | --- | --- | --- | --- |
|  | **ICE14.1** | | | **ICE14.2** | | | **ICE14.3** | | |
| OTU similarity | **99%** | **97%** | **95%** | **99%** | **97%** | **95%** | **99%** | **97%** | **95%** |
| Crenarchaeota; MBGA | 0.0 | 0.0 | 0.0 | 0.0 | 0.0 | 0.0 | 0.0 | 0.0 | 0.0 |
| Crenarchaeota; Thaumarchaeota; Cenarchaeales; Cenarchaeaceae | 0.0 | 0.0 | 0.9 | 0.0 | 0.0 | 2.1 | 0.0 | 0.0 | 4.9 |
| Crenarchaeota; Thaumarchaeota; Cenarchaeales; SAGMA-X | 0.0 | 0.0 | 0.0 | 15.4 | 18.1 | 17.5 | 0.0 | 0.0 | 0.0 |
| Crenarchaeota; Thaumarchaeota; Nitrososphaerales; Nitrososphaeraceae | 99.9 | 100.0 | 99.0 | 72.3 | 78.6 | 77.2 | 99.9 | 99.9 | 95.8 |
| Euryarchaeota; Methanobacteria; Methanobacteriales; MSBL1 | 0.0 | 0.0 | 0.0 | 0.0 | 0.0 | 0.0 | 0.0 | 0.0 | 0.0 |
| Euryarchaeota; Methanomicrobia; Methanosarcinales; Methanosarcinaceae | 0.0 | 0.0 | 0.0 | 0.0 | 3.2 | 3.2 | 0.0 | 0.0 | 0.0 |

Table S7: Output of the PHREEQC geochemical modelling shown for one representative sample (ICE-13_15, Vatnajökull).

| Phase | SI | 1=SUPERSATURATED;  0=UNDERSATURATED | log IAP | log KT | Formula |
| --- | --- | --- | --- | --- | --- |
| Albite | -9.3 | 0 | -5.97 | 3.33 | NaAlSi3O8 |
| Albite_high | -10.79 | 0 | -5.97 | 4.82 | NaAlSi3O8 |
| Albite_low | -9.3 | 0 | -5.97 | 3.33 | NaAlSi3O8 |
| Analcime | -8.28 | 0 | -1.1 | 7.18 | Na.96Al.96Si2.04O6:H2O |
| Anorthite | -16.31 | 0 | 14.92 | 31.23 | CaAl2(SiO4)2 |
| Beidellite-Mg | -3.6 | 0 | 4.26 | 7.87 | Mg.165Al2.33Si3.67O10(OH)2 |
| Beidellite-Na | -4.34 | 0 | 3.47 | 7.81 | Na.33Al2.33Si3.67O10(OH)2 |
| Boehmite | 0.74 | 1 | 10.1 | 9.37 | AlO2H |
| Brucite | -12.36 | 0 | 5.73 | 18.09 | Mg(OH)2 |
| Ca-Al_Pyroxene | -21.23 | 0 | 20.43 | 41.66 | CaAl2SiO6 |
| Chalcedony | -1.19 | 0 | -5.51 | -4.32 | SiO2 |
| Clinochlore-14A | -44.5 | 0 | 32.32 | 76.82 | Mg5Al2Si3O10(OH)8 |
| Clinochlore-7A | -48.09 | 0 | 32.32 | 80.41 | Mg5Al2Si3O10(OH)8 |
| Cristobalite(alpha) | -1.51 | 0 | -5.51 | -4 | SiO2 |
| Cristobalite(beta) | -2.03 | 0 | -5.51 | -3.49 | SiO2 |
| Diaspore | 1.18 | 1 | 10.1 | 8.92 | AlHO2 |
| Diopside | -22.5 | 0 | 0.44 | 22.93 | CaMgSi2O6 |
| Enstatite | -12.35 | 0 | 0.22 | 12.57 | MgSiO3 |
| Fayalite | -17.01 | 0 | 4.45 | 21.46 | Fe2SiO4 |
| Fe(OH)2 | -10.45 | 0 | 4.98 | 15.44 | Fe(OH)2 |
| Fe(OH)3 | -5.59 | 0 | 1.41 | 7 | Fe(OH)3 |
| FeO | -10.25 | 0 | 4.98 | 15.23 | FeO |
| Forsterite | -25.13 | 0 | 5.95 | 31.08 | Mg2SiO4 |
| Gibbsite | 0.73 | 1 | 10.1 | 9.38 | Al(OH)3 |
| Goethite | -0.12 | 0 | 1.41 | 1.53 | FeOOH |
| Hedenbergite | -21.74 | 0 | -0.31 | 21.43 | CaFe(SiO3)2 |
| Hematite | 0.67 | 1 | 2.82 | 2.15 | Fe2O3 |
| Hercynite | -9.17 | 0 | 25.19 | 34.36 | FeAl2O4 |
| Kaolinite | 0.13 | 1 | 9.19 | 9.06 | Al2Si2O5(OH)4 |
| Laumontite | -12.38 | 0 | 3.9 | 16.27 | CaAl2Si4O12:4H2O |
| Lawsonite | -11.04 | 0 | 14.92 | 25.96 | CaAl2Si2O7(OH)2:H2O |
| Magnetite | -6.1 | 0 | 7.8 | 13.9 | Fe3O4 |
| Monticellite | -26.69 | 0 | 5.95 | 32.64 | CaMgSiO4 |
| Montmor-Ca | -6.08 | 0 | -2.33 | 3.74 | Ca.165Mg.33Al1.67Si4O10(OH)2 |
| Montmor-Mg | -6.01 | 0 | -2.34 | 3.67 | Mg.495Al1.67Si4O10(OH)2 |
| Montmor-Na | -6.75 | 0 | -3.13 | 3.62 | Na.33Mg.33Al1.67Si4O10(OH)2 |
| Mordenite | -10.66 | 0 | -16.56 | -5.9 | Ca.2895Na.361Al.94Si5.06O12:3.468H2O |
| Mordenite-dehy | -27.42 | 0 | -16.56 | 10.86 | Ca.2895Na.361Al.94Si5.06O12 |
| Nontronite-Ca | -1.81 | 0 | -13.13 | -11.31 | Ca.165Fe2Al.33Si3.67H2O12 |
| Nontronite-H | -1.68 | 0 | -14.07 | -12.39 | H.33Fe2Al.33Si3.67H2O12 |
| Nontronite-Mg | -1.82 | 0 | -13.13 | -11.3 | Mg.165Fe2Al.33Si3.67H2O12 |
| Nontronite-Na | -2.56 | 0 | -13.92 | -11.36 | Na.33Fe2Al.33Si3.67H2O12 |
| Periclase | -18.01 | 0 | 5.73 | 23.74 | MgO |
| Pyrophyllite | -3.54 | 0 | -1.84 | 1.71 | Al2Si4O10(OH)2 |
| Quartz | -0.9 | 0 | -5.51 | -4.62 | SiO2 |
| SiO2(am) | -2.4 | 0 | -5.51 | -3.11 | SiO2 |


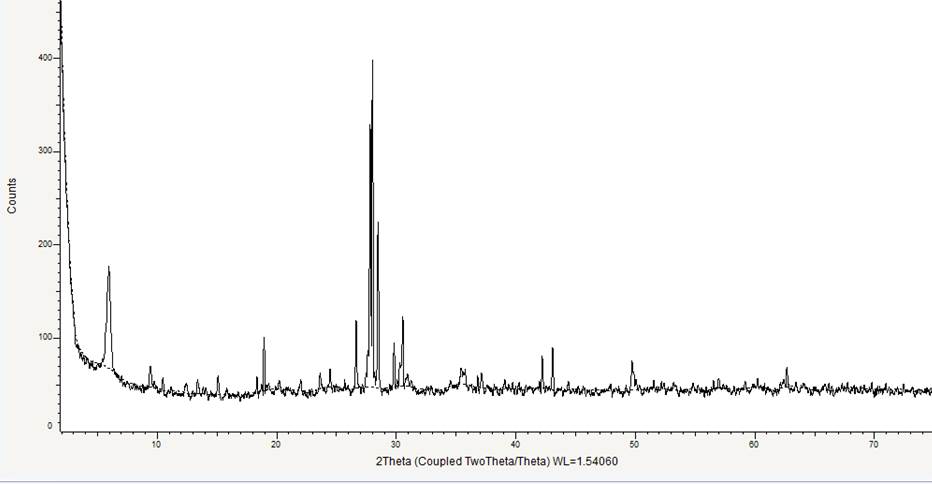


Figure S1: XRD pattern of one representative sample (ICE-13_15, Vatnajökull) and typical for all Iceland samples showing the main mineral components quartz, feldspars, pyroxene and olivine and minor contributions from clays, basaltic glass and hematite.


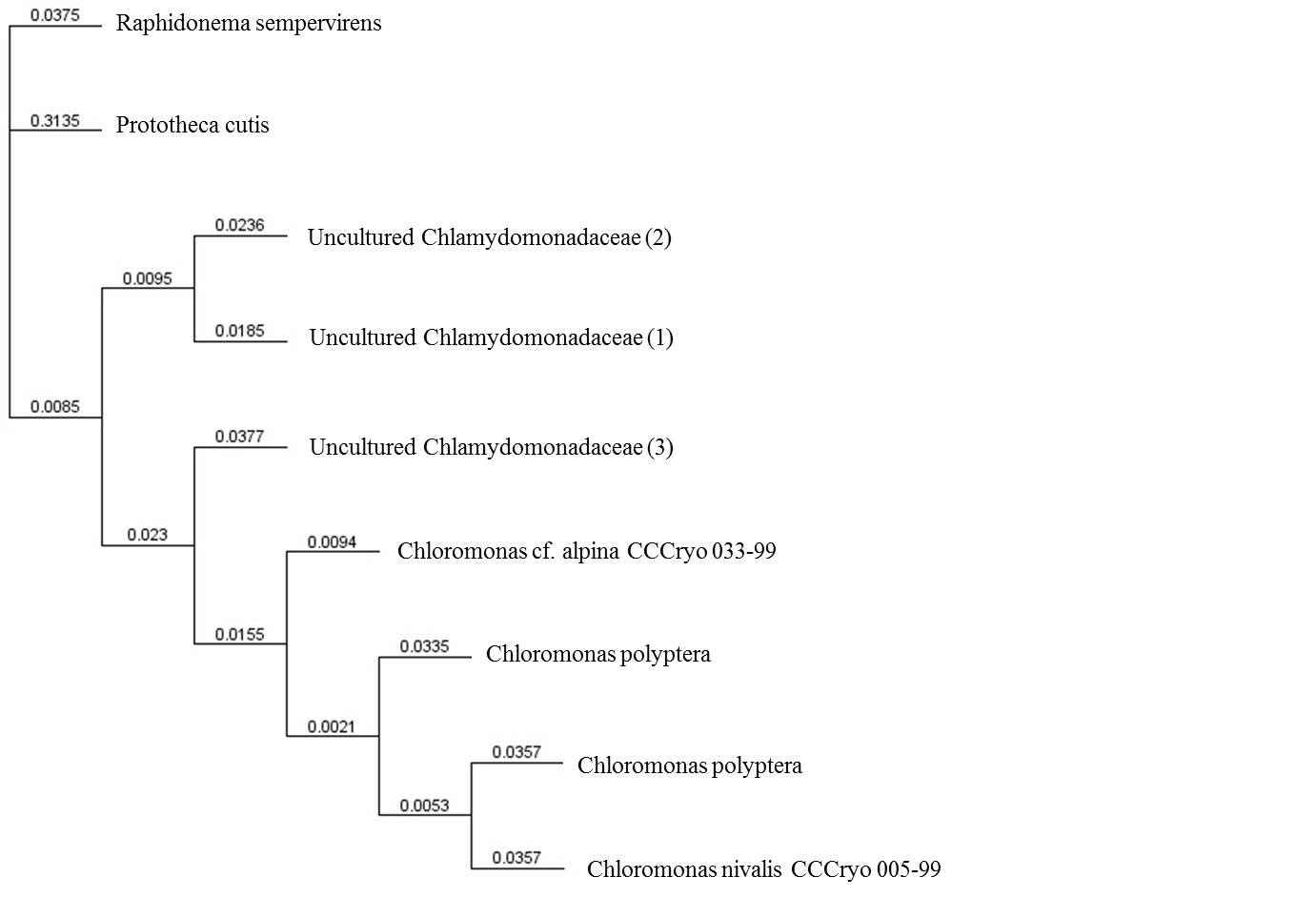


Figure S2: Phylogenetic tree of main algal species showing the inferred evolutionary relationships between the main algal species in our samples. Based on their 18S rRNA sequences they are closely related (89-92% similarity) to other *Chloromonas* species found.
