# Supplementary material for: Microbial diversity on Icelandic glaciers and ice caps
Source: Front Microbiol. 2015 Apr 20;6:307. doi: 10.3389/fmicb.2015.00307 (PMC4403510; doi:10.3389/fmicb.2015.00307)
Supplement: Supplementary file 2 [file DataSheet2.PDF]

Taxonomy Summary. Current Level:

View Figure (.pdf) View Legend (.pdf)

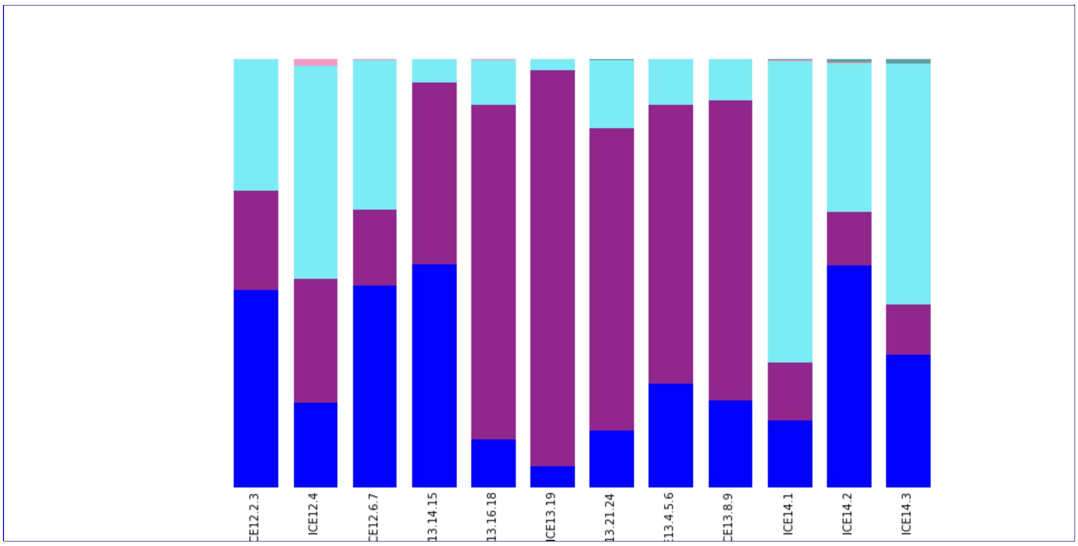

View Table (.txt)

		Total		ICE12.2.3	ICE12.4	ICE12.6.7	ICE13.14.15	ICE13.16.18	ICE13.19	ICE13.21.24	ICE13.4.5.6	ICE13.8.9	ICE14.1	ICE14.2	ICE14.3
Legend	Taxonomy	count	%	%	%	%	%	%	%	%	%	%	%	%	%
	Eukaryota;__Amoebozoa	0	0.0%	0.0%	0.0%	0.0%	0.0%	0.0%	0.0%	0.0%	0.0%	0.0%	0.0%	0.0%	0.0%
	Eukaryota;__Archaeplastida	3	28.1%	46.0%	19.8%	47.2%	52.0%	11.1%	5.0%	13.4%	24.1%	20.2%	15.7%	51.9%	31.0%
	Eukaryota;__Centrohelida	0	0.0%	0.0%	0.0%	0.0%	0.0%	0.0%	0.0%	0.0%	0.0%	0.0%	0.0%	0.0%	0.0%
	Eukaryota;__Kathablepharidae	0	0.0%	0.0%	0.0%	0.0%	0.0%	0.0%	0.0%	0.0%	0.0%	0.0%	0.0%	0.0%	0.0%
	Eukaryota;__Opisthokonta	5	43.8%	23.2%	29.0%	17.7%	42.4%	78.1%	92.5%	70.5%	65.1%	70.2%	13.3%	12.4%	11.6%
	Eukaryota;__RT5iin25	0	0.0%	0.0%	0.0%	0.0%	0.0%	0.0%	0.0%	0.0%	0.0%	0.0%	0.0%	0.0%	0.0%
	Eukaryota;__SAR	3	27.6%	30.7%	49.6%	34.9%	5.6%	10.5%	2.5%	15.8%	10.7%	9.6%	70.3%	34.6%	56.3%
	Eukaryota;__Zeuk77	0	0.2%	0.1%	1.6%	0.2%	0.0%	0.1%	0.0%	0.0%	0.0%	0.0%	0.5%	0.2%	0.1%
	No blast hit;Other	0	0.2%	0.0%	0.0%	0.0%	0.0%	0.1%	0.0%	0.3%	0.0%	0.0%	0.2%	0.9%	1.0%

Taxonomy Summary. Current Level:

View Figure (.pdf) View Legend (.pdf)

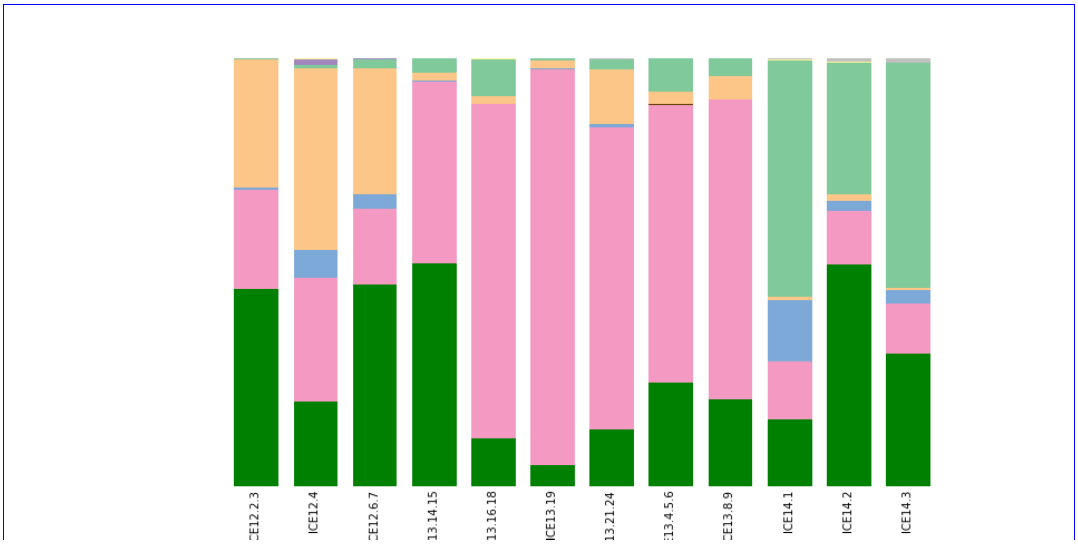

View Table (.txt)

		Total		ICE12.2.3	ICE12.4	ICE12.6.7	ICE13.14.15	ICE13.16.18	ICE13.19	ICE13.21.24	ICE13.4.5.6	ICE13.8.9	ICE14.1	ICE14.2	ICE14.3
Legend	Taxonomy	count	%	%	%	%	%	%	%	%	%	%	%	%	%
	Eukaryota;__Amoebozoa;__Conosa	0	0.0%	0.0%	0.0%	0.0%	0.0%	0.0%	0.0%	0.0%	0.0%	0.0%	0.0%	0.0%	0.0%
	Eukaryota;__Amoebozoa;__Discosea	0	0.0%	0.0%	0.0%	0.0%	0.0%	0.0%	0.0%	0.0%	0.0%	0.0%	0.0%	0.0%	0.0%
	Eukaryota;__Amoebozoa;__Lobosa	0	0.0%	0.0%	0.0%	0.0%	0.0%	0.0%	0.0%	0.0%	0.0%	0.0%	0.0%	0.0%	0.0%
	Eukaryota;__Archaeplastida;__Chloroplastida	3	28.1%	46.0%	19.8%	47.2%	52.0%	11.1%	5.0%	13.4%	24.1%	20.2%	15.7%	51.9%	31.0%
	Eukaryota;__Archaeplastida;__Rhodophyceae	0	0.0%	0.0%	0.0%	0.0%	0.0%	0.0%	0.0%	0.0%	0.0%	0.0%	0.0%	0.0%	0.0%
	Eukaryota;__Centrohelida;__Heterophryidae	0	0.0%	0.0%	0.0%	0.0%	0.0%	0.0%	0.0%	0.0%	0.0%	0.0%	0.0%	0.0%	0.0%
	Eukaryota;__Kathablepharidae;__Roombia	0	0.0%	0.0%	0.0%	0.0%	0.0%	0.0%	0.0%	0.0%	0.0%	0.0%	0.0%	0.0%	0.0%
	Eukaryota;__Opisthokonta;__Fungi	5	43.8%	23.2%	29.0%	17.7%	42.4%	78.1%	92.5%	70.5%	65.1%	70.2%	13.3%	12.4%	11.6%
	Eukaryota;__Opisthokonta;__Holozoa	0	0.0%	0.0%	0.0%	0.0%	0.0%	0.0%	0.0%	0.0%	0.0%	0.0%	0.0%	0.0%	0.0%
	Eukaryota;__Opisthokonta;__Metazoa	0	0.0%	0.0%	0.0%	0.0%	0.0%	0.0%	0.0%	0.0%	0.1%	0.0%	0.0%	0.0%	0.0%
	Eukaryota;__Opisthokonta;__uncultured	0	0.0%	0.0%	0.0%	0.0%	0.0%	0.0%	0.0%	0.0%	0.0%	0.0%	0.0%	0.0%	0.0%
	Eukaryota;__RT5iin25;__uncultured_Eimeriidae	0	0.0%	0.0%	0.0%	0.0%	0.0%	0.0%	0.0%	0.0%	0.0%	0.0%	0.0%	0.0%	0.0%
	Eukaryota;__RT5iin25;__uncultured_freshwater_eukaryote	0	0.0%	0.0%	0.0%	0.0%	0.0%	0.0%	0.0%	0.0%	0.0%	0.0%	0.0%	0.0%	0.0%
	Eukaryota;__SAR;__Alveolata	0	2.7%	0.5%	6.3%	3.4%	0.3%	0.1%	0.1%	0.8%	0.2%	0.0%	14.4%	2.4%	3.3%
	Eukaryota;__SAR;__Rhizaria	1	10.9%	30.1%	42.5%	29.2%	1.9%	1.7%	1.9%	12.7%	2.7%	5.3%	0.9%	1.4%	0.5%
	Eukaryota;__SAR;__Stramenopiles	2	14.0%	0.1%	0.8%	2.3%	3.4%	8.8%	0.5%	2.3%	7.9%	4.3%	55.0%	30.7%	52.4%
	Eukaryota;__Zeuk77;__uncultured_Oxytrichidae	0	0.1%	0.1%	1.4%	0.2%	0.0%	0.0%	0.0%	0.0%	0.0%	0.0%	0.0%	0.0%	0.0%
	Eukaryota;__Zeuk77;__uncultured_eukaryote	0	0.1%	0.0%	0.2%	0.0%	0.0%	0.1%	0.0%	0.0%	0.0%	0.0%	0.4%	0.2%	0.1%
	No blast hit;Other;Other	0	0.2%	0.0%	0.0%	0.0%	0.0%	0.1%	0.0%	0.3%	0.0%	0.0%	0.2%	0.9%	1.0%

Taxonomy Summary. Current Level:

View Figure (.pdf) View Legend (.pdf)

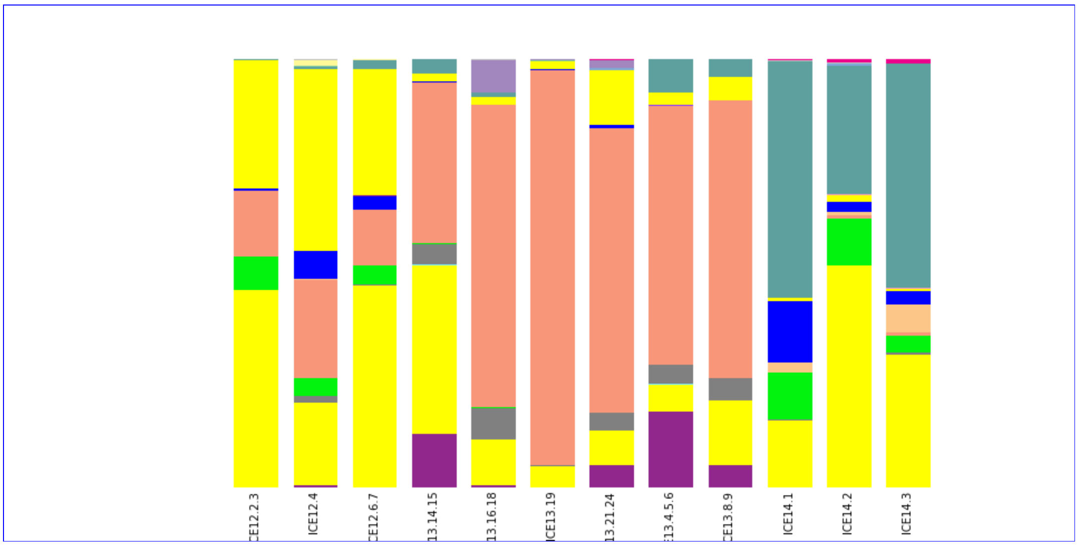

View Table (.txt)

		Total		ICE12.2.3	ICE12.4	ICE12.6.7	ICE13.14.15	ICE13.16.18	ICE13.19	ICE13.21.24	ICE13.4.5.6	ICE13.8.9	ICE14.1	ICE14.2	ICE14.3
Legend	Taxonomy	count	%	%	%	%	%	%	%	%	%	%	%	%	%
	Eukaryota;__Amoebozoa;__Conosa;__Schizoplasmodiida	0	0.0%	0.0%	0.0%	0.0%	0.0%	0.0%	0.0%	0.0%	0.0%	0.0%	0.0%	0.0%	0.0%
	Eukaryota;__Amoebozoa;__Conosa;__Soliformovum	0	0.0%	0.0%	0.0%	0.0%	0.0%	0.0%	0.0%	0.0%	0.0%	0.0%	0.0%	0.0%	0.0%
	Eukaryota;__Amoebozoa;__Discosea;__Flabellinia	0	0.0%	0.0%	0.0%	0.0%	0.0%	0.0%	0.0%	0.0%	0.0%	0.0%	0.0%	0.0%	0.0%
	Eukaryota;__Amoebozoa;__Lobosa;__Tubulinea	0	0.0%	0.0%	0.0%	0.0%	0.0%	0.0%	0.0%	0.0%	0.0%	0.0%	0.0%	0.0%	0.0%
	Eukaryota;__Archaeplastida;__Chloroplastida;__Charophyta	0	3.5%	0.0%	0.6%	0.0%	12.4%	0.5%	0.0%	5.2%	17.6%	5.3%	0.0%	0.0%	0.0%
	Eukaryota;__Archaeplastida;__Chloroplastida;__Chlorophyta	3	24.6%	46.0%	19.2%	47.2%	39.5%	10.6%	5.0%	8.2%	6.4%	14.9%	15.7%	51.8%	30.9%
	Eukaryota;__Archaeplastida;__Chloroplastida;__uncultured	0	0.0%	0.0%	0.0%	0.0%	0.1%	0.0%	0.0%	0.0%	0.1%	0.0%	0.0%	0.0%	0.0%
	Eukaryota;__Archaeplastida;__Rhodophyceae;__Compsopogonales	0	0.0%	0.0%	0.0%	0.0%	0.0%	0.0%	0.0%	0.0%	0.0%	0.0%	0.0%	0.0%	0.0%
	Eukaryota;__Centrohelida;__Heterophryidae;__Heterophrys	0	0.0%	0.0%	0.0%	0.0%	0.0%	0.0%	0.0%	0.0%	0.0%	0.0%	0.0%	0.0%	0.0%
	Eukaryota;__Kathablepharidae;__Roombia;__Roombia_truncata	0	0.0%	0.0%	0.0%	0.0%	0.0%	0.0%	0.0%	0.0%	0.0%	0.0%	0.0%	0.0%	0.0%
	Eukaryota;__Opisthokonta;__Fungi;__Ascomycota	0	2.4%	0.0%	1.4%	0.1%	4.9%	7.2%	0.2%	4.0%	4.5%	5.3%	0.2%	0.0%	0.5%
	Eukaryota;__Opisthokonta;__Fungi;__Basal_fungi	0	3.6%	7.9%	4.4%	4.5%	0.1%	0.4%	0.0%	0.0%	0.0%	0.0%	10.9%	10.9%	3.9%
	Eukaryota;__Opisthokonta;__Fungi;__Basidiomycota	4	37.1%	15.3%	22.8%	13.1%	37.5%	70.5%	92.2%	66.6%	60.5%	64.9%	0.1%	0.7%	0.8%
	Eukaryota;__Opisthokonta;__Fungi;__LKM11	0	0.0%	0.0%	0.0%	0.0%	0.0%	0.0%	0.1%	0.0%	0.0%	0.0%	0.0%	0.0%	0.0%
	Eukaryota;__Opisthokonta;__Fungi;__LKM15	0	0.8%	0.0%	0.4%	0.0%	0.0%	0.0%	0.0%	0.0%	0.0%	0.0%	2.1%	0.8%	6.4%
	Eukaryota;__Opisthokonta;__Fungi;__Nucletmycea	0	0.0%	0.0%	0.0%	0.0%	0.0%	0.0%	0.0%	0.0%	0.0%	0.0%	0.0%	0.0%	0.0%
	Eukaryota;__Opisthokonta;__Holozoa;__Choanomonada	0	0.0%	0.0%	0.0%	0.0%	0.0%	0.0%	0.0%	0.0%	0.0%	0.0%	0.0%	0.0%	0.0%
	Eukaryota;__Opisthokonta;__Metazoa;__Annelida	0	0.0%	0.0%	0.0%	0.0%	0.0%	0.0%	0.0%	0.0%	0.0%	0.0%	0.0%	0.0%	0.0%
	Eukaryota;__Opisthokonta;__Metazoa;__Arthropoda	0	0.0%	0.0%	0.0%	0.0%	0.0%	0.0%	0.0%	0.0%	0.0%	0.0%	0.0%	0.0%	0.0%
	Eukaryota;__Opisthokonta;__Metazoa;__Cnidaria	0	0.0%	0.0%	0.0%	0.0%	0.0%	0.0%	0.0%	0.0%	0.0%	0.0%	0.0%	0.0%	0.0%
	Eukaryota;__Opisthokonta;__Metazoa;__Gastrotricha	0	0.0%	0.0%	0.0%	0.0%	0.0%	0.0%	0.0%	0.0%	0.0%	0.0%	0.0%	0.0%	0.0%
	Eukaryota;__Opisthokonta;__Metazoa;__Nematoda	0	0.0%	0.0%	0.0%	0.0%	0.0%	0.0%	0.0%	0.0%	0.0%	0.0%	0.0%	0.0%	0.0%
	Eukaryota;__Opisthokonta;__Metazoa;__Platyhelminthes	0	0.0%	0.0%	0.0%	0.0%	0.0%	0.0%	0.0%	0.0%	0.0%	0.0%	0.0%	0.0%	0.0%
	Eukaryota;__Opisthokonta;__Metazoa;__Porifera	0	0.0%	0.0%	0.0%	0.0%	0.0%	0.0%	0.0%	0.0%	0.0%	0.0%	0.0%	0.0%	0.0%
	Eukaryota;__Opisthokonta;__Metazoa;__Rotifera	0	0.0%	0.0%	0.0%	0.0%	0.0%	0.0%	0.0%	0.0%	0.0%	0.0%	0.0%	0.0%	0.0%
	Eukaryota;__Opisthokonta;__Metazoa;__Tardigrada	0	0.0%	0.0%	0.0%	0.0%	0.0%	0.0%	0.0%	0.0%	0.0%	0.0%	0.0%	0.0%	0.0%
	Eukaryota;__Opisthokonta;__uncultured;__uncultured_Sarcosomataceae	0	0.0%	0.0%	0.0%	0.0%	0.0%	0.0%	0.0%	0.0%	0.0%	0.0%	0.0%	0.0%	0.0%
	Eukaryota;__RT5iin25;__uncultured_Eimeriidae;Other	0	0.0%	0.0%	0.0%	0.0%	0.0%	0.0%	0.0%	0.0%	0.0%	0.0%	0.0%	0.0%	0.0%
	Eukaryota;__RT5iin25;__uncultured_freshwater_eukaryote;Other	0	0.0%	0.0%	0.0%	0.0%	0.0%	0.0%	0.0%	0.0%	0.0%	0.0%	0.0%	0.0%	0.0%
	Eukaryota;__SAR;__Alveolata;__Apicomplexa	0	0.0%	0.0%	0.0%	0.0%	0.0%	0.0%	0.0%	0.0%	0.0%	0.0%	0.0%	0.0%	0.0%
	Eukaryota;__SAR;__Alveolata;__Ciliophora	0	2.6%	0.5%	6.3%	3.1%	0.3%	0.1%	0.1%	0.8%	0.2%	0.0%	14.4%	2.4%	3.3%
	Eukaryota;__SAR;__Alveolata;__Dinoflagellata	0	0.0%	0.0%	0.0%	0.0%	0.0%	0.0%	0.0%	0.0%	0.0%	0.0%	0.0%	0.0%	0.0%
	Eukaryota;__SAR;__Alveolata;__NIF-4C10	0	0.0%	0.0%	0.0%	0.0%	0.0%	0.0%	0.0%	0.0%	0.0%	0.0%	0.0%	0.0%	0.0%
	Eukaryota;__SAR;__Alveolata;__Protalveolata	0	0.0%	0.0%	0.0%	0.3%	0.0%	0.0%	0.0%	0.0%	0.0%	0.0%	0.0%	0.0%	0.0%
	Eukaryota;__SAR;__Rhizaria;__Cercozoa	1	10.9%	30.1%	42.5%	29.2%	1.9%	1.7%	1.9%	12.7%	2.7%	5.3%	0.9%	1.4%	0.5%
	Eukaryota;__SAR;__Stramenopiles;__Bicosoecida	0	0.0%	0.0%	0.0%	0.0%	0.0%	0.0%	0.0%	0.0%	0.0%	0.0%	0.0%	0.0%	0.0%
	Eukaryota;__SAR;__Stramenopiles;__CCI40	0	0.0%	0.0%	0.0%	0.0%	0.0%	0.0%	0.0%	0.0%	0.0%	0.0%	0.0%	0.3%	0.0%
	Eukaryota;__SAR;__Stramenopiles;__Chrysophyceae	2	13.1%	0.1%	0.6%	2.3%	3.4%	1.1%	0.0%	0.0%	7.8%	4.3%	55.0%	30.0%	52.4%
	Eukaryota;__SAR;__Stramenopiles;__Diatomea	0	0.0%	0.0%	0.0%	0.0%	0.0%	0.1%	0.0%	0.0%	0.0%	0.0%	0.0%	0.0%	0.0%
	Eukaryota;__SAR;__Stramenopiles;__Eustigmatales	0	0.0%	0.0%	0.0%	0.0%	0.0%	0.0%	0.0%	0.0%	0.0%	0.0%	0.0%	0.0%	0.0%
	Eukaryota;__SAR;__Stramenopiles;__Labyrinthulomycetes	0	0.0%	0.0%	0.0%	0.0%	0.0%	0.0%	0.0%	0.0%	0.0%	0.0%	0.0%	0.0%	0.0%
	Eukaryota;__SAR;__Stramenopiles;__Peronosporomycetes	0	0.0%	0.0%	0.0%	0.0%	0.0%	0.0%	0.0%	0.0%	0.0%	0.0%	0.0%	0.0%	0.0%
	Eukaryota;__SAR;__Stramenopiles;__Synurales	0	0.1%	0.0%	0.0%	0.0%	0.0%	0.0%	0.0%	0.4%	0.0%	0.0%	0.0%	0.4%	0.0%
	Eukaryota;__SAR;__Stramenopiles;__TKR07M.92	0	0.0%	0.0%	0.0%	0.0%	0.0%	0.0%	0.0%	0.0%	0.0%	0.0%	0.0%	0.0%	0.0%
	Eukaryota;__SAR;__Stramenopiles;__Xanthophyceae	0	0.0%	0.0%	0.2%	0.0%	0.0%	0.0%	0.2%	0.0%	0.0%	0.0%	0.0%	0.0%	0.0%
	Eukaryota;__SAR;__Stramenopiles;__uncultured_eukaryote	0	0.8%	0.0%	0.0%	0.0%	0.0%	7.6%	0.3%	1.9%	0.0%	0.0%	0.0%	0.0%	0.0%
	Eukaryota;__Zeuk77;__uncultured_Oxytrichidae;Other	0	0.1%	0.1%	1.4%	0.2%	0.0%	0.0%	0.0%	0.0%	0.0%	0.0%	0.0%	0.0%	0.0%
	Eukaryota;__Zeuk77;__uncultured_eukaryote;Other	0	0.1%	0.0%	0.2%	0.0%	0.0%	0.1%	0.0%	0.0%	0.0%	0.0%	0.4%	0.2%	0.1%
	No blast hit;Other;Other;Other	0	0.2%	0.0%	0.0%	0.0%	0.0%	0.1%	0.0%	0.3%	0.0%	0.0%	0.2%	0.9%	1.0%

Taxonomy Summary. Current Level:

View Figure (.pdf) View Legend (.pdf)

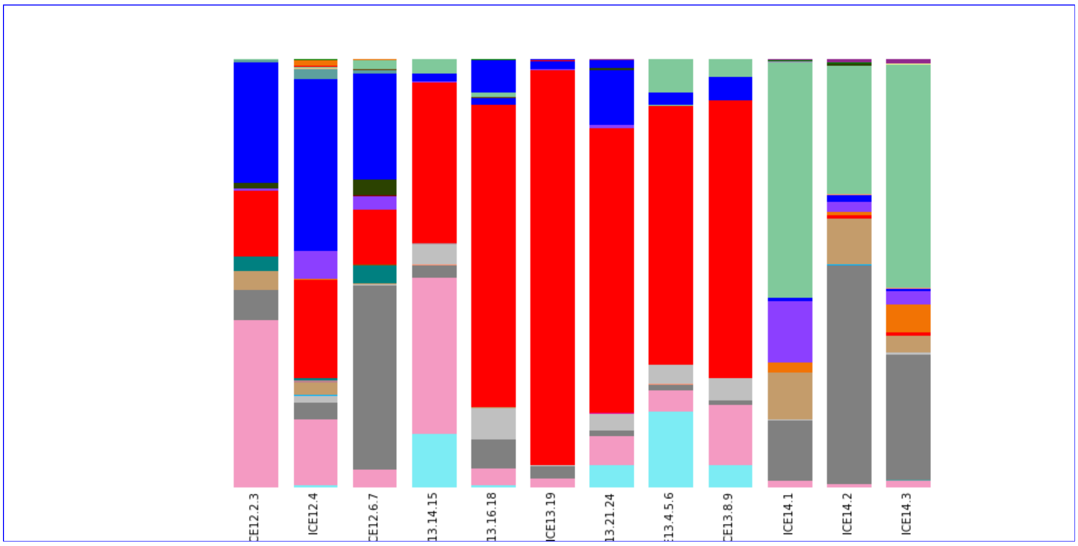

View Table (.txt)

		Total		ICE12.2.3	ICE12.4	ICE12.6.7	ICE13.14.15	ICE13.16.18	ICE13.19	ICE13.21.24	ICE13.4.5.6	ICE13.8.9	ICE14.1	ICE14.2	ICE14.3
Legend	Taxonomy	count	%	%	%	%	%	%	%	%	%	%	%	%	%
	Eukaryota;__Amoebozoa;__Conosa;__Schizoplasmodiida;__Ceratiomyxella	0	0.0%	0.0%	0.0%	0.0%	0.0%	0.0%	0.0%	0.0%	0.0%	0.0%	0.0%	0.0%	0.0%
	Eukaryota;__Amoebozoa;__Conosa;__Schizoplasmodiida;__Nematostelium	0	0.0%	0.0%	0.0%	0.0%	0.0%	0.0%	0.0%	0.0%	0.0%	0.0%	0.0%	0.0%	0.0%
	Eukaryota;__Amoebozoa;__Conosa;__Soliformovum;__Soliformovum_irregulare	0	0.0%	0.0%	0.0%	0.0%	0.0%	0.0%	0.0%	0.0%	0.0%	0.0%	0.0%	0.0%	0.0%
	Eukaryota;__Amoebozoa;__Discosea;__Flabellinia;__Dactylopodida	0	0.0%	0.0%	0.0%	0.0%	0.0%	0.0%	0.0%	0.0%	0.0%	0.0%	0.0%	0.0%	0.0%
	Eukaryota;__Amoebozoa;__Lobosa;__Tubulinea;__Euamoebida	0	0.0%	0.0%	0.0%	0.0%	0.0%	0.0%	0.0%	0.0%	0.0%	0.0%	0.0%	0.0%	0.0%
	Eukaryota;__Archaeplastida;__Chloroplastida;__Charophyta;__Amb-18S-504	0	0.0%	0.0%	0.0%	0.0%	0.0%	0.0%	0.0%	0.0%	0.0%	0.0%	0.0%	0.0%	0.0%
	Eukaryota;__Archaeplastida;__Chloroplastida;__Charophyta;__Phragmoplastophyta	0	3.5%	0.0%	0.6%	0.0%	12.4%	0.5%	0.0%	5.2%	17.6%	5.3%	0.0%	0.0%	0.0%
	Eukaryota;__Archaeplastida;__Chloroplastida;__Chlorophyta;__Chlorophyceae	1	10.9%	39.0%	15.3%	4.2%	36.5%	3.9%	2.1%	6.8%	4.9%	13.8%	1.6%	0.7%	1.6%
	Eukaryota;__Archaeplastida;__Chloroplastida;__Chlorophyta;__Mamiellophyceae	0	0.0%	0.0%	0.0%	0.0%	0.0%	0.0%	0.0%	0.0%	0.0%	0.0%	0.1%	0.1%	0.2%
	Eukaryota;__Archaeplastida;__Chloroplastida;__Chlorophyta;__Nephroselmidophyceae	0	0.0%	0.0%	0.0%	0.0%	0.0%	0.0%	0.0%	0.0%	0.0%	0.0%	0.0%	0.0%	0.0%
	Eukaryota;__Archaeplastida;__Chloroplastida;__Chlorophyta;__Trebouxiophyceae	2	13.7%	7.1%	4.0%	43.1%	3.0%	6.7%	2.9%	1.4%	1.4%	1.1%	14.1%	51.1%	29.1%
	Eukaryota;__Archaeplastida;__Chloroplastida;__Chlorophyta;__Ulvophyceae	0	0.0%	0.0%	0.0%	0.0%	0.0%	0.0%	0.0%	0.0%	0.1%	0.0%	0.0%	0.0%	0.0%
	Eukaryota;__Archaeplastida;__Chloroplastida;__uncultured;__Jatropha	0	0.0%	0.0%	0.0%	0.0%	0.1%	0.0%	0.0%	0.0%	0.1%	0.0%	0.0%	0.0%	0.0%
	Eukaryota;__Archaeplastida;__Chloroplastida;__uncultured;__Neosetophoma	0	0.0%	0.0%	0.0%	0.0%	0.0%	0.0%	0.0%	0.0%	0.0%	0.0%	0.0%	0.0%	0.0%
	Eukaryota;__Archaeplastida;__Chloroplastida;__uncultured;__uncultured_Ascomycota	0	0.0%	0.0%	0.0%	0.0%	0.0%	0.0%	0.0%	0.0%	0.0%	0.0%	0.0%	0.0%	0.0%
	Eukaryota;__Archaeplastida;__Rhodophyceae;__Compsopogonales;__Compsopogon	0	0.0%	0.0%	0.0%	0.0%	0.0%	0.0%	0.0%	0.0%	0.0%	0.0%	0.0%	0.0%	0.0%
	Eukaryota;__Centrohelida;__Heterophryidae;__Heterophrys;__Heterophrys_sp._Oxford1	0	0.0%	0.0%	0.0%	0.0%	0.0%	0.0%	0.0%	0.0%	0.0%	0.0%	0.0%	0.0%	0.0%
	Eukaryota;__Kathablepharidae;__Roombia;__Roombia_truncata;Other	0	0.0%	0.0%	0.0%	0.0%	0.0%	0.0%	0.0%	0.0%	0.0%	0.0%	0.0%	0.0%	0.0%
	Eukaryota;__Opisthokonta;__Fungi;__Ascomycota;__Pezizomycotina	0	2.3%	0.0%	1.4%	0.1%	4.9%	7.2%	0.2%	3.8%	4.5%	5.3%	0.2%	0.0%	0.5%
	Eukaryota;__Opisthokonta;__Fungi;__Ascomycota;__Taphrinomycotina	0	0.0%	0.0%	0.0%	0.0%	0.0%	0.0%	0.0%	0.1%	0.0%	0.0%	0.0%	0.0%	0.0%
	Eukaryota;__Opisthokonta;__Fungi;__Basal_fungi;__Blastocladiomycota	0	0.0%	0.0%	0.4%	0.1%	0.0%	0.0%	0.0%	0.0%	0.0%	0.0%	0.0%	0.1%	0.0%
	Eukaryota;__Opisthokonta;__Fungi;__Basal_fungi;__Chytridiomycota	0	2.8%	4.6%	3.0%	0.3%	0.0%	0.4%	0.0%	0.0%	0.0%	0.0%	10.8%	10.7%	3.8%
	Eukaryota;__Opisthokonta;__Fungi;__Basal_fungi;__Entomophthoromycotina	0	0.0%	0.0%	0.0%	0.0%	0.0%	0.0%	0.0%	0.0%	0.0%	0.0%	0.0%	0.0%	0.0%
	Eukaryota;__Opisthokonta;__Fungi;__Basal_fungi;__Glomeromycota	0	0.0%	0.0%	0.0%	0.0%	0.0%	0.0%	0.0%	0.0%	0.0%	0.0%	0.0%	0.0%	0.0%
	Eukaryota;__Opisthokonta;__Fungi;__Basal_fungi;__Mucoromycotina	0	0.0%	0.0%	0.4%	0.0%	0.1%	0.0%	0.0%	0.0%	0.0%	0.0%	0.0%	0.0%	0.0%
	Eukaryota;__Opisthokonta;__Fungi;__Basal_fungi;__uncultured	0	0.7%	3.3%	0.6%	4.2%	0.0%	0.0%	0.0%	0.0%	0.0%	0.0%	0.1%	0.1%	0.1%
	Eukaryota;__Opisthokonta;__Fungi;__Basal_fungi;__uncultured_Chytridiomycota	0	0.0%	0.0%	0.0%	0.0%	0.0%	0.0%	0.0%	0.0%	0.0%	0.0%	0.0%	0.0%	0.0%
	Eukaryota;__Opisthokonta;__Fungi;__Basal_fungi;__uncultured_eukaryote	0	0.0%	0.0%	0.0%	0.0%	0.0%	0.0%	0.0%	0.0%	0.0%	0.0%	0.0%	0.0%	0.0%
	Eukaryota;__Opisthokonta;__Fungi;__Basidiomycota;__Agaricomycotina	0	0.0%	0.0%	0.0%	0.3%	0.0%	0.0%	0.0%	0.0%	0.0%	0.0%	0.0%	0.0%	0.0%
	Eukaryota;__Opisthokonta;__Fungi;__Basidiomycota;__Pucciniomycotina	4	37.1%	15.3%	22.8%	12.9%	37.5%	70.5%	92.2%	66.6%	60.4%	64.9%	0.1%	0.7%	0.8%
	Eukaryota;__Opisthokonta;__Fungi;__LKM11;__uncultured_fungus	0	0.0%	0.0%	0.0%	0.0%	0.0%	0.0%	0.1%	0.0%	0.0%	0.0%	0.0%	0.0%	0.0%
	Eukaryota;__Opisthokonta;__Fungi;__LKM15;__uncultured_Clathrinidae_sp.	0	0.8%	0.0%	0.4%	0.0%	0.0%	0.0%	0.0%	0.0%	0.0%	0.0%	2.1%	0.8%	6.4%
	Eukaryota;__Opisthokonta;__Fungi;__Nucletmycea;__Fonticula	0	0.0%	0.0%	0.0%	0.0%	0.0%	0.0%	0.0%	0.0%	0.0%	0.0%	0.0%	0.0%	0.0%
	Eukaryota;__Opisthokonta;__Holozoa;__Choanomonada;__Acanthoecida	0	0.0%	0.0%	0.0%	0.0%	0.0%	0.0%	0.0%	0.0%	0.0%	0.0%	0.0%	0.0%	0.0%
	Eukaryota;__Opisthokonta;__Holozoa;__Choanomonada;__Craspedida	0	0.0%	0.0%	0.0%	0.0%	0.0%	0.0%	0.0%	0.0%	0.0%	0.0%	0.0%	0.0%	0.0%
	Eukaryota;__Opisthokonta;__Metazoa;__Annelida;__Family_Incertae_Sedis	0	0.0%	0.0%	0.0%	0.0%	0.0%	0.0%	0.0%	0.0%	0.0%	0.0%	0.0%	0.0%	0.0%
	Eukaryota;__Opisthokonta;__Metazoa;__Arthropoda;__Chelicerata	0	0.0%	0.0%	0.0%	0.0%	0.0%	0.0%	0.0%	0.0%	0.0%	0.0%	0.0%	0.0%	0.0%
	Eukaryota;__Opisthokonta;__Metazoa;__Arthropoda;__Crustacea	0	0.0%	0.0%	0.0%	0.0%	0.0%	0.0%	0.0%	0.0%	0.0%	0.0%	0.0%	0.0%	0.0%
	Eukaryota;__Opisthokonta;__Metazoa;__Arthropoda;__Hexapoda	0	0.0%	0.0%	0.0%	0.0%	0.0%	0.0%	0.0%	0.0%	0.0%	0.0%	0.0%	0.0%	0.0%
	Eukaryota;__Opisthokonta;__Metazoa;__Cnidaria;__Anthozoa	0	0.0%	0.0%	0.0%	0.0%	0.0%	0.0%	0.0%	0.0%	0.0%	0.0%	0.0%	0.0%	0.0%
	Eukaryota;__Opisthokonta;__Metazoa;__Gastrotricha;__Thaumastodermatidae	0	0.0%	0.0%	0.0%	0.0%	0.0%	0.0%	0.0%	0.0%	0.0%	0.0%	0.0%	0.0%	0.0%
	Eukaryota;__Opisthokonta;__Metazoa;__Nematoda;__Chromadorea	0	0.0%	0.0%	0.0%	0.0%	0.0%	0.0%	0.0%	0.0%	0.0%	0.0%	0.0%	0.0%	0.0%
	Eukaryota;__Opisthokonta;__Metazoa;__Platyhelminthes;__Turbellaria	0	0.0%	0.0%	0.0%	0.0%	0.0%	0.0%	0.0%	0.0%	0.0%	0.0%	0.0%	0.0%	0.0%
	Eukaryota;__Opisthokonta;__Metazoa;__Porifera;__Calcarea	0	0.0%	0.0%	0.0%	0.0%	0.0%	0.0%	0.0%	0.0%	0.0%	0.0%	0.0%	0.0%	0.0%
	Eukaryota;__Opisthokonta;__Metazoa;__Porifera;__Hexactinellida	0	0.0%	0.0%	0.0%	0.0%	0.0%	0.0%	0.0%	0.0%	0.0%	0.0%	0.0%	0.0%	0.0%
	Eukaryota;__Opisthokonta;__Metazoa;__Rotifera;__Family_Incertae_Sedis	0	0.0%	0.0%	0.0%	0.0%	0.0%	0.0%	0.0%	0.0%	0.0%	0.0%	0.0%	0.0%	0.0%
	Eukaryota;__Opisthokonta;__Metazoa;__Rotifera;__Philodinidae	0	0.0%	0.0%	0.0%	0.0%	0.0%	0.0%	0.0%	0.0%	0.0%	0.0%	0.0%	0.0%	0.0%
	Eukaryota;__Opisthokonta;__Metazoa;__Tardigrada;__Hypsibiidae	0	0.0%	0.0%	0.0%	0.0%	0.0%	0.0%	0.0%	0.0%	0.0%	0.0%	0.0%	0.0%	0.0%
	Eukaryota;__Opisthokonta;__uncultured;__uncultured_Sarcosomataceae;Other	0	0.0%	0.0%	0.0%	0.0%	0.0%	0.0%	0.0%	0.0%	0.0%	0.0%	0.0%	0.0%	0.0%
	Eukaryota;__RT5iin25;__uncultured_Eimeriidae;Other;Other	0	0.0%	0.0%	0.0%	0.0%	0.0%	0.0%	0.0%	0.0%	0.0%	0.0%	0.0%	0.0%	0.0%
	Eukaryota;__RT5iin25;__uncultured_freshwater_eukaryote;Other;Other	0	0.0%	0.0%	0.0%	0.0%	0.0%	0.0%	0.0%	0.0%	0.0%	0.0%	0.0%	0.0%	0.0%
	Eukaryota;__SAR;__Alveolata;__Apicomplexa;__Conoidasida	0	0.0%	0.0%	0.0%	0.0%	0.0%	0.0%	0.0%	0.0%	0.0%	0.0%	0.0%	0.0%	0.0%
	Eukaryota;__SAR;__Alveolata;__Ciliophora;__Intramacronucleata	0	2.6%	0.5%	6.3%	3.1%	0.3%	0.1%	0.1%	0.8%	0.2%	0.0%	14.4%	2.4%	3.3%
	Eukaryota;__SAR;__Alveolata;__Dinoflagellata;__Dinophyceae	0	0.0%	0.0%	0.0%	0.0%	0.0%	0.0%	0.0%	0.0%	0.0%	0.0%	0.0%	0.0%	0.0%
	Eukaryota;__SAR;__Alveolata;__NIF-4C10;__uncultured_marine_alveolate	0	0.0%	0.0%	0.0%	0.0%	0.0%	0.0%	0.0%	0.0%	0.0%	0.0%	0.0%	0.0%	0.0%
	Eukaryota;__SAR;__Alveolata;__Protalveolata;__Perkinsidae	0	0.0%	0.0%	0.0%	0.3%	0.0%	0.0%	0.0%	0.0%	0.0%	0.0%	0.0%	0.0%	0.0%
	Eukaryota;__SAR;__Rhizaria;__Cercozoa;__Athalamea	0	0.4%	1.5%	0.0%	3.5%	0.0%	0.0%	0.0%	0.0%	0.0%	0.0%	0.0%	0.0%	0.0%
	Eukaryota;__SAR;__Rhizaria;__Cercozoa;__Cercomonadidae	0	0.0%	0.0%	0.0%	0.1%	0.0%	0.0%	0.0%	0.0%	0.0%	0.0%	0.0%	0.0%	0.0%
	Eukaryota;__SAR;__Rhizaria;__Cercozoa;__Endomyxa	0	0.0%	0.0%	0.0%	0.0%	0.0%	0.0%	0.0%	0.0%	0.0%	0.0%	0.0%	0.0%	0.0%
	Eukaryota;__SAR;__Rhizaria;__Cercozoa;__Glissomonadida	1	10.1%	28.1%	40.1%	24.7%	1.9%	1.6%	1.8%	12.6%	2.7%	5.3%	0.9%	1.4%	0.5%
	Eukaryota;__SAR;__Rhizaria;__Cercozoa;__Incertae_Sedis	0	0.0%	0.0%	0.0%	0.0%	0.0%	0.0%	0.0%	0.0%	0.0%	0.0%	0.0%	0.0%	0.0%
	Eukaryota;__SAR;__Rhizaria;__Cercozoa;__Metromonadea	0	0.0%	0.0%	0.0%	0.0%	0.0%	0.0%	0.0%	0.0%	0.0%	0.0%	0.0%	0.0%	0.0%
	Eukaryota;__SAR;__Rhizaria;__Cercozoa;__Novel_Clade_12	0	0.0%	0.0%	0.0%	0.0%	0.0%	0.0%	0.0%	0.0%	0.0%	0.0%	0.0%	0.0%	0.0%
	Eukaryota;__SAR;__Rhizaria;__Cercozoa;__PP1-8	0	0.0%	0.0%	0.0%	0.0%	0.0%	0.0%	0.0%	0.0%	0.0%	0.0%	0.0%	0.0%	0.0%
	Eukaryota;__SAR;__Rhizaria;__Cercozoa;__Pansomonadida	0	0.0%	0.0%	0.0%	0.1%	0.0%	0.0%	0.0%	0.0%	0.0%	0.0%	0.0%	0.0%	0.0%
	Eukaryota;__SAR;__Rhizaria;__Cercozoa;__Silicofilosea	0	0.0%	0.0%	0.2%	0.0%	0.0%	0.0%	0.0%	0.1%	0.0%	0.0%	0.0%	0.0%	0.0%
	Eukaryota;__SAR;__Rhizaria;__Cercozoa;__Thecofilosea	0	0.3%	0.6%	2.2%	0.8%	0.0%	0.1%	0.1%	0.0%	0.0%	0.0%	0.0%	0.0%	0.1%
	Eukaryota;__SAR;__Rhizaria;__Cercozoa;__uncultured	0	0.0%	0.0%	0.0%	0.1%	0.0%	0.1%	0.0%	0.0%	0.0%	0.0%	0.0%	0.0%	0.0%
	Eukaryota;__SAR;__Stramenopiles;__Bicosoecida;__P34.6	0	0.0%	0.0%	0.0%	0.0%	0.0%	0.0%	0.0%	0.0%	0.0%	0.0%	0.0%	0.0%	0.0%
	Eukaryota;__SAR;__Stramenopiles;__Bicosoecida;__Siluaniidae	0	0.0%	0.0%	0.0%	0.0%	0.0%	0.0%	0.0%	0.0%	0.0%	0.0%	0.0%	0.0%	0.0%
	Eukaryota;__SAR;__Stramenopiles;__CCI40;__uncultured_marine_eukaryote	0	0.0%	0.0%	0.0%	0.0%	0.0%	0.0%	0.0%	0.0%	0.0%	0.0%	0.0%	0.3%	0.0%
	Eukaryota;__SAR;__Stramenopiles;__Chrysophyceae;__CCMP1899	0	0.0%	0.0%	0.0%	0.0%	0.0%	0.0%	0.0%	0.0%	0.0%	0.0%	0.0%	0.0%	0.0%
	Eukaryota;__SAR;__Stramenopiles;__Chrysophyceae;__Chromulinales	0	0.0%	0.0%	0.4%	0.1%	0.0%	0.0%	0.0%	0.0%	0.0%	0.0%	0.0%	0.0%	0.0%
	Eukaryota;__SAR;__Stramenopiles;__Chrysophyceae;__Chrysocapsales	2	13.0%	0.1%	0.2%	2.2%	3.4%	1.1%	0.0%	0.0%	7.8%	4.3%	55.0%	30.0%	52.3%
	Eukaryota;__SAR;__Stramenopiles;__Chrysophyceae;__LG21-05	0	0.0%	0.0%	0.0%	0.0%	0.0%	0.0%	0.0%	0.0%	0.0%	0.0%	0.0%	0.0%	0.0%
	Eukaryota;__SAR;__Stramenopiles;__Chrysophyceae;__Ochromonadales	0	0.0%	0.0%	0.0%	0.0%	0.0%	0.0%	0.0%	0.0%	0.0%	0.0%	0.0%	0.0%	0.0%
	Eukaryota;__SAR;__Stramenopiles;__Chrysophyceae;__uncultured_eukaryote	0	0.0%	0.0%	0.0%	0.0%	0.0%	0.0%	0.0%	0.0%	0.0%	0.0%	0.0%	0.0%	0.0%
	Eukaryota;__SAR;__Stramenopiles;__Diatomea;__Bacillariophytina	0	0.0%	0.0%	0.0%	0.0%	0.0%	0.1%	0.0%	0.0%	0.0%	0.0%	0.0%	0.0%	0.0%
	Eukaryota;__SAR;__Stramenopiles;__Eustigmatales;__Nannochloropsis	0	0.0%	0.0%	0.0%	0.0%	0.0%	0.0%	0.0%	0.0%	0.0%	0.0%	0.0%	0.0%	0.0%
	Eukaryota;__SAR;__Stramenopiles;__Labyrinthulomycetes;__Thraustochytriaceae	0	0.0%	0.0%	0.0%	0.0%	0.0%	0.0%	0.0%	0.0%	0.0%	0.0%	0.0%	0.0%	0.0%
	Eukaryota;__SAR;__Stramenopiles;__Peronosporomycetes;__Aphanomyces	0	0.0%	0.0%	0.0%	0.0%	0.0%	0.0%	0.0%	0.0%	0.0%	0.0%	0.0%	0.0%	0.0%
	Eukaryota;__SAR;__Stramenopiles;__Peronosporomycetes;__Lagenidium	0	0.0%	0.0%	0.0%	0.0%	0.0%	0.0%	0.0%	0.0%	0.0%	0.0%	0.0%	0.0%	0.0%

	Eukaryota;__SAR;__Stramenopiles;__Peronosporomycetes;__Ochromonadaceae_environmental_sample	0	0.0%	0.0%	0.0%	0.0%	0.0%	0.0%	0.0%	0.0%	0.0%	0.0%	0.0%	0.0%	0.0%
	Eukaryota;__SAR;__Stramenopiles;__Peronosporomycetes;__Phytophthora	0	0.0%	0.0%	0.0%	0.0%	0.0%	0.0%	0.0%	0.0%	0.0%	0.0%	0.0%	0.0%	0.0%
	Eukaryota;__SAR;__Stramenopiles;__Peronosporomycetes;__Saprolegnia	0	0.0%	0.0%	0.0%	0.0%	0.0%	0.0%	0.0%	0.0%	0.0%	0.0%	0.0%	0.0%	0.0%
	Eukaryota;__SAR;__Stramenopiles;__Synurales;__Synura	0	0.1%	0.0%	0.0%	0.0%	0.0%	0.0%	0.0%	0.4%	0.0%	0.0%	0.0%	0.4%	0.0%
	Eukaryota;__SAR;__Stramenopiles;__TKR07M.92;__uncultured_eukaryote	0	0.0%	0.0%	0.0%	0.0%	0.0%	0.0%	0.0%	0.0%	0.0%	0.0%	0.0%	0.0%	0.0%
	Eukaryota;__SAR;__Stramenopiles;__Xanthophyceae;__Tribonematales	0	0.0%	0.0%	0.2%	0.0%	0.0%	0.0%	0.2%	0.0%	0.0%	0.0%	0.0%	0.0%	0.0%
	Eukaryota;__SAR;__Stramenopiles;__uncultured_eukaryote;Other	0	0.8%	0.0%	0.0%	0.0%	0.0%	7.6%	0.3%	1.9%	0.0%	0.0%	0.0%	0.0%	0.0%
	Eukaryota;__Zeuk77;__uncultured_Oxytrichidae;Other;Other	0	0.1%	0.1%	1.4%	0.2%	0.0%	0.0%	0.0%	0.0%	0.0%	0.0%	0.0%	0.0%	0.0%
	Eukaryota;__Zeuk77;__uncultured_eukaryote;Other;Other	0	0.1%	0.0%	0.2%	0.0%	0.0%	0.1%	0.0%	0.0%	0.0%	0.0%	0.4%	0.2%	0.1%
	No blast hit;Other;Other;Other;Other	0	0.2%	0.0%	0.0%	0.0%	0.0%	0.1%	0.0%	0.3%	0.0%	0.0%	0.2%	0.9%	1.0%

Taxonomy Summary. Current Level:

View Figure (.pdf) View Legend (.pdf)

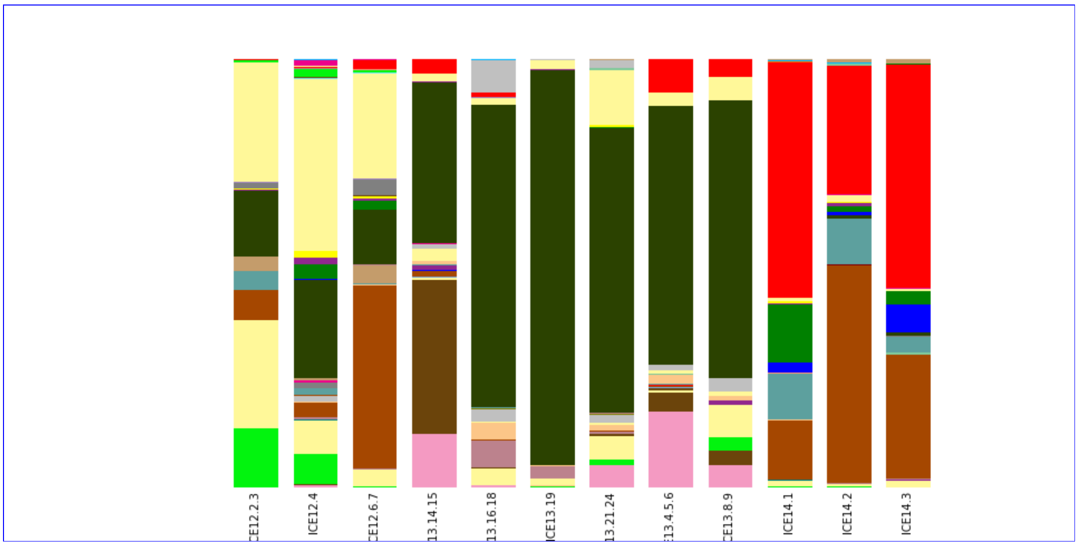

View Table (.txt)

		Total		ICE12.2.3	ICE12.4	ICE12.6.7	ICE13.14.15	ICE13.16.18	ICE13.19	ICE13.21.24	ICE13.4.5.6	ICE13.8.9	ICE14.1	ICE14.2	ICE14.3
Legend	Taxonomy	count	%	%	%	%	%	%	%	%	%	%	%	%	%
	Eukaryota;__Amoebozoa;__Conosa;__Schizoplasmodiida;__Ceratiomyxella;__Ceratiomyxella_tahitiensis	0	0.0%	0.0%	0.0%	0.0%	0.0%	0.0%	0.0%	0.0%	0.0%	0.0%	0.0%	0.0%	0.0%
	Eukaryota;__Amoebozoa;__Conosa;__Schizoplasmodiida;__Nematostelium;__Nematostelium_ovatum	0	0.0%	0.0%	0.0%	0.0%	0.0%	0.0%	0.0%	0.0%	0.0%	0.0%	0.0%	0.0%	0.0%
	Eukaryota;__Amoebozoa;__Conosa;__Soliformovum;__Soliformovum_irregulare;Other	0	0.0%	0.0%	0.0%	0.0%	0.0%	0.0%	0.0%	0.0%	0.0%	0.0%	0.0%	0.0%	0.0%
	Eukaryota;__Amoebozoa;__Discosea;__Flabellinia;__Dactylopodida;__A1WVB	0	0.0%	0.0%	0.0%	0.0%	0.0%	0.0%	0.0%	0.0%	0.0%	0.0%	0.0%	0.0%	0.0%
	Eukaryota;__Amoebozoa;__Lobosa;__Tubulinea;__Euamoebida;__BOLA868	0	0.0%	0.0%	0.0%	0.0%	0.0%	0.0%	0.0%	0.0%	0.0%	0.0%	0.0%	0.0%	0.0%
	Eukaryota;__Archaeplastida;__Chloroplastida;__Charophyta;__Amb-18S-504;__Phaseoleae	0	0.0%	0.0%	0.0%	0.0%	0.0%	0.0%	0.0%	0.0%	0.0%	0.0%	0.0%	0.0%	0.0%
	Eukaryota;__Archaeplastida;__Chloroplastida;__Charophyta;__Phragmoplastophyta;__Coleochaetophyceae	0	0.0%	0.0%	0.0%	0.0%	0.0%	0.0%	0.0%	0.0%	0.0%	0.0%	0.0%	0.0%	0.0%
	Eukaryota;__Archaeplastida;__Chloroplastida;__Charophyta;__Phragmoplastophyta;__Streptophyta	0	3.5%	0.0%	0.6%	0.0%	12.4%	0.5%	0.0%	5.2%	17.6%	5.3%	0.0%	0.0%	0.0%
	Eukaryota;__Archaeplastida;__Chloroplastida;__Charophyta;__Phragmoplastophyta;__Zygnematales	0	0.0%	0.0%	0.0%	0.0%	0.0%	0.0%	0.0%	0.0%	0.0%	0.0%	0.0%	0.0%	0.0%
	Eukaryota;__Archaeplastida;__Chloroplastida;__Chlorophyta;__Chlorophyceae;__Amb-18S-422	0	3.7%	0.0%	0.2%	0.0%	35.9%	0.0%	0.0%	0.1%	4.5%	3.2%	0.0%	0.0%	0.0%
	Eukaryota;__Archaeplastida;__Chloroplastida;__Chlorophyta;__Chlorophyceae;__Asterococcus	0	0.0%	0.0%	0.0%	0.0%	0.0%	0.0%	0.0%	0.0%	0.0%	0.0%	0.0%	0.0%	0.0%
	Eukaryota;__Archaeplastida;__Chloroplastida;__Chlorophyta;__Chlorophyceae;__CCCryo	0	2.2%	13.9%	6.9%	0.3%	0.0%	0.1%	0.2%	1.1%	0.0%	3.2%	0.2%	0.1%	0.0%
	Eukaryota;__Archaeplastida;__Chloroplastida;__Chlorophyta;__Chlorophyceae;__Carteria	0	0.0%	0.0%	0.0%	0.1%	0.0%	0.0%	0.0%	0.0%	0.0%	0.0%	0.0%	0.0%	0.0%
	Eukaryota;__Archaeplastida;__Chloroplastida;__Chlorophyta;__Chlorophyceae;__Characiochloris	0	0.0%	0.0%	0.0%	0.0%	0.0%	0.0%	0.0%	0.0%	0.0%	0.0%	0.0%	0.0%	0.0%
	Eukaryota;__Archaeplastida;__Chloroplastida;__Chlorophyta;__Chlorophyceae;__Chlamydomonas	0	0.0%	0.0%	0.0%	0.0%	0.0%	0.0%	0.2%	0.0%	0.0%	0.0%	0.0%	0.0%	0.0%
	Eukaryota;__Archaeplastida;__Chloroplastida;__Chlorophyta;__Chlorophyceae;__Chlorococcum	0	0.0%	0.0%	0.0%	0.0%	0.0%	0.0%	0.0%	0.0%	0.0%	0.0%	0.0%	0.0%	0.0%
	Eukaryota;__Archaeplastida;__Chloroplastida;__Chlorophyta;__Chlorophyceae;__Chlorogonium	0	0.0%	0.0%	0.0%	0.0%	0.0%	0.0%	0.0%	0.0%	0.0%	0.0%	0.0%	0.0%	0.0%
	Eukaryota;__Archaeplastida;__Chloroplastida;__Chlorophyta;__Chlorophyceae;__Chloromonas	1	5.0%	25.0%	7.9%	3.7%	0.6%	3.8%	1.7%	5.6%	0.4%	7.4%	1.3%	0.5%	1.5%
	Eukaryota;__Archaeplastida;__Chloroplastida;__Chlorophyta;__Chlorophyceae;__Chlorosarcinopsis	0	0.0%	0.0%	0.0%	0.0%	0.0%	0.0%	0.0%	0.0%	0.0%	0.0%	0.0%	0.0%	0.0%
	Eukaryota;__Archaeplastida;__Chloroplastida;__Chlorophyta;__Chlorophyceae;__Dictyococcus	0	0.0%	0.0%	0.0%	0.1%	0.0%	0.0%	0.0%	0.0%	0.0%	0.0%	0.0%	0.0%	0.0%
	Eukaryota;__Archaeplastida;__Chloroplastida;__Chlorophyta;__Chlorophyceae;__Dunaliella	0	0.0%	0.0%	0.0%	0.0%	0.0%	0.0%	0.0%	0.0%	0.0%	0.0%	0.0%	0.0%	0.0%
	Eukaryota;__Archaeplastida;__Chloroplastida;__Chlorophyta;__Chlorophyceae;__Dysmorphococcus	0	0.0%	0.0%	0.0%	0.0%	0.0%	0.0%	0.0%	0.0%	0.0%	0.0%	0.0%	0.0%	0.0%
	Eukaryota;__Archaeplastida;__Chloroplastida;__Chlorophyta;__Chlorophyceae;__E108-01D	0	0.0%	0.0%	0.0%	0.0%	0.0%	0.0%	0.0%	0.0%	0.0%	0.0%	0.0%	0.0%	0.0%
	Eukaryota;__Archaeplastida;__Chloroplastida;__Chlorophyta;__Chlorophyceae;__Elev-18S-713	0	0.0%	0.0%	0.0%	0.0%	0.0%	0.0%	0.0%	0.0%	0.0%	0.0%	0.0%	0.0%	0.0%
	Eukaryota;__Archaeplastida;__Chloroplastida;__Chlorophyta;__Chlorophyceae;__Fritschiella	0	0.0%	0.0%	0.0%	0.0%	0.0%	0.0%	0.0%	0.0%	0.0%	0.0%	0.0%	0.0%	0.0%
	Eukaryota;__Archaeplastida;__Chloroplastida;__Chlorophyta;__Chlorophyceae;__Hafniomonas	0	0.0%	0.0%	0.2%	0.0%	0.0%	0.0%	0.0%	0.0%	0.0%	0.0%	0.0%	0.0%	0.0%
	Eukaryota;__Archaeplastida;__Chloroplastida;__Chlorophyta;__Chlorophyceae;__Lobochlamys	0	0.0%	0.0%	0.0%	0.0%	0.0%	0.0%	0.0%	0.0%	0.0%	0.0%	0.0%	0.0%	0.0%
	Eukaryota;__Archaeplastida;__Chloroplastida;__Chlorophyta;__Chlorophyceae;__Neochlorosarcina	0	0.0%	0.0%	0.0%	0.0%	0.0%	0.0%	0.0%	0.0%	0.0%	0.0%	0.0%	0.0%	0.0%
	Eukaryota;__Archaeplastida;__Chloroplastida;__Chlorophyta;__Chlorophyceae;__Neocystis	0	0.0%	0.0%	0.0%	0.0%	0.0%	0.0%	0.0%	0.0%	0.0%	0.0%	0.0%	0.0%	0.0%
	Eukaryota;__Archaeplastida;__Chloroplastida;__Chlorophyta;__Chlorophyceae;__Oogamochlamys	0	0.0%	0.0%	0.0%	0.0%	0.0%	0.0%	0.0%	0.0%	0.0%	0.0%	0.0%	0.0%	0.0%
	Eukaryota;__Archaeplastida;__Chloroplastida;__Chlorophyta;__Chlorophyceae;__Paulschulzia	0	0.0%	0.0%	0.0%	0.0%	0.0%	0.0%	0.0%	0.0%	0.0%	0.0%	0.0%	0.0%	0.0%
	Eukaryota;__Archaeplastida;__Chloroplastida;__Chlorophyta;__Chlorophyceae;__Pseudopediastrum	0	0.0%	0.0%	0.0%	0.0%	0.0%	0.0%	0.0%	0.0%	0.0%	0.0%	0.0%	0.0%	0.0%
	Eukaryota;__Archaeplastida;__Chloroplastida;__Chlorophyta;__Mamiellophyceae;__Crustomastix	0	0.0%	0.0%	0.0%	0.0%	0.0%	0.0%	0.0%	0.0%	0.0%	0.0%	0.0%	0.0%	0.0%
	Eukaryota;__Archaeplastida;__Chloroplastida;__Chlorophyta;__Mamiellophyceae;__Dolichomastix	0	0.0%	0.0%	0.0%	0.0%	0.0%	0.0%	0.0%	0.0%	0.0%	0.0%	0.0%	0.0%	0.2%
	Eukaryota;__Archaeplastida;__Chloroplastida;__Chlorophyta;__Mamiellophyceae;__Mamiella	0	0.0%	0.0%	0.0%	0.0%	0.0%	0.0%	0.0%	0.0%	0.0%	0.0%	0.0%	0.0%	0.0%
	Eukaryota;__Archaeplastida;__Chloroplastida;__Chlorophyta;__Mamiellophyceae;__Monomastix	0	0.0%	0.0%	0.0%	0.0%	0.0%	0.0%	0.0%	0.0%	0.0%	0.0%	0.0%	0.0%	0.0%
	Eukaryota;__Archaeplastida;__Chloroplastida;__Chlorophyta;__Mamiellophyceae;__uncultured_eukaryote	0	0.0%	0.0%	0.0%	0.0%	0.0%	0.0%	0.0%	0.0%	0.0%	0.0%	0.0%	0.0%	0.0%
	Eukaryota;__Archaeplastida;__Chloroplastida;__Chlorophyta;__Nephroselmidophyceae;__Nephroselmis	0	0.0%	0.0%	0.0%	0.0%	0.0%	0.0%	0.0%	0.0%	0.0%	0.0%	0.0%	0.0%	0.0%
	Eukaryota;__Archaeplastida;__Chloroplastida;__Chlorophyta;__Trebouxiophyceae;__Asterochloris	0	0.1%	0.0%	0.0%	0.0%	0.0%	0.2%	0.0%	0.4%	0.7%	0.0%	0.0%	0.0%	0.0%
	Eukaryota;__Archaeplastida;__Chloroplastida;__Chlorophyta;__Trebouxiophyceae;__Characium	0	0.0%	0.0%	0.0%	0.0%	0.0%	0.0%	0.0%	0.0%	0.0%	0.0%	0.0%	0.0%	0.0%
	Eukaryota;__Archaeplastida;__Chloroplastida;__Chlorophyta;__Trebouxiophyceae;__Chlorella	0	0.0%	0.0%	0.0%	0.0%	0.0%	0.0%	0.0%	0.1%	0.0%	0.0%	0.0%	0.0%	0.0%
	Eukaryota;__Archaeplastida;__Chloroplastida;__Chlorophyta;__Trebouxiophyceae;__Chloroidium	0	0.0%	0.0%	0.0%	0.1%	0.0%	0.0%	0.0%	0.0%	0.0%	0.0%	0.0%	0.0%	0.0%
	Eukaryota;__Archaeplastida;__Chloroplastida;__Chlorophyta;__Trebouxiophyceae;__Coccomyxa	0	0.0%	0.0%	0.0%	0.0%	0.1%	0.0%	0.0%	0.0%	0.2%	0.0%	0.0%	0.0%	0.0%
	Eukaryota;__Archaeplastida;__Chloroplastida;__Chlorophyta;__Trebouxiophyceae;__Didymogenes	0	0.0%	0.0%	0.0%	0.0%	0.0%	0.0%	0.0%	0.0%	0.0%	0.0%	0.0%	0.0%	0.0%
	Eukaryota;__Archaeplastida;__Chloroplastida;__Chlorophyta;__Trebouxiophyceae;__Diplosphaera	0	0.0%	0.0%	0.0%	0.0%	0.0%	0.0%	0.0%	0.0%	0.0%	0.0%	0.0%	0.0%	0.0%
	Eukaryota;__Archaeplastida;__Chloroplastida;__Chlorophyta;__Trebouxiophyceae;__Elliptochloris	0	0.0%	0.0%	0.0%	0.0%	0.0%	0.0%	0.0%	0.0%	0.0%	0.0%	0.0%	0.0%	0.0%
	Eukaryota;__Archaeplastida;__Chloroplastida;__Chlorophyta;__Trebouxiophyceae;__Koliella	0	0.0%	0.0%	0.0%	0.0%	0.0%	0.0%	0.0%	0.0%	0.0%	0.0%	0.0%	0.0%	0.0%
	Eukaryota;__Archaeplastida;__Chloroplastida;__Chlorophyta;__Trebouxiophyceae;__Makinoella	0	0.0%	0.0%	0.0%	0.0%	0.0%	0.0%	0.0%	0.0%	0.0%	0.0%	0.0%	0.0%	0.0%
	Eukaryota;__Archaeplastida;__Chloroplastida;__Chlorophyta;__Trebouxiophyceae;__Meyerella	0	0.0%	0.0%	0.0%	0.0%	0.0%	0.0%	0.0%	0.0%	0.0%	0.0%	0.0%	0.0%	0.0%
	Eukaryota;__Archaeplastida;__Chloroplastida;__Chlorophyta;__Trebouxiophyceae;__Microthamnion	0	0.0%	0.0%	0.0%	0.0%	0.0%	0.0%	0.0%	0.0%	0.0%	0.0%	0.0%	0.0%	0.0%
	Eukaryota;__Archaeplastida;__Chloroplastida;__Chlorophyta;__Trebouxiophyceae;__Nannochloris	0	0.0%	0.0%	0.0%	0.0%	0.0%	0.0%	0.0%	0.0%	0.0%	0.0%	0.0%	0.0%	0.0%
	Eukaryota;__Archaeplastida;__Chloroplastida;__Chlorophyta;__Trebouxiophyceae;__Parachlorella	0	0.0%	0.0%	0.0%	0.0%	0.0%	0.0%	0.0%	0.0%	0.0%	0.0%	0.0%	0.0%	0.0%
	Eukaryota;__Archaeplastida;__Chloroplastida;__Chlorophyta;__Trebouxiophyceae;__Prasiola	0	0.0%	0.0%	0.0%	0.0%	0.0%	0.0%	0.0%	0.0%	0.0%	0.0%	0.0%	0.0%	0.0%
	Eukaryota;__Archaeplastida;__Chloroplastida;__Chlorophyta;__Trebouxiophyceae;__Prototheca	0	0.9%	0.1%	0.6%	0.3%	0.0%	6.4%	2.9%	0.5%	0.0%	0.0%	0.0%	0.1%	0.2%
	Eukaryota;__Archaeplastida;__Chloroplastida;__Chlorophyta;__Trebouxiophyceae;__Pseudochlorella	0	0.0%	0.0%	0.0%	0.0%	0.1%	0.0%	0.0%	0.0%	0.0%	0.0%	0.0%	0.0%	0.0%
	Eukaryota;__Archaeplastida;__Chloroplastida;__Chlorophyta;__Trebouxiophyceae;__Pseudomarvania	0	0.0%	0.0%	0.0%	0.0%	0.0%	0.0%	0.0%	0.0%	0.0%	0.0%	0.0%	0.0%	0.0%
	Eukaryota;__Archaeplastida;__Chloroplastida;__Chlorophyta;__Trebouxiophyceae;__Radiofilum	0	0.0%	0.0%	0.0%	0.0%	0.0%	0.1%	0.0%	0.0%	0.0%	0.0%	0.0%	0.0%	0.0%
	Eukaryota;__Archaeplastida;__Chloroplastida;__Chlorophyta;__Trebouxiophyceae;__Raphidonema	1	12.4%	7.0%	3.4%	42.7%	1.5%	0.1%	0.0%	0.1%	0.2%	0.0%	14.0%	50.9%	28.9%
	Eukaryota;__Archaeplastida;__Chloroplastida;__Chlorophyta;__Trebouxiophyceae;__Stichococcus	0	0.0%	0.0%	0.0%	0.0%	0.0%	0.0%	0.0%	0.1%	0.3%	0.0%	0.0%	0.0%	0.0%
	Eukaryota;__Archaeplastida;__Chloroplastida;__Chlorophyta;__Trebouxiophyceae;__Trebouxia	0	0.0%	0.0%	0.0%	0.0%	0.2%	0.0%	0.0%	0.0%	0.0%	0.0%	0.0%	0.0%	0.0%
	Eukaryota;__Archaeplastida;__Chloroplastida;__Chlorophyta;__Trebouxiophyceae;__uncultured_Banisveld_eukaryote	0	0.0%	0.0%	0.0%	0.1%	0.0%	0.0%	0.0%	0.0%	0.0%	0.0%	0.0%	0.0%	0.0%
	Eukaryota;__Archaeplastida;__Chloroplastida;__Chlorophyta;__Trebouxiophyceae;__uncultured_Chlorodendraceae	0	0.0%	0.0%	0.0%	0.0%	0.0%	0.0%	0.0%	0.0%	0.0%	0.0%	0.0%	0.0%	0.0%
	Eukaryota;__Archaeplastida;__Chloroplastida;__Chlorophyta;__Trebouxiophyceae;__uncultured_Chlorophyta	0	0.2%	0.0%	0.0%	0.0%	1.1%	0.0%	0.0%	0.0%	0.0%	1.1%	0.0%	0.0%	0.0%
	Eukaryota;__Archaeplastida;__Chloroplastida;__Chlorophyta;__Ulvophyceae;__Pseudendocloniopsis	0	0.0%	0.0%	0.0%	0.0%	0.0%	0.0%	0.0%	0.0%	0.0%	0.0%	0.0%	0.0%	0.0%
	Eukaryota;__Archaeplastida;__Chloroplastida;__Chlorophyta;__Ulvophyceae;__Trentepohlia	0	0.0%	0.0%	0.0%	0.0%	0.0%	0.0%	0.0%	0.0%	0.1%	0.0%	0.0%	0.0%	0.0%
	Eukaryota;__Archaeplastida;__Chloroplastida;__Chlorophyta;__Ulvophyceae;__uncultured_eukaryote	0	0.0%	0.0%	0.0%	0.0%	0.0%	0.0%	0.0%	0.0%	0.0%	0.0%	0.0%	0.0%	0.0%
	Eukaryota;__Archaeplastida;__Chloroplastida;__uncultured;__Jatropha;__Jatropha_curcas	0	0.0%	0.0%	0.0%	0.0%	0.1%	0.0%	0.0%	0.0%	0.1%	0.0%	0.0%	0.0%	0.0%
	Eukaryota;__Archaeplastida;__Chloroplastida;__uncultured;__Neosetophoma;__Neosetophoma_samarorum	0	0.0%	0.0%	0.0%	0.0%	0.0%	0.0%	0.0%	0.0%	0.0%	0.0%	0.0%	0.0%	0.0%

	Eukaryota;__Archaeplastida;__Chloroplastida;__uncultured;__uncultured_Ascomycota;Other	0	0.0%	0.0%	0.0%	0.0%	0.0%	0.0%	0.0%	0.0%	0.0%	0.0%	0.0%	0.0%	0.0%
	Eukaryota;__Archaeplastida;__Rhodophyceae;__Compsopogonales;__Compsopogon;__Compsopogonopsis	0	0.0%	0.0%	0.0%	0.0%	0.0%	0.0%	0.0%	0.0%	0.0%	0.0%	0.0%	0.0%	0.0%
	Eukaryota;__Centrohelida;__Heterophryidae;__Heterophrys;__Heterophrys_sp._Oxford1;Other	0	0.0%	0.0%	0.0%	0.0%	0.0%	0.0%	0.0%	0.0%	0.0%	0.0%	0.0%	0.0%	0.0%
	Eukaryota;__Kathablepharidae;__Roombia;__Roombia_truncata;Other;Other	0	0.0%	0.0%	0.0%	0.0%	0.0%	0.0%	0.0%	0.0%	0.0%	0.0%	0.0%	0.0%	0.0%
	Eukaryota;__Opisthokonta;__Fungi;__Ascomycota;__Pezizomycotina;__Dothideomycetes	0	0.8%	0.0%	0.2%	0.1%	0.9%	3.8%	0.2%	1.2%	2.2%	1.1%	0.0%	0.0%	0.0%
	Eukaryota;__Opisthokonta;__Fungi;__Ascomycota;__Pezizomycotina;__Eurotiomycetes	0	0.1%	0.0%	0.0%	0.0%	0.1%	0.1%	0.0%	0.0%	0.1%	0.0%	0.1%	0.0%	0.4%
	Eukaryota;__Opisthokonta;__Fungi;__Ascomycota;__Pezizomycotina;__Incertae_Sedis	0	0.0%	0.0%	0.0%	0.0%	0.0%	0.1%	0.0%	0.0%	0.0%	0.0%	0.0%	0.0%	0.0%
	Eukaryota;__Opisthokonta;__Fungi;__Ascomycota;__Pezizomycotina;__Lecanoromycetes	0	0.4%	0.0%	0.0%	0.0%	2.7%	0.2%	0.0%	0.4%	0.9%	1.1%	0.0%	0.0%	0.0%
	Eukaryota;__Opisthokonta;__Fungi;__Ascomycota;__Pezizomycotina;__Leotiomycetes	0	1.0%	0.0%	1.2%	0.0%	1.1%	3.0%	0.0%	2.0%	1.3%	3.2%	0.0%	0.0%	0.1%
	Eukaryota;__Opisthokonta;__Fungi;__Ascomycota;__Pezizomycotina;__Lichinomycetes	0	0.0%	0.0%	0.0%	0.0%	0.1%	0.0%	0.0%	0.0%	0.0%	0.0%	0.0%	0.0%	0.0%
	Eukaryota;__Opisthokonta;__Fungi;__Ascomycota;__Pezizomycotina;__Orbiliomycetes	0	0.0%	0.0%	0.0%	0.0%	0.0%	0.0%	0.0%	0.0%	0.0%	0.0%	0.0%	0.0%	0.0%
	Eukaryota;__Opisthokonta;__Fungi;__Ascomycota;__Pezizomycotina;__Pezizomycetes	0	0.0%	0.0%	0.0%	0.0%	0.0%	0.0%	0.0%	0.0%	0.0%	0.0%	0.0%	0.0%	0.0%
	Eukaryota;__Opisthokonta;__Fungi;__Ascomycota;__Pezizomycotina;__Sordariomycetes	0	0.0%	0.0%	0.0%	0.0%	0.1%	0.1%	0.0%	0.1%	0.0%	0.0%	0.0%	0.0%	0.0%
	Eukaryota;__Opisthokonta;__Fungi;__Ascomycota;__Pezizomycotina;__uncultured_soil_fungus	0	0.0%	0.0%	0.0%	0.0%	0.0%	0.0%	0.0%	0.0%	0.0%	0.0%	0.0%	0.0%	0.0%
	Eukaryota;__Opisthokonta;__Fungi;__Ascomycota;__Taphrinomycotina;__Taphrinomycetes	0	0.0%	0.0%	0.0%	0.0%	0.0%	0.0%	0.0%	0.1%	0.0%	0.0%	0.0%	0.0%	0.0%
	Eukaryota;__Opisthokonta;__Fungi;__Basal_fungi;__Blastocladiomycota;__Blastocladiales	0	0.0%	0.0%	0.0%	0.0%	0.0%	0.0%	0.0%	0.0%	0.0%	0.0%	0.0%	0.0%	0.0%
	Eukaryota;__Opisthokonta;__Fungi;__Basal_fungi;__Blastocladiomycota;__Hyaloraphidium	0	0.0%	0.0%	0.0%	0.1%	0.0%	0.0%	0.0%	0.0%	0.0%	0.0%	0.0%	0.1%	0.0%
	Eukaryota;__Opisthokonta;__Fungi;__Basal_fungi;__Blastocladiomycota;__uncultured_eukaryote	0	0.0%	0.0%	0.0%	0.0%	0.0%	0.0%	0.0%	0.0%	0.0%	0.0%	0.0%	0.0%	0.0%
	Eukaryota;__Opisthokonta;__Fungi;__Basal_fungi;__Blastocladiomycota;__uncultured_fungus	0	0.0%	0.0%	0.4%	0.0%	0.0%	0.0%	0.0%	0.0%	0.0%	0.0%	0.0%	0.0%	0.0%
	Eukaryota;__Opisthokonta;__Fungi;__Basal_fungi;__Chytridiomycota;__Blyttiomyces	0	0.0%	0.0%	0.0%	0.0%	0.0%	0.0%	0.0%	0.0%	0.0%	0.0%	0.0%	0.0%	0.1%
	Eukaryota;__Opisthokonta;__Fungi;__Basal_fungi;__Chytridiomycota;__Chytridiales	0	0.0%	0.0%	0.0%	0.0%	0.0%	0.0%	0.0%	0.0%	0.0%	0.0%	0.0%	0.0%	0.0%
	Eukaryota;__Opisthokonta;__Fungi;__Basal_fungi;__Chytridiomycota;__Chytriomyces	0	0.0%	0.0%	0.0%	0.0%	0.0%	0.0%	0.0%	0.0%	0.0%	0.0%	0.0%	0.0%	0.0%
	Eukaryota;__Opisthokonta;__Fungi;__Basal_fungi;__Chytridiomycota;__Cyllamyces	0	0.0%	0.0%	0.0%	0.0%	0.0%	0.0%	0.0%	0.0%	0.0%	0.0%	0.0%	0.0%	0.0%
	Eukaryota;__Opisthokonta;__Fungi;__Basal_fungi;__Chytridiomycota;__Gaertneriomyces	0	0.0%	0.0%	0.0%	0.0%	0.0%	0.0%	0.0%	0.0%	0.0%	0.0%	0.0%	0.0%	0.0%
	Eukaryota;__Opisthokonta;__Fungi;__Basal_fungi;__Chytridiomycota;__Neocallimastix	0	0.0%	0.0%	0.0%	0.0%	0.0%	0.0%	0.0%	0.0%	0.0%	0.0%	0.0%	0.0%	0.0%
	Eukaryota;__Opisthokonta;__Fungi;__Basal_fungi;__Chytridiomycota;__Rhizophlyctis	0	0.0%	0.0%	0.0%	0.0%	0.0%	0.0%	0.0%	0.0%	0.0%	0.0%	0.0%	0.0%	0.0%
	Eukaryota;__Opisthokonta;__Fungi;__Basal_fungi;__Chytridiomycota;__Spizellomyces	0	0.0%	0.0%	0.0%	0.0%	0.0%	0.0%	0.0%	0.0%	0.0%	0.0%	0.0%	0.0%	0.0%
	Eukaryota;__Opisthokonta;__Fungi;__Basal_fungi;__Chytridiomycota;__uncultured_Chytridiomycota	0	2.6%	4.5%	1.6%	0.2%	0.0%	0.4%	0.0%	0.0%	0.0%	0.0%	10.7%	10.7%	3.6%
	Eukaryota;__Opisthokonta;__Fungi;__Basal_fungi;__Chytridiomycota;__uncultured_Rhizophydiaceae	0	0.0%	0.0%	0.0%	0.0%	0.0%	0.0%	0.0%	0.0%	0.0%	0.0%	0.0%	0.0%	0.0%
	Eukaryota;__Opisthokonta;__Fungi;__Basal_fungi;__Chytridiomycota;__uncultured_eukaryote	0	0.1%	0.1%	1.2%	0.1%	0.0%	0.0%	0.0%	0.0%	0.0%	0.0%	0.0%	0.0%	0.0%
	Eukaryota;__Opisthokonta;__Fungi;__Basal_fungi;__Chytridiomycota;__uncultured_eukaryotic_picoplankton	0	0.0%	0.0%	0.2%	0.0%	0.0%	0.0%	0.0%	0.0%	0.0%	0.0%	0.0%	0.0%	0.0%
	Eukaryota;__Opisthokonta;__Fungi;__Basal_fungi;__Chytridiomycota;__uncultured_fungus	0	0.0%	0.0%	0.0%	0.0%	0.0%	0.0%	0.0%	0.0%	0.0%	0.0%	0.0%	0.0%	0.0%
	Eukaryota;__Opisthokonta;__Fungi;__Basal_fungi;__Chytridiomycota;__uncultured_microeukaryote	0	0.0%	0.0%	0.0%	0.0%	0.0%	0.0%	0.0%	0.0%	0.0%	0.0%	0.0%	0.0%	0.0%
	Eukaryota;__Opisthokonta;__Fungi;__Basal_fungi;__Chytridiomycota;__uncultured_soil_fungus	0	0.0%	0.0%	0.0%	0.0%	0.0%	0.0%	0.0%	0.0%	0.0%	0.0%	0.0%	0.0%	0.0%
	Eukaryota;__Opisthokonta;__Fungi;__Basal_fungi;__Entomophthoromycotina;__Basidiobolus	0	0.0%	0.0%	0.0%	0.0%	0.0%	0.0%	0.0%	0.0%	0.0%	0.0%	0.0%	0.0%	0.0%
	Eukaryota;__Opisthokonta;__Fungi;__Basal_fungi;__Glomeromycota;__Glomeromycota_sp._MIB_8442	0	0.0%	0.0%	0.0%	0.0%	0.0%	0.0%	0.0%	0.0%	0.0%	0.0%	0.0%	0.0%	0.0%
	Eukaryota;__Opisthokonta;__Fungi;__Basal_fungi;__Mucoromycotina;__Endogone	0	0.0%	0.0%	0.0%	0.0%	0.0%	0.0%	0.0%	0.0%	0.0%	0.0%	0.0%	0.0%	0.0%
	Eukaryota;__Opisthokonta;__Fungi;__Basal_fungi;__Mucoromycotina;__Mucoromycotina_sp._MIB_8359	0	0.0%	0.0%	0.0%	0.0%	0.0%	0.0%	0.0%	0.0%	0.0%	0.0%	0.0%	0.0%	0.0%
	Eukaryota;__Opisthokonta;__Fungi;__Basal_fungi;__Mucoromycotina;__uncultured_soil_fungus	0	0.0%	0.0%	0.4%	0.0%	0.1%	0.0%	0.0%	0.0%	0.0%	0.0%	0.0%	0.0%	0.0%
	Eukaryota;__Opisthokonta;__Fungi;__Basal_fungi;__uncultured;__Cercomonadida	0	0.0%	0.0%	0.0%	0.0%	0.0%	0.0%	0.0%	0.0%	0.0%	0.0%	0.0%	0.0%	0.0%
	Eukaryota;__Opisthokonta;__Fungi;__Basal_fungi;__uncultured;__uncultured_Eimeriidae	0	0.7%	3.3%	0.6%	4.2%	0.0%	0.0%	0.0%	0.0%	0.0%	0.0%	0.1%	0.1%	0.1%
	Eukaryota;__Opisthokonta;__Fungi;__Basal_fungi;__uncultured_Chytridiomycota;Other	0	0.0%	0.0%	0.0%	0.0%	0.0%	0.0%	0.0%	0.0%	0.0%	0.0%	0.0%	0.0%	0.0%
	Eukaryota;__Opisthokonta;__Fungi;__Basal_fungi;__uncultured_eukaryote;Other	0	0.0%	0.0%	0.0%	0.0%	0.0%	0.0%	0.0%	0.0%	0.0%	0.0%	0.0%	0.0%	0.0%
	Eukaryota;__Opisthokonta;__Fungi;__Basidiomycota;__Agaricomycotina;__Agaricomycetes	0	0.0%	0.0%	0.0%	0.3%	0.0%	0.0%	0.0%	0.0%	0.0%	0.0%	0.0%	0.0%	0.0%
	Eukaryota;__Opisthokonta;__Fungi;__Basidiomycota;__Agaricomycotina;__Tremellomycetes	0	0.0%	0.0%	0.0%	0.0%	0.0%	0.0%	0.0%	0.0%	0.0%	0.0%	0.0%	0.0%	0.0%
	Eukaryota;__Opisthokonta;__Fungi;__Basidiomycota;__Agaricomycotina;__uncultured_Auriculariaceae	0	0.0%	0.0%	0.0%	0.0%	0.0%	0.0%	0.0%	0.0%	0.0%	0.0%	0.0%	0.0%	0.0%
	Eukaryota;__Opisthokonta;__Fungi;__Basidiomycota;__Pucciniomycotina;__Microbotryomycetes	4	37.1%	15.3%	22.8%	12.9%	37.5%	70.5%	92.2%	66.6%	60.4%	64.9%	0.1%	0.7%	0.8%
	Eukaryota;__Opisthokonta;__Fungi;__Basidiomycota;__Pucciniomycotina;__Pucciniomycetes	0	0.0%	0.0%	0.0%	0.0%	0.0%	0.0%	0.0%	0.0%	0.0%	0.0%	0.0%	0.0%	0.0%
	Eukaryota;__Opisthokonta;__Fungi;__LKM11;__uncultured_fungus;Other	0	0.0%	0.0%	0.0%	0.0%	0.0%	0.0%	0.1%	0.0%	0.0%	0.0%	0.0%	0.0%	0.0%
	Eukaryota;__Opisthokonta;__Fungi;__LKM15;__uncultured_Clathrinidae_sp.;Other	0	0.8%	0.0%	0.4%	0.0%	0.0%	0.0%	0.0%	0.0%	0.0%	0.0%	2.1%	0.8%	6.4%
	Eukaryota;__Opisthokonta;__Fungi;__Nucletmycea;__Fonticula;__uncultured_eukaryote	0	0.0%	0.0%	0.0%	0.0%	0.0%	0.0%	0.0%	0.0%	0.0%	0.0%	0.0%	0.0%	0.0%
	Eukaryota;__Opisthokonta;__Holozoa;__Choanomonada;__Acanthoecida;__Diaphanoeca	0	0.0%	0.0%	0.0%	0.0%	0.0%	0.0%	0.0%	0.0%	0.0%	0.0%	0.0%	0.0%	0.0%
	Eukaryota;__Opisthokonta;__Holozoa;__Choanomonada;__Acanthoecida;__FV23-1A4	0	0.0%	0.0%	0.0%	0.0%	0.0%	0.0%	0.0%	0.0%	0.0%	0.0%	0.0%	0.0%	0.0%
	Eukaryota;__Opisthokonta;__Holozoa;__Choanomonada;__Craspedida;__OLI11041	0	0.0%	0.0%	0.0%	0.0%	0.0%	0.0%	0.0%	0.0%	0.0%	0.0%	0.0%	0.0%	0.0%
	Eukaryota;__Opisthokonta;__Metazoa;__Annelida;__Family_Incertae_Sedis;__Notomastus	0	0.0%	0.0%	0.0%	0.0%	0.0%	0.0%	0.0%	0.0%	0.0%	0.0%	0.0%	0.0%	0.0%
	Eukaryota;__Opisthokonta;__Metazoa;__Arthropoda;__Chelicerata;__Arachnida	0	0.0%	0.0%	0.0%	0.0%	0.0%	0.0%	0.0%	0.0%	0.0%	0.0%	0.0%	0.0%	0.0%
	Eukaryota;__Opisthokonta;__Metazoa;__Arthropoda;__Crustacea;__Malacostraca	0	0.0%	0.0%	0.0%	0.0%	0.0%	0.0%	0.0%	0.0%	0.0%	0.0%	0.0%	0.0%	0.0%
	Eukaryota;__Opisthokonta;__Metazoa;__Arthropoda;__Hexapoda;__Collembola	0	0.0%	0.0%	0.0%	0.0%	0.0%	0.0%	0.0%	0.0%	0.0%	0.0%	0.0%	0.0%	0.0%
	Eukaryota;__Opisthokonta;__Metazoa;__Cnidaria;__Anthozoa;__Antipathes	0	0.0%	0.0%	0.0%	0.0%	0.0%	0.0%	0.0%	0.0%	0.0%	0.0%	0.0%	0.0%	0.0%
	Eukaryota;__Opisthokonta;__Metazoa;__Cnidaria;__Anthozoa;__Flosmaris	0	0.0%	0.0%	0.0%	0.0%	0.0%	0.0%	0.0%	0.0%	0.0%	0.0%	0.0%	0.0%	0.0%
	Eukaryota;__Opisthokonta;__Metazoa;__Cnidaria;__Anthozoa;__Umbellula	0	0.0%	0.0%	0.0%	0.0%	0.0%	0.0%	0.0%	0.0%	0.0%	0.0%	0.0%	0.0%	0.0%
	Eukaryota;__Opisthokonta;__Metazoa;__Gastrotricha;__Thaumastodermatidae;__Diplodasys	0	0.0%	0.0%	0.0%	0.0%	0.0%	0.0%	0.0%	0.0%	0.0%	0.0%	0.0%	0.0%	0.0%
	Eukaryota;__Opisthokonta;__Metazoa;__Nematoda;__Chromadorea;__Axonolaimidae	0	0.0%	0.0%	0.0%	0.0%	0.0%	0.0%	0.0%	0.0%	0.0%	0.0%	0.0%	0.0%	0.0%
	Eukaryota;__Opisthokonta;__Metazoa;__Platyhelminthes;__Turbellaria;__Rhabdocoela	0	0.0%	0.0%	0.0%	0.0%	0.0%	0.0%	0.0%	0.0%	0.0%	0.0%	0.0%	0.0%	0.0%
	Eukaryota;__Opisthokonta;__Metazoa;__Porifera;__Calcarea;__Clathrina	0	0.0%	0.0%	0.0%	0.0%	0.0%	0.0%	0.0%	0.0%	0.0%	0.0%	0.0%	0.0%	0.0%
	Eukaryota;__Opisthokonta;__Metazoa;__Porifera;__Hexactinellida;__Sympagella	0	0.0%	0.0%	0.0%	0.0%	0.0%	0.0%	0.0%	0.0%	0.0%	0.0%	0.0%	0.0%	0.0%
	Eukaryota;__Opisthokonta;__Metazoa;__Rotifera;__Family_Incertae_Sedis;__Encentrum	0	0.0%	0.0%	0.0%	0.0%	0.0%	0.0%	0.0%	0.0%	0.0%	0.0%	0.0%	0.0%	0.0%
	Eukaryota;__Opisthokonta;__Metazoa;__Rotifera;__Family_Incertae_Sedis;__uncultured_metazoan	0	0.0%	0.0%	0.0%	0.0%	0.0%	0.0%	0.0%	0.0%	0.0%	0.0%	0.0%	0.0%	0.0%
	Eukaryota;__Opisthokonta;__Metazoa;__Rotifera;__Philodinidae;__Adineta	0	0.0%	0.0%	0.0%	0.0%	0.0%	0.0%	0.0%	0.0%	0.0%	0.0%	0.0%	0.0%	0.0%
	Eukaryota;__Opisthokonta;__Metazoa;__Rotifera;__Philodinidae;__Rotaria	0	0.0%	0.0%	0.0%	0.0%	0.0%	0.0%	0.0%	0.0%	0.0%	0.0%	0.0%	0.0%	0.0%
	Eukaryota;__Opisthokonta;__Metazoa;__Rotifera;__Philodinidae;__uncultured_bdelloid_rotifer	0	0.0%	0.0%	0.0%	0.0%	0.0%	0.0%	0.0%	0.0%	0.0%	0.0%	0.0%	0.0%	0.0%
	Eukaryota;__Opisthokonta;__Metazoa;__Tardigrada;__Hypsibiidae;__Acutuncus	0	0.0%	0.0%	0.0%	0.0%	0.0%	0.0%	0.0%	0.0%	0.0%	0.0%	0.0%	0.0%	0.0%
	Eukaryota;__Opisthokonta;__Metazoa;__Tardigrada;__Hypsibiidae;__Calohypsibius	0	0.0%	0.0%	0.0%	0.0%	0.0%	0.0%	0.0%	0.0%	0.0%	0.0%	0.0%	0.0%	0.0%
	Eukaryota;__Opisthokonta;__Metazoa;__Tardigrada;__Hypsibiidae;__Eremobiotus	0	0.0%	0.0%	0.0%	0.0%	0.0%	0.0%	0.0%	0.0%	0.0%	0.0%	0.0%	0.0%	0.0%
	Eukaryota;__Opisthokonta;__Metazoa;__Tardigrada;__Hypsibiidae;__Tardigrada	0	0.0%	0.0%	0.0%	0.0%	0.0%	0.0%	0.0%	0.0%	0.0%	0.0%	0.0%	0.0%	0.0%
	Eukaryota;__Opisthokonta;__uncultured;__uncultured_Sarcosomataceae;Other;Other	0	0.0%	0.0%	0.0%	0.0%	0.0%	0.0%	0.0%	0.0%	0.0%	0.0%	0.0%	0.0%	0.0%
	Eukaryota;__RT5iin25;__uncultured_Eimeriidae;Other;Other;Other	0	0.0%	0.0%	0.0%	0.0%	0.0%	0.0%	0.0%	0.0%	0.0%	0.0%	0.0%	0.0%	0.0%
	Eukaryota;__RT5iin25;__uncultured_freshwater_eukaryote;Other;Other;Other	0	0.0%	0.0%	0.0%	0.0%	0.0%	0.0%	0.0%	0.0%	0.0%	0.0%	0.0%	0.0%	0.0%
	Eukaryota;__SAR;__Alveolata;__Apicomplexa;__Conoidasida;__Coccidia	0	0.0%	0.0%	0.0%	0.0%	0.0%	0.0%	0.0%	0.0%	0.0%	0.0%	0.0%	0.0%	0.0%
	Eukaryota;__SAR;__Alveolata;__Apicomplexa;__Conoidasida;__Gregarinasina	0	0.0%	0.0%	0.0%	0.0%	0.0%	0.0%	0.0%	0.0%	0.0%	0.0%	0.0%	0.0%	0.0%
	Eukaryota;__SAR;__Alveolata;__Ciliophora;__Intramacronucleata;__Conthreep	0	2.0%	0.1%	3.4%	2.0%	0.0%	0.0%	0.0%	0.1%	0.0%	0.0%	13.7%	1.4%	3.2%
	Eukaryota;__SAR;__Alveolata;__Ciliophora;__Intramacronucleata;__Litostomatea	0	0.3%	0.3%	1.6%	0.6%	0.3%	0.1%	0.1%	0.0%	0.0%	0.0%	0.1%	0.7%	0.0%
	Eukaryota;__SAR;__Alveolata;__Ciliophora;__Intramacronucleata;__Spirotrichea	0	0.3%	0.2%	1.4%	0.6%	0.0%	0.0%	0.0%	0.7%	0.2%	0.0%	0.5%	0.4%	0.1%
	Eukaryota;__SAR;__Alveolata;__Dinoflagellata;__Dinophyceae;__Gymnodiniphycidae	0	0.0%	0.0%	0.0%	0.0%	0.0%	0.0%	0.0%	0.0%	0.0%	0.0%	0.0%	0.0%	0.0%
	Eukaryota;__SAR;__Alveolata;__Dinoflagellata;__Dinophyceae;__Peridiniphycidae	0	0.0%	0.0%	0.0%	0.0%	0.0%	0.0%	0.0%	0.0%	0.0%	0.0%	0.0%	0.0%	0.0%
	Eukaryota;__SAR;__Alveolata;__NIF-4C10;__uncultured_marine_alveolate;Other	0	0.0%	0.0%	0.0%	0.0%	0.0%	0.0%	0.0%	0.0%	0.0%	0.0%	0.0%	0.0%	0.0%
	Eukaryota;__SAR;__Alveolata;__Protalveolata;__Perkinsidae;__A31	0	0.0%	0.0%	0.0%	0.3%	0.0%	0.0%	0.0%	0.0%	0.0%	0.0%	0.0%	0.0%	0.0%
	Eukaryota;__SAR;__Rhizaria;__Cercozoa;__Athalamea;__Athalamea_environmental_sample	0	0.4%	1.5%	0.0%	3.5%	0.0%	0.0%	0.0%	0.0%	0.0%	0.0%	0.0%	0.0%	0.0%
	Eukaryota;__SAR;__Rhizaria;__Cercozoa;__Cercomonadidae;__Cavernomonas	0	0.0%	0.0%	0.0%	0.0%	0.0%	0.0%	0.0%	0.0%	0.0%	0.0%	0.0%	0.0%	0.0%
	Eukaryota;__SAR;__Rhizaria;__Cercozoa;__Cercomonadidae;__Cercomonas	0	0.0%	0.0%	0.0%	0.1%	0.0%	0.0%	0.0%	0.0%	0.0%	0.0%	0.0%	0.0%	0.0%
	Eukaryota;__SAR;__Rhizaria;__Cercozoa;__Endomyxa;__Novel_Clade_10	0	0.0%	0.0%	0.0%	0.0%	0.0%	0.0%	0.0%	0.0%	0.0%	0.0%	0.0%	0.0%	0.0%
	Eukaryota;__SAR;__Rhizaria;__Cercozoa;__Glissomonadida;__Allantion	0	0.0%	0.0%	0.0%	0.0%	0.0%	0.0%	0.0%	0.0%	0.0%	0.0%	0.0%	0.0%	0.0%
	Eukaryota;__SAR;__Rhizaria;__Cercozoa;__Glissomonadida;__Amb-18S-901	0	0.0%	0.0%	0.0%	0.0%	0.0%	0.0%	0.0%	0.0%	0.0%	0.0%	0.0%	0.0%	0.0%
	Eukaryota;__SAR;__Rhizaria;__Cercozoa;__Glissomonadida;__Bodomorpha	0	0.0%	0.1%	0.0%	0.2%	0.0%	0.0%	0.0%	0.0%	0.0%	0.0%	0.0%	0.0%	0.0%
	Eukaryota;__SAR;__Rhizaria;__Cercozoa;__Glissomonadida;__Heteromita	1	10.1%	28.0%	40.1%	24.5%	1.9%	1.6%	1.8%	12.6%	2.7%	5.3%	0.9%	1.4%	0.5%
	Eukaryota;__SAR;__Rhizaria;__Cercozoa;__Glissomonadida;__uncultured	0	0.0%	0.0%	0.0%	0.0%	0.0%	0.0%	0.0%	0.0%	0.0%	0.0%	0.0%	0.0%	0.0%
	Eukaryota;__SAR;__Rhizaria;__Cercozoa;__Glissomonadida;__uncultured_cercozoan	0	0.0%	0.0%	0.0%	0.0%	0.0%	0.0%	0.0%	0.0%	0.0%	0.0%	0.0%	0.0%	0.0%
	Eukaryota;__SAR;__Rhizaria;__Cercozoa;__Glissomonadida;__uncultured_eukaryote	0	0.0%	0.0%	0.0%	0.0%	0.0%	0.0%	0.0%	0.0%	0.0%	0.0%	0.0%	0.0%	0.0%
	Eukaryota;__SAR;__Rhizaria;__Cercozoa;__Incertae_Sedis;__Gymnophrys	0	0.0%	0.0%	0.0%	0.0%	0.0%	0.0%	0.0%	0.0%	0.0%	0.0%	0.0%	0.0%	0.0%
	Eukaryota;__SAR;__Rhizaria;__Cercozoa;__Metromonadea;__Metopion	0	0.0%	0.0%	0.0%	0.0%	0.0%	0.0%	0.0%	0.0%	0.0%	0.0%	0.0%	0.0%	0.0%
	Eukaryota;__SAR;__Rhizaria;__Cercozoa;__Novel_Clade_12;__uncultured_freshwater_cercozoan	0	0.0%	0.0%	0.0%	0.0%	0.0%	0.0%	0.0%	0.0%	0.0%	0.0%	0.0%	0.0%	0.0%
	Eukaryota;__SAR;__Rhizaria;__Cercozoa;__PP1-8;__uncultured_Heteromitidae	0	0.0%	0.0%	0.0%	0.0%	0.0%	0.0%	0.0%	0.0%	0.0%	0.0%	0.0%	0.0%	0.0%
	Eukaryota;__SAR;__Rhizaria;__Cercozoa;__PP1-8;__uncultured_cercozoan	0	0.0%	0.0%	0.0%	0.0%	0.0%	0.0%	0.0%	0.0%	0.0%	0.0%	0.0%	0.0%	0.0%
	Eukaryota;__SAR;__Rhizaria;__Cercozoa;__PP1-8;__uncultured_eukaryote	0	0.0%	0.0%	0.0%	0.0%	0.0%	0.0%	0.0%	0.0%	0.0%	0.0%	0.0%	0.0%	0.0%
	Eukaryota;__SAR;__Rhizaria;__Cercozoa;__Pansomonadida;__Aurigamonas	0	0.0%	0.0%	0.0%	0.1%	0.0%	0.0%	0.0%	0.0%	0.0%	0.0%	0.0%	0.0%	0.0%
	Eukaryota;__SAR;__Rhizaria;__Cercozoa;__Silicofilosea;__Euglyphida	0	0.0%	0.0%	0.0%	0.0%	0.0%	0.0%	0.0%	0.0%	0.0%	0.0%	0.0%	0.0%	0.0%
	Eukaryota;__SAR;__Rhizaria;__Cercozoa;__Silicofilosea;__Marimonadida	0	0.0%	0.0%	0.0%	0.0%	0.0%	0.0%	0.0%	0.0%	0.0%	0.0%	0.0%	0.0%	0.0%
	Eukaryota;__SAR;__Rhizaria;__Cercozoa;__Silicofilosea;__Spongomonadida	0	0.0%	0.0%	0.0%	0.0%	0.0%	0.0%	0.0%	0.0%	0.0%	0.0%	0.0%	0.0%	0.0%
	Eukaryota;__SAR;__Rhizaria;__Cercozoa;__Silicofilosea;__Thaumatomonadida	0	0.0%	0.0%	0.2%	0.0%	0.0%	0.0%	0.0%	0.1%	0.0%	0.0%	0.0%	0.0%	0.0%
	Eukaryota;__SAR;__Rhizaria;__Cercozoa;__Thecofilosea;__Amb-18S-431	0	0.0%	0.0%	0.0%	0.0%	0.0%	0.0%	0.0%	0.0%	0.0%	0.0%	0.0%	0.0%	0.0%
	Eukaryota;__SAR;__Rhizaria;__Cercozoa;__Thecofilosea;__BOLA322	0	0.0%	0.0%	0.0%	0.0%	0.0%	0.0%	0.0%	0.0%	0.0%	0.0%	0.0%	0.0%	0.0%
	Eukaryota;__SAR;__Rhizaria;__Cercozoa;__Thecofilosea;__Cryomonadida	0	0.0%	0.0%	0.0%	0.0%	0.0%	0.0%	0.1%	0.0%	0.0%	0.0%	0.0%	0.0%	0.0%
	Eukaryota;__SAR;__Rhizaria;__Cercozoa;__Thecofilosea;__E-A1	0	0.0%	0.0%	0.2%	0.1%	0.0%	0.0%	0.0%	0.0%	0.0%	0.0%	0.0%	0.0%	0.0%
	Eukaryota;__SAR;__Rhizaria;__Cercozoa;__Thecofilosea;__Ebriacea	0	0.0%	0.0%	0.0%	0.0%	0.0%	0.0%	0.0%	0.0%	0.0%	0.0%	0.0%	0.0%	0.0%
	Eukaryota;__SAR;__Rhizaria;__Cercozoa;__Thecofilosea;__NIF-3A7	0	0.0%	0.0%	0.0%	0.0%	0.0%	0.0%	0.0%	0.0%	0.0%	0.0%	0.0%	0.0%	0.0%
	Eukaryota;__SAR;__Rhizaria;__Cercozoa;__Thecofilosea;__Phaeodarea	0	0.0%	0.0%	0.0%	0.2%	0.0%	0.0%	0.0%	0.0%	0.0%	0.0%	0.0%	0.0%	0.0%
	Eukaryota;__SAR;__Rhizaria;__Cercozoa;__Thecofilosea;__WHOI-LI1-14	0	0.0%	0.0%	0.0%	0.0%	0.0%	0.0%	0.0%	0.0%	0.0%	0.0%	0.0%	0.0%	0.0%

	Eukaryota;__SAR;__Rhizaria;__Cercozoa;__Thecofilosea;__uncultured	0	0.3%	0.6%	1.8%	0.7%	0.0%	0.1%	0.0%	0.0%	0.0%	0.0%	0.0%	0.0%	0.1%
	Eukaryota;__SAR;__Rhizaria;__Cercozoa;__uncultured;__Cercozoa	0	0.0%	0.0%	0.0%	0.1%	0.0%	0.0%	0.0%	0.0%	0.0%	0.0%	0.0%	0.0%	0.0%
	Eukaryota;__SAR;__Rhizaria;__Cercozoa;__uncultured;__uncultured_Eimeriidae	0	0.0%	0.0%	0.0%	0.0%	0.0%	0.0%	0.0%	0.0%	0.0%	0.0%	0.0%	0.0%	0.0%
	Eukaryota;__SAR;__Rhizaria;__Cercozoa;__uncultured;__uncultured_cercozoan	0	0.0%	0.0%	0.0%	0.0%	0.0%	0.0%	0.0%	0.0%	0.0%	0.0%	0.0%	0.0%	0.0%
	Eukaryota;__SAR;__Rhizaria;__Cercozoa;__uncultured;__uncultured_eukaryote	0	0.0%	0.0%	0.0%	0.0%	0.0%	0.0%	0.0%	0.0%	0.0%	0.0%	0.0%	0.0%	0.0%
	Eukaryota;__SAR;__Rhizaria;__Cercozoa;__uncultured;__uncultured_eukaryotic_picoplankton	0	0.0%	0.0%	0.0%	0.0%	0.0%	0.1%	0.0%	0.0%	0.0%	0.0%	0.0%	0.0%	0.0%
	Eukaryota;__SAR;__Stramenopiles;__Bicosoecida;__P34.6;__Bicosoecida	0	0.0%	0.0%	0.0%	0.0%	0.0%	0.0%	0.0%	0.0%	0.0%	0.0%	0.0%	0.0%	0.0%
	Eukaryota;__SAR;__Stramenopiles;__Bicosoecida;__Siluaniidae;__PSA11SP2005	0	0.0%	0.0%	0.0%	0.0%	0.0%	0.0%	0.0%	0.0%	0.0%	0.0%	0.0%	0.0%	0.0%
	Eukaryota;__SAR;__Stramenopiles;__CCI40;__uncultured_marine_eukaryote;Other	0	0.0%	0.0%	0.0%	0.0%	0.0%	0.0%	0.0%	0.0%	0.0%	0.0%	0.0%	0.3%	0.0%
	Eukaryota;__SAR;__Stramenopiles;__Chrysophyceae;__CCMP1899;__Chrysophyceae_sp._I76	0	0.0%	0.0%	0.0%	0.0%	0.0%	0.0%	0.0%	0.0%	0.0%	0.0%	0.0%	0.0%	0.0%
	Eukaryota;__SAR;__Stramenopiles;__Chrysophyceae;__CCMP1899;__Ochromonas	0	0.0%	0.0%	0.0%	0.0%	0.0%	0.0%	0.0%	0.0%	0.0%	0.0%	0.0%	0.0%	0.0%
	Eukaryota;__SAR;__Stramenopiles;__Chrysophyceae;__CCMP1899;__uncultured_marine_stramenopile	0	0.0%	0.0%	0.0%	0.0%	0.0%	0.0%	0.0%	0.0%	0.0%	0.0%	0.0%	0.0%	0.0%
	Eukaryota;__SAR;__Stramenopiles;__Chrysophyceae;__Chromulinales;__Chromulina	0	0.0%	0.0%	0.0%	0.0%	0.0%	0.0%	0.0%	0.0%	0.0%	0.0%	0.0%	0.0%	0.0%
	Eukaryota;__SAR;__Stramenopiles;__Chrysophyceae;__Chromulinales;__Chrysamoeba	0	0.0%	0.0%	0.4%	0.1%	0.0%	0.0%	0.0%	0.0%	0.0%	0.0%	0.0%	0.0%	0.0%
	Eukaryota;__SAR;__Stramenopiles;__Chrysophyceae;__Chromulinales;__Chrysochaete	0	0.0%	0.0%	0.0%	0.0%	0.0%	0.0%	0.0%	0.0%	0.0%	0.0%	0.0%	0.0%	0.0%
	Eukaryota;__SAR;__Stramenopiles;__Chrysophyceae;__Chromulinales;__LG31-02	0	0.0%	0.0%	0.0%	0.0%	0.0%	0.0%	0.0%	0.0%	0.0%	0.0%	0.0%	0.0%	0.0%
	Eukaryota;__SAR;__Stramenopiles;__Chrysophyceae;__Chromulinales;__Spumella	0	0.0%	0.0%	0.0%	0.0%	0.0%	0.0%	0.0%	0.0%	0.0%	0.0%	0.0%	0.0%	0.0%
	Eukaryota;__SAR;__Stramenopiles;__Chrysophyceae;__Chrysocapsales;__Chrysocapsa	0	0.0%	0.0%	0.0%	0.0%	0.0%	0.0%	0.0%	0.0%	0.0%	0.0%	0.0%	0.0%	0.0%
	Eukaryota;__SAR;__Stramenopiles;__Chrysophyceae;__Chrysocapsales;__Hydrurus	2	13.0%	0.1%	0.2%	2.2%	3.4%	1.1%	0.0%	0.0%	7.8%	4.3%	55.0%	30.0%	52.3%
	Eukaryota;__SAR;__Stramenopiles;__Chrysophyceae;__LG21-05;__uncultured_eukaryote	0	0.0%	0.0%	0.0%	0.0%	0.0%	0.0%	0.0%	0.0%	0.0%	0.0%	0.0%	0.0%	0.0%
	Eukaryota;__SAR;__Stramenopiles;__Chrysophyceae;__LG21-05;__uncultured_marine_eukaryote	0	0.0%	0.0%	0.0%	0.0%	0.0%	0.0%	0.0%	0.0%	0.0%	0.0%	0.0%	0.0%	0.0%
	Eukaryota;__SAR;__Stramenopiles;__Chrysophyceae;__Ochromonadales;__Ochromonas	0	0.0%	0.0%	0.0%	0.0%	0.0%	0.0%	0.0%	0.0%	0.0%	0.0%	0.0%	0.0%	0.0%
	Eukaryota;__SAR;__Stramenopiles;__Chrysophyceae;__Ochromonadales;__Paraphysomonas	0	0.0%	0.0%	0.0%	0.0%	0.0%	0.0%	0.0%	0.0%	0.0%	0.0%	0.0%	0.0%	0.0%
	Eukaryota;__SAR;__Stramenopiles;__Chrysophyceae;__uncultured_eukaryote;Other	0	0.0%	0.0%	0.0%	0.0%	0.0%	0.0%	0.0%	0.0%	0.0%	0.0%	0.0%	0.0%	0.0%
	Eukaryota;__SAR;__Stramenopiles;__Diatomea;__Bacillariophytina;__Bacillariophyceae	0	0.0%	0.0%	0.0%	0.0%	0.0%	0.1%	0.0%	0.0%	0.0%	0.0%	0.0%	0.0%	0.0%
	Eukaryota;__SAR;__Stramenopiles;__Eustigmatales;__Nannochloropsis;__uncultured_marine_eukaryote	0	0.0%	0.0%	0.0%	0.0%	0.0%	0.0%	0.0%	0.0%	0.0%	0.0%	0.0%	0.0%	0.0%
	Eukaryota;__SAR;__Stramenopiles;__Labyrinthulomycetes;__Thraustochytriaceae;__Schizochytrium	0	0.0%	0.0%	0.0%	0.0%	0.0%	0.0%	0.0%	0.0%	0.0%	0.0%	0.0%	0.0%	0.0%
	Eukaryota;__SAR;__Stramenopiles;__Peronosporomycetes;__Aphanomyces;__uncultured_Saprolegniales	0	0.0%	0.0%	0.0%	0.0%	0.0%	0.0%	0.0%	0.0%	0.0%	0.0%	0.0%	0.0%	0.0%
	Eukaryota;__SAR;__Stramenopiles;__Peronosporomycetes;__Lagenidium;__Lagenidium_caudatum	0	0.0%	0.0%	0.0%	0.0%	0.0%	0.0%	0.0%	0.0%	0.0%	0.0%	0.0%	0.0%	0.0%
	Eukaryota;__SAR;__Stramenopiles;__Peronosporomycetes;__Ochromonadaceae_environmental_sample;Other	0	0.0%	0.0%	0.0%	0.0%	0.0%	0.0%	0.0%	0.0%	0.0%	0.0%	0.0%	0.0%	0.0%
	Eukaryota;__SAR;__Stramenopiles;__Peronosporomycetes;__Phytophthora;__Halophytophthora	0	0.0%	0.0%	0.0%	0.0%	0.0%	0.0%	0.0%	0.0%	0.0%	0.0%	0.0%	0.0%	0.0%
	Eukaryota;__SAR;__Stramenopiles;__Peronosporomycetes;__Phytophthora;__uncultured_soil_fungus	0	0.0%	0.0%	0.0%	0.0%	0.0%	0.0%	0.0%	0.0%	0.0%	0.0%	0.0%	0.0%	0.0%
	Eukaryota;__SAR;__Stramenopiles;__Peronosporomycetes;__Saprolegnia;__Saprolegnia_sp._SAP1	0	0.0%	0.0%	0.0%	0.0%	0.0%	0.0%	0.0%	0.0%	0.0%	0.0%	0.0%	0.0%	0.0%
	Eukaryota;__SAR;__Stramenopiles;__Synurales;__Synura;__Synura_uvella	0	0.1%	0.0%	0.0%	0.0%	0.0%	0.0%	0.0%	0.4%	0.0%	0.0%	0.0%	0.4%	0.0%
	Eukaryota;__SAR;__Stramenopiles;__TKR07M.92;__uncultured_eukaryote;Other	0	0.0%	0.0%	0.0%	0.0%	0.0%	0.0%	0.0%	0.0%	0.0%	0.0%	0.0%	0.0%	0.0%
	Eukaryota;__SAR;__Stramenopiles;__Xanthophyceae;__Tribonematales;__Botrydiopsis	0	0.0%	0.0%	0.2%	0.0%	0.0%	0.0%	0.2%	0.0%	0.0%	0.0%	0.0%	0.0%	0.0%
	Eukaryota;__SAR;__Stramenopiles;__uncultured_eukaryote;Other;Other	0	0.8%	0.0%	0.0%	0.0%	0.0%	7.6%	0.3%	1.9%	0.0%	0.0%	0.0%	0.0%	0.0%
	Eukaryota;__Zeuk77;__uncultured_Oxytrichidae;Other;Other;Other	0	0.1%	0.1%	1.4%	0.2%	0.0%	0.0%	0.0%	0.0%	0.0%	0.0%	0.0%	0.0%	0.0%
	Eukaryota;__Zeuk77;__uncultured_eukaryote;Other;Other;Other	0	0.1%	0.0%	0.2%	0.0%	0.0%	0.1%	0.0%	0.0%	0.0%	0.0%	0.4%	0.2%	0.1%
	No blast hit;Other;Other;Other;Other;Other	0	0.2%	0.0%	0.0%	0.0%	0.0%	0.1%	0.0%	0.3%	0.0%	0.0%	0.2%	0.9%	1.0%
