# Supplementary material for: Microbial diversity on Icelandic glaciers and ice caps
Source: Front Microbiol. 2015 Apr 20;6:307. doi: 10.3389/fmicb.2015.00307 (PMC4403510; doi:10.3389/fmicb.2015.00307)
Supplement: Supplementary file 3 [file DataSheet3.PDF]

Taxonomy Summary. Current Level:

View Figure (.pdf) View Legend (.pdf)

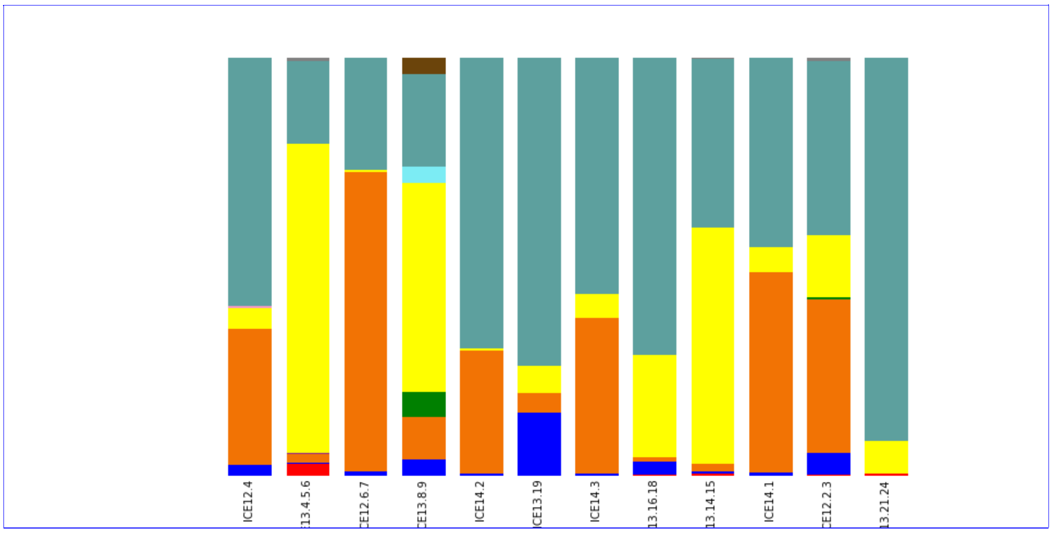

View Table (.txt)

Legend	Taxonomy	Total count	Total %	ICE12.4 %	ICE13.4.5.6 %	ICE12.6.7 %	ICE13.8.9 %	ICE14.2 %	ICE13.19 %	ICE14.3 %	ICE13.16.18 %	ICE13.14.15 %	ICE14.1 %	ICE12.2.3 %	ICE13.21.24 %
	k__Bacteria;p__Acidobacteria	0	0.4%	0.0%	2.9%	0.1%	0.0%	0.0%	0.0%	0.0%	0.2%	0.5%	0.0%	0.3%	0.5%
	k__Bacteria;p__Actinobacteria	0	2.8%	2.7%	0.3%	0.9%	4.0%	0.5%	15.0%	0.6%	3.1%	0.5%	0.8%	5.1%	0.0%
	k__Bacteria;p__Bacteroidetes	3	22.9%	32.4%	2.1%	71.6%	10.0%	29.3%	4.9%	37.2%	1.2%	1.9%	47.8%	36.8%	0.1%
	k__Bacteria;p__Chlorobi	0	0.5%	0.0%	0.0%	0.0%	6.0%	0.0%	0.0%	0.0%	0.0%	0.0%	0.0%	0.4%	0.0%
	k__Bacteria;p__Chloroflexi	0	0.0%	0.0%	0.2%	0.0%	0.0%	0.0%	0.0%	0.0%	0.0%	0.0%	0.0%	0.0%	0.0%
	k__Bacteria;p__Cyanobacteria	3	21.0%	5.0%	74.0%	0.6%	50.0%	0.8%	6.4%	5.7%	24.4%	56.4%	6.1%	14.9%	7.7%
	k__Bacteria;p__Firmicutes	0	0.3%	0.0%	0.0%	0.0%	4.0%	0.0%	0.1%	0.0%	0.0%	0.0%	0.0%	0.0%	0.0%
	k__Bacteria;p__Gemmatimonadetes	0	0.0%	0.5%	0.0%	0.0%	0.0%	0.0%	0.1%	0.0%	0.0%	0.0%	0.0%	0.1%	0.0%
	k__Bacteria;p__Proteobacteria	6	51.5%	59.5%	19.9%	26.8%	22.0%	69.4%	73.6%	56.5%	71.1%	40.4%	45.3%	41.6%	91.6%
	k__Bacteria;p__Spirochaetes	0	0.3%	0.0%	0.0%	0.0%	4.0%	0.0%	0.0%	0.0%	0.0%	0.0%	0.0%	0.0%	0.0%
	k__Bacteria;p__WPS-2	0	0.2%	0.0%	0.7%	0.0%	0.0%	0.0%	0.0%	0.0%	0.0%	0.3%	0.0%	0.8%	0.1%

Taxonomy Summary. Current Level:

View Figure (.pdf) View Legend (.pdf)

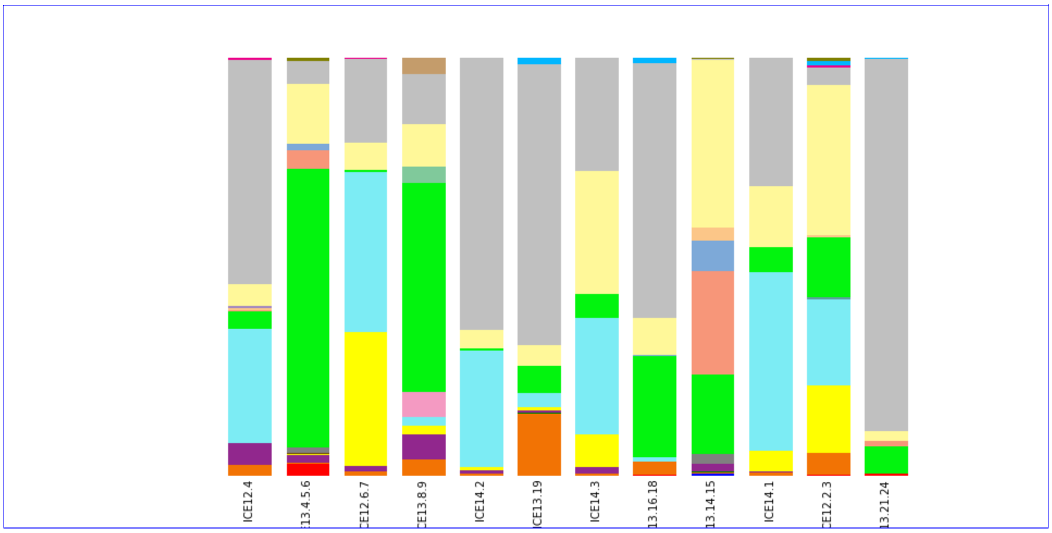

View Table (.txt)

Legend	Taxonomy	Total count	Total %	ICE12.4 %	ICE13.4.5.6 %	ICE12.6.7 %	ICE13.8.9 %	ICE14.2 %	ICE13.19 %	ICE14.3 %	ICE13.16.18 %	ICE13.14.15 %	ICE14.1 %	ICE12.2.3 %	ICE13.21.24 %
	k__Bacteria;p__Acidobacteria;c__Acidobacteriia	0	0.3%	0.0%	2.9%	0.1%	0.0%	0.0%	0.0%	0.0%	0.2%	0.0%	0.0%	0.3%	0.5%
	k__Bacteria;p__Acidobacteria;c__Solibacteres	0	0.0%	0.0%	0.0%	0.0%	0.0%	0.0%	0.0%	0.0%	0.0%	0.5%	0.0%	0.0%	0.0%
	k__Bacteria;p__Actinobacteria;c__Actinobacteria	0	2.8%	2.5%	0.3%	0.9%	4.0%	0.5%	14.9%	0.6%	3.1%	0.3%	0.8%	5.1%	0.0%
	k__Bacteria;p__Actinobacteria;c__Thermoleophilia	0	0.0%	0.2%	0.0%	0.0%	0.0%	0.0%	0.1%	0.0%	0.0%	0.3%	0.0%	0.0%	0.0%
	k__Bacteria;p__Bacteroidetes;c__Cytophagia	0	1.6%	5.2%	1.8%	1.4%	6.0%	0.7%	0.7%	1.6%	0.0%	1.9%	0.1%	0.1%	0.1%
	k__Bacteria;p__Bacteroidetes;c__Sphingobacteriia	1	5.4%	0.0%	0.2%	32.0%	2.0%	0.8%	0.6%	7.6%	0.2%	0.0%	5.0%	16.1%	0.0%
	k__Bacteria;p__Bacteroidetes;c__[Saprospirae]	2	15.9%	27.3%	0.1%	38.2%	2.0%	27.8%	3.6%	28.0%	1.0%	0.0%	42.7%	20.6%	0.0%
	k__Bacteria;p__Chlorobi;c__Ignavibacteria	0	0.5%	0.0%	0.0%	0.0%	6.0%	0.0%	0.0%	0.0%	0.0%	0.0%	0.0%	0.0%	0.0%
	k__Bacteria;p__Chlorobi;c__SJA-28	0	0.0%	0.0%	0.0%	0.0%	0.0%	0.0%	0.0%	0.0%	0.0%	0.0%	0.0%	0.4%	0.0%
	k__Bacteria;p__Chloroflexi;c__Ktedonobacteria	0	0.0%	0.0%	0.2%	0.0%	0.0%	0.0%	0.0%	0.0%	0.0%	0.0%	0.0%	0.0%	0.0%
	k__Bacteria;p__Cyanobacteria;c__	0	0.3%	0.0%	1.4%	0.0%	0.0%	0.0%	0.0%	0.0%	0.0%	2.2%	0.0%	0.0%	0.0%
	k__Bacteria;p__Cyanobacteria;c__Chloroplast	2	17.0%	4.3%	66.5%	0.6%	50.0%	0.8%	6.4%	5.7%	24.1%	19.0%	6.1%	14.5%	6.4%
	k__Bacteria;p__Cyanobacteria;c__Nostocophycideae	0	2.6%	0.2%	4.6%	0.0%	0.0%	0.0%	0.0%	0.0%	0.0%	24.9%	0.0%	0.0%	1.4%
	k__Bacteria;p__Cyanobacteria;c__Oscillatoriophycideae	0	0.8%	0.0%	1.4%	0.0%	0.0%	0.0%	0.0%	0.0%	0.3%	7.3%	0.0%	0.0%	0.0%
	k__Bacteria;p__Cyanobacteria;c__Synechococcophycideae	0	0.3%	0.5%	0.1%	0.0%	0.0%	0.0%	0.0%	0.0%	0.0%	3.0%	0.0%	0.4%	0.0%
	k__Bacteria;p__Firmicutes;c__Bacilli	0	0.3%	0.0%	0.0%	0.0%	4.0%	0.0%	0.1%	0.0%	0.0%	0.0%	0.0%	0.0%	0.0%
	k__Bacteria;p__Gemmatimonadetes;c__Gemmatimonadetes	0	0.0%	0.5%	0.0%	0.0%	0.0%	0.0%	0.1%	0.0%	0.0%	0.0%	0.0%	0.1%	0.0%
	k__Bacteria;p__Proteobacteria;c__Alphaproteobacteria	2	14.7%	5.2%	14.3%	6.6%	10.0%	4.4%	4.8%	29.4%	8.8%	40.1%	14.7%	36.0%	2.5%
	k__Bacteria;p__Proteobacteria;c__Betaproteobacteria	4	36.3%	53.8%	5.4%	20.0%	12.0%	64.9%	67.3%	27.1%	60.9%	0.3%	30.6%	4.2%	89.1%
	k__Bacteria;p__Proteobacteria;c__Deltaproteobacteria	0	0.1%	0.5%	0.1%	0.1%	0.0%	0.0%	0.0%	0.0%	0.0%	0.0%	0.0%	0.3%	0.0%
	k__Bacteria;p__Proteobacteria;c__Gammaproteobacteria	0	0.4%	0.0%	0.1%	0.1%	0.0%	0.1%	1.5%	0.1%	1.4%	0.0%	0.0%	1.1%	0.1%
	k__Bacteria;p__Spirochaetes;c__Spirochaetes	0	0.3%	0.0%	0.0%	0.0%	4.0%	0.0%	0.0%	0.0%	0.0%	0.0%	0.0%	0.0%	0.0%
	k__Bacteria;p__WPS-2;c__	0	0.2%	0.0%	0.7%	0.0%	0.0%	0.0%	0.0%	0.0%	0.0%	0.3%	0.0%	0.8%	0.1%

Taxonomy Summary. Current Level:

View Figure (.pdf) View Legend (.pdf)

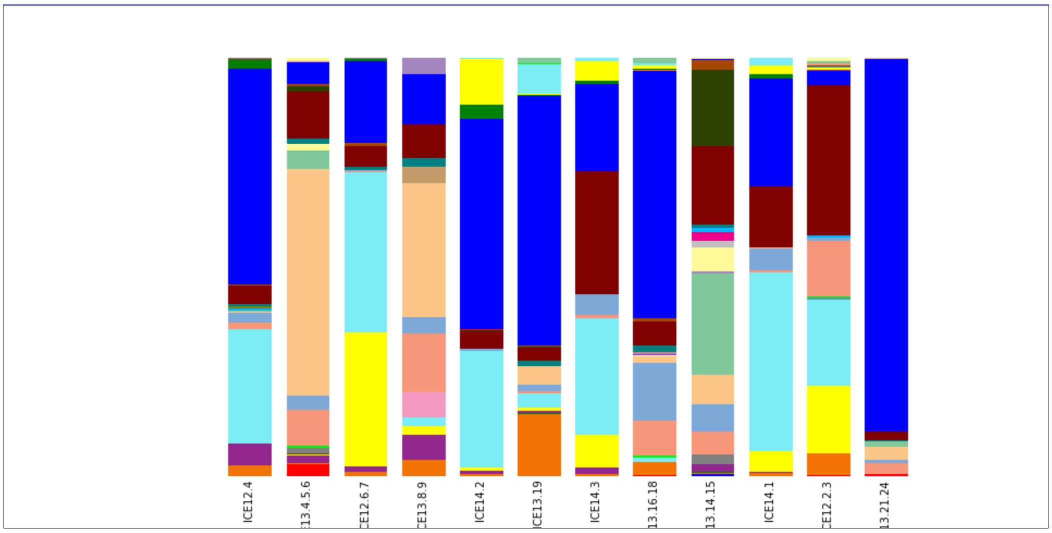

View Table (.txt)

		Total		ICE12.4	ICE13.4.5.6	ICE12.6.7	ICE13.8.9	ICE14.2	ICE13.19	ICE14.3	ICE13.16.18	ICE13.14.15	ICE14.1	ICE12.2.3	ICE13.21.24
Legend	Taxonomy	count	%	%	%	%	%	%	%	%	%	%	%	%	%
	k__Bacteria;p__Acidobacteria;c__Acidobacteriia;o__Acidobacteriales	0	0.3%	0.0%	2.9%	0.1%	0.0%	0.0%	0.0%	0.0%	0.2%	0.0%	0.0%	0.3%	0.5%
	k__Bacteria;p__Acidobacteria;c__Solibacteres;o__Solibacterales	0	0.0%	0.0%	0.0%	0.0%	0.0%	0.0%	0.0%	0.0%	0.0%	0.5%	0.0%	0.0%	0.0%
	k__Bacteria;p__Actinobacteria;c__Actinobacteria;o__Actinomycetales	0	2.8%	2.5%	0.3%	0.9%	4.0%	0.5%	14.9%	0.6%	3.1%	0.3%	0.8%	5.1%	0.0%
	k__Bacteria;p__Actinobacteria;c__Thermoleophilia;o__Gaiellales	0	0.0%	0.2%	0.0%	0.0%	0.0%	0.0%	0.1%	0.0%	0.0%	0.3%	0.0%	0.0%	0.0%
	k__Bacteria;p__Bacteroidetes;c__Cytophagia;o__Cytophagales	0	1.6%	5.2%	1.8%	1.4%	6.0%	0.7%	0.7%	1.6%	0.0%	1.9%	0.1%	0.1%	0.1%
	k__Bacteria;p__Bacteroidetes;c__Sphingobacteriia;o__Sphingobacteriales	1	5.4%	0.0%	0.2%	32.0%	2.0%	0.8%	0.6%	7.6%	0.2%	0.0%	5.0%	16.1%	0.0%
	k__Bacteria;p__Bacteroidetes;c__[Saprospirae];o__[Saprospirales]	2	15.9%	27.3%	0.1%	38.2%	2.0%	27.8%	3.6%	28.0%	1.0%	0.0%	42.7%	20.6%	0.0%
	k__Bacteria;p__Chlorobi;c__Ignavibacteria;o__Ignavibacteriales	0	0.5%	0.0%	0.0%	0.0%	6.0%	0.0%	0.0%	0.0%	0.0%	0.0%	0.0%	0.0%	0.0%
	k__Bacteria;p__Chlorobi;c__SJA-28;o__	0	0.0%	0.0%	0.0%	0.0%	0.0%	0.0%	0.0%	0.0%	0.0%	0.0%	0.0%	0.4%	0.0%
	k__Bacteria;p__Chloroflexi;c__Ktedonobacteria;o__Thermogemmatisporales	0	0.0%	0.0%	0.2%	0.0%	0.0%	0.0%	0.0%	0.0%	0.0%	0.0%	0.0%	0.0%	0.0%
	k__Bacteria;p__Cyanobacteria;c__;o__	0	0.3%	0.0%	1.4%	0.0%	0.0%	0.0%	0.0%	0.0%	0.0%	2.2%	0.0%	0.0%	0.0%
	k__Bacteria;p__Cyanobacteria;c__Chloroplast;o__	0	0.1%	0.0%	0.4%	0.0%	0.0%	0.1%	0.0%	0.0%	0.5%	0.0%	0.1%	0.4%	0.1%
	k__Bacteria;p__Cyanobacteria;c__Chloroplast;o__Chlorophyta	1	4.6%	1.6%	8.6%	0.4%	14.0%	0.1%	0.4%	0.7%	8.3%	5.4%	0.5%	13.2%	2.4%
	k__Bacteria;p__Cyanobacteria;c__Chloroplast;o__Stramenopiles	0	3.7%	2.5%	3.4%	0.2%	4.0%	0.5%	1.7%	4.9%	13.9%	6.5%	5.2%	0.7%	0.9%
	k__Bacteria;p__Cyanobacteria;c__Chloroplast;o__Streptophyta	1	8.5%	0.2%	54.2%	0.0%	32.0%	0.1%	4.3%	0.0%	1.4%	7.0%	0.2%	0.1%	2.9%
	k__Bacteria;p__Cyanobacteria;c__Nostocophycideae;o__Nostocales	0	2.5%	0.2%	4.6%	0.0%	0.0%	0.0%	0.0%	0.0%	0.0%	24.4%	0.0%	0.0%	1.4%
	k__Bacteria;p__Cyanobacteria;c__Nostocophycideae;o__Stigonematales	0	0.0%	0.0%	0.0%	0.0%	0.0%	0.0%	0.0%	0.0%	0.0%	0.5%	0.0%	0.0%	0.0%
	k__Bacteria;p__Cyanobacteria;c__Oscillatoriophycideae;o__Chroococcales	0	0.6%	0.0%	1.4%	0.0%	0.0%	0.0%	0.0%	0.0%	0.3%	5.7%	0.0%	0.0%	0.0%
	k__Bacteria;p__Cyanobacteria;c__Oscillatoriophycideae;o__Oscillatoriales	0	0.1%	0.0%	0.0%	0.0%	0.0%	0.0%	0.0%	0.0%	0.0%	1.6%	0.0%	0.0%	0.0%
	k__Bacteria;p__Cyanobacteria;c__Synechococcophycideae;o__Pseudanabaenales	0	0.2%	0.0%	0.0%	0.0%	0.0%	0.0%	0.0%	0.0%	0.0%	1.9%	0.0%	0.0%	0.0%
	k__Bacteria;p__Cyanobacteria;c__Synechococcophycideae;o__Synechococcales	0	0.2%	0.5%	0.1%	0.0%	0.0%	0.0%	0.0%	0.0%	0.0%	1.1%	0.0%	0.4%	0.0%
	k__Bacteria;p__Firmicutes;c__Bacilli;o__Bacillales	0	0.3%	0.0%	0.0%	0.0%	4.0%	0.0%	0.1%	0.0%	0.0%	0.0%	0.0%	0.0%	0.0%
	k__Bacteria;p__Gemmatimonadetes;c__Gemmatimonadetes;o__Gemmatimonadales	0	0.0%	0.5%	0.0%	0.0%	0.0%	0.0%	0.1%	0.0%	0.0%	0.0%	0.0%	0.1%	0.0%
	k__Bacteria;p__Proteobacteria;c__Alphaproteobacteria;o__	0	0.0%	0.0%	0.0%	0.0%	0.0%	0.0%	0.0%	0.0%	0.3%	0.0%	0.0%	0.0%	0.0%
	k__Bacteria;p__Proteobacteria;c__Alphaproteobacteria;o__Caulobacterales	0	0.0%	0.0%	0.0%	0.0%	0.0%	0.0%	0.0%	0.0%	0.3%	0.0%	0.0%	0.0%	0.0%
	k__Bacteria;p__Proteobacteria;c__Alphaproteobacteria;o__Rhizobiales	0	0.7%	0.7%	1.3%	0.8%	2.0%	0.0%	1.1%	0.0%	1.5%	0.8%	0.1%	0.1%	0.4%
	k__Bacteria;p__Proteobacteria;c__Alphaproteobacteria;o__Rhodospirillales	1	11.8%	4.3%	11.2%	4.8%	8.0%	4.2%	3.3%	29.3%	5.9%	18.7%	14.6%	35.7%	1.9%
	k__Bacteria;p__Proteobacteria;c__Alphaproteobacteria;o__Rickettsiales	0	1.6%	0.0%	1.3%	0.0%	0.0%	0.1%	0.1%	0.0%	0.0%	18.2%	0.0%	0.0%	0.0%
	k__Bacteria;p__Proteobacteria;c__Alphaproteobacteria;o__Sphingomonadales	0	0.5%	0.2%	0.4%	0.9%	0.0%	0.2%	0.3%	0.0%	0.7%	2.4%	0.0%	0.2%	0.1%
	k__Bacteria;p__Proteobacteria;c__Betaproteobacteria;o__	0	0.0%	0.0%	0.0%	0.0%	0.0%	0.1%	0.0%	0.0%	0.0%	0.0%	0.0%	0.0%	0.0%
	k__Bacteria;p__Proteobacteria;c__Betaproteobacteria;o__Burkholderiales	4	33.1%	51.8%	5.3%	19.5%	12.0%	50.4%	59.8%	20.8%	59.2%	0.3%	25.6%	3.4%	89.1%
	k__Bacteria;p__Proteobacteria;c__Betaproteobacteria;o__Ellin6067	0	0.0%	0.0%	0.0%	0.0%	0.0%	0.0%	0.0%	0.0%	0.3%	0.0%	0.0%	0.0%	0.0%
	k__Bacteria;p__Proteobacteria;c__Betaproteobacteria;o__Gallionellales	0	0.7%	2.0%	0.0%	0.4%	0.0%	3.4%	0.2%	0.9%	0.2%	0.0%	1.0%	0.0%	0.0%
	k__Bacteria;p__Proteobacteria;c__Betaproteobacteria;o__IS-44	0	0.0%	0.0%	0.0%	0.0%	0.0%	0.0%	0.0%	0.0%	0.0%	0.0%	0.0%	0.2%	0.0%
	k__Bacteria;p__Proteobacteria;c__Betaproteobacteria;o__Methylophilales	0	1.6%	0.0%	0.0%	0.0%	0.0%	10.8%	0.1%	4.7%	0.7%	0.0%	2.2%	0.4%	0.0%
	k__Bacteria;p__Proteobacteria;c__Betaproteobacteria;o__Rhodocyclales	0	0.9%	0.0%	0.1%	0.1%	0.0%	0.3%	7.2%	0.6%	0.5%	0.0%	1.8%	0.1%	0.0%
	k__Bacteria;p__Proteobacteria;c__Betaproteobacteria;o__SC-I-84	0	0.0%	0.0%	0.1%	0.0%	0.0%	0.0%	0.0%	0.0%	0.0%	0.0%	0.0%	0.0%	0.0%
	k__Bacteria;p__Proteobacteria;c__Deltaproteobacteria;o__MBNT15	0	0.0%	0.0%	0.0%	0.0%	0.0%	0.0%	0.0%	0.0%	0.0%	0.0%	0.0%	0.1%	0.0%
	k__Bacteria;p__Proteobacteria;c__Deltaproteobacteria;o__Myxococcales	0	0.0%	0.2%	0.1%	0.0%	0.0%	0.0%	0.0%	0.0%	0.0%	0.0%	0.0%	0.2%	0.0%
	k__Bacteria;p__Proteobacteria;c__Deltaproteobacteria;o__Spirobacillales	0	0.0%	0.2%	0.0%	0.1%	0.0%	0.0%	0.0%	0.0%	0.0%	0.0%	0.0%	0.0%	0.0%
	k__Bacteria;p__Proteobacteria;c__Gammaproteobacteria;o__	0	0.0%	0.0%	0.0%	0.0%	0.0%	0.0%	0.2%	0.0%	0.0%	0.0%	0.0%	0.0%	0.0%
	k__Bacteria;p__Proteobacteria;c__Gammaproteobacteria;o__Legionellales	0	0.1%	0.0%	0.0%	0.0%	0.0%	0.0%	0.0%	0.0%	0.0%	0.0%	0.0%	0.7%	0.0%
	k__Bacteria;p__Proteobacteria;c__Gammaproteobacteria;o__Pseudomonadales	0	0.0%	0.0%	0.1%	0.0%	0.0%	0.0%	0.0%	0.0%	0.0%	0.0%	0.0%	0.0%	0.0%
	k__Bacteria;p__Proteobacteria;c__Gammaproteobacteria;o__Thiotrichales	0	0.0%	0.0%	0.0%	0.0%	0.0%	0.0%	0.0%	0.0%	0.2%	0.0%	0.0%	0.0%	0.1%
	k__Bacteria;p__Proteobacteria;c__Gammaproteobacteria;o__Xanthomonadales	0	0.3%	0.0%	0.0%	0.1%	0.0%	0.1%	1.3%	0.0%	1.2%	0.0%	0.0%	0.4%	0.0%
	k__Bacteria;p__Spirochaetes;c__Spirochaetes;o__Spirochaetales	0	0.3%	0.0%	0.0%	0.0%	4.0%	0.0%	0.0%	0.0%	0.0%	0.0%	0.0%	0.0%	0.0%
	k__Bacteria;p__WPS-2;c__;o__	0	0.2%	0.0%	0.7%	0.0%	0.0%	0.0%	0.0%	0.0%	0.0%	0.3%	0.0%	0.8%	0.1%

Taxonomy Summary. Current Level:

View Figure (.pdf) View Legend (.pdf)

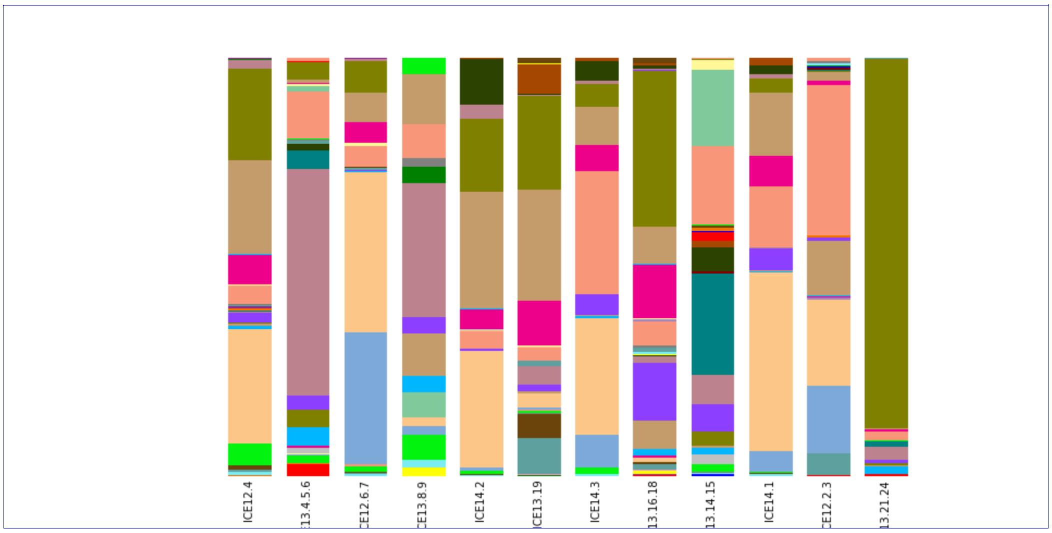

View Table (.txt)

		Total		ICE12.4	ICE13.4.5.6	ICE12.6.7	ICE13.8.9	ICE14.2	ICE13.19	ICE14.3	ICE13.16.18	ICE13.14.15	ICE14.1	ICE12.2.3	ICE13.21.24
Legend	Taxonomy	count	%	%	%	%	%	%	%	%	%	%	%	%	%

	k__Bacteria;p__Acidobacteria;c__Acidobacteriia;o__Acidobacteriales;f__Acidobacteriaceae	0	0.3%	0.0%	2.9%	0.1%	0.0%	0.0%	0.0%	0.0%	0.2%	0.0%	0.0%	0.3%	0.5%
	k__Bacteria;p__Acidobacteria;c__Solibacteres;o__Solibacterales;f__	0	0.0%	0.0%	0.0%	0.0%	0.0%	0.0%	0.0%	0.0%	0.0%	0.5%	0.0%	0.0%	0.0%
	k__Bacteria;p__Actinobacteria;c__Actinobacteria;o__Actinomycetales;f__	0	0.0%	0.2%	0.2%	0.0%	0.0%	0.0%	0.1%	0.0%	0.0%	0.0%	0.0%	0.0%	0.0%
	k__Bacteria;p__Actinobacteria;c__Actinobacteria;o__Actinomycetales;f__Frankiaceae	0	0.0%	0.0%	0.0%	0.0%	0.0%	0.0%	0.1%	0.0%	0.0%	0.0%	0.0%	0.0%	0.0%
	k__Bacteria;p__Actinobacteria;c__Actinobacteria;o__Actinomycetales;f__Geodermatophilaceae	0	0.0%	0.0%	0.0%	0.0%	0.0%	0.0%	0.0%	0.0%	0.3%	0.0%	0.0%	0.0%	0.0%
	k__Bacteria;p__Actinobacteria;c__Actinobacteria;o__Actinomycetales;f__Kineosporiaceae	0	0.2%	0.0%	0.0%	0.0%	2.0%	0.0%	0.0%	0.0%	0.9%	0.0%	0.0%	0.0%	0.0%
	k__Bacteria;p__Actinobacteria;c__Actinobacteria;o__Actinomycetales;f__Microbacteriaceae	0	0.4%	0.9%	0.0%	0.4%	2.0%	0.2%	0.2%	0.4%	0.0%	0.3%	0.5%	0.1%	0.0%
	k__Bacteria;p__Actinobacteria;c__Actinobacteria;o__Actinomycetales;f__Nocardiaceae	0	0.0%	0.0%	0.0%	0.0%	0.0%	0.0%	0.1%	0.0%	0.2%	0.0%	0.0%	0.0%	0.0%
	k__Bacteria;p__Actinobacteria;c__Actinobacteria;o__Actinomycetales;f__Pseudonocardiaceae	0	1.3%	0.5%	0.1%	0.2%	0.0%	0.0%	8.7%	0.0%	1.2%	0.0%	0.1%	5.1%	0.0%
	k__Bacteria;p__Actinobacteria;c__Actinobacteria;o__Actinomycetales;f__Sporichthyaceae	0	0.7%	0.9%	0.0%	0.4%	0.0%	0.4%	5.7%	0.2%	0.5%	0.0%	0.3%	0.0%	0.0%
	k__Bacteria;p__Actinobacteria;c__Thermoleophilia;o__Gaiellales;f__	0	0.0%	0.2%	0.0%	0.0%	0.0%	0.0%	0.1%	0.0%	0.0%	0.3%	0.0%	0.0%	0.0%
	k__Bacteria;p__Bacteroidetes;c__Cytophagia;o__Cytophagales;f__Cytophagaceae	0	1.6%	5.2%	1.8%	1.4%	6.0%	0.7%	0.7%	1.6%	0.0%	1.9%	0.1%	0.1%	0.1%
	k__Bacteria;p__Bacteroidetes;c__Sphingobacteriia;o__Sphingobacteriales;f__	0	0.0%	0.0%	0.0%	0.4%	0.0%	0.0%	0.2%	0.0%	0.0%	0.0%	0.0%	0.0%	0.0%
	k__Bacteria;p__Bacteroidetes;c__Sphingobacteriia;o__Sphingobacteriales;f__Sphingobacteriaceae	1	5.3%	0.0%	0.2%	31.6%	2.0%	0.8%	0.4%	7.6%	0.2%	0.0%	5.0%	16.1%	0.0%
	k__Bacteria;p__Bacteroidetes;c__[Saprospirae];o__[Saprospirales];f__Chitinophagaceae	2	15.9%	27.3%	0.1%	38.2%	2.0%	27.8%	3.6%	28.0%	1.0%	0.0%	42.7%	20.6%	0.0%
	k__Bacteria;p__Chlorobi;c__Ignavibacteria;o__Ignavibacteriales;f__Ignavibacteriaceae	0	0.5%	0.0%	0.0%	0.0%	6.0%	0.0%	0.0%	0.0%	0.0%	0.0%	0.0%	0.0%	0.0%
	k__Bacteria;p__Chlorobi;c__SJA-28;o__;f__	0	0.0%	0.0%	0.0%	0.0%	0.0%	0.0%	0.0%	0.0%	0.0%	0.0%	0.0%	0.4%	0.0%
	k__Bacteria;p__Chloroflexi;c__Ktedonobacteria;o__Thermogemmatisporales;f__Thermogemmatisporaceae	0	0.0%	0.0%	0.2%	0.0%	0.0%	0.0%	0.0%	0.0%	0.0%	0.0%	0.0%	0.0%	0.0%
	k__Bacteria;p__Cyanobacteria;c__;o__;f__	0	0.3%	0.0%	1.4%	0.0%	0.0%	0.0%	0.0%	0.0%	0.0%	2.2%	0.0%	0.0%	0.0%
	k__Bacteria;p__Cyanobacteria;c__Chloroplast;o__;f__	0	0.1%	0.0%	0.4%	0.0%	0.0%	0.1%	0.0%	0.0%	0.5%	0.0%	0.1%	0.4%	0.1%
	k__Bacteria;p__Cyanobacteria;c__Chloroplast;o__Chlorophyta;f__	0	1.2%	0.7%	4.4%	0.2%	4.0%	0.1%	0.0%	0.5%	1.5%	1.6%	0.1%	0.1%	1.6%
	k__Bacteria;p__Cyanobacteria;c__Chloroplast;o__Chlorophyta;f__Chlamydomonadaceae	0	2.7%	0.7%	0.1%	0.1%	10.0%	0.0%	0.4%	0.3%	6.7%	0.5%	0.4%	13.0%	0.3%
	k__Bacteria;p__Cyanobacteria;c__Chloroplast;o__Chlorophyta;f__Trebouxiophyceae	0	0.7%	0.2%	4.0%	0.0%	0.0%	0.0%	0.0%	0.0%	0.0%	3.3%	0.0%	0.0%	0.6%
	k__Bacteria;p__Cyanobacteria;c__Chloroplast;o__Stramenopiles;f__	0	3.7%	2.5%	3.4%	0.2%	4.0%	0.5%	1.7%	4.9%	13.9%	6.5%	5.2%	0.7%	0.9%
	k__Bacteria;p__Cyanobacteria;c__Chloroplast;o__Streptophyta;f__	1	8.5%	0.2%	54.2%	0.0%	32.0%	0.1%	4.3%	0.0%	1.4%	7.0%	0.2%	0.1%	2.9%
	k__Bacteria;p__Cyanobacteria;c__Nostocophycideae;o__Nostocales;f__Nostocaceae	0	2.5%	0.2%	4.6%	0.0%	0.0%	0.0%	0.0%	0.0%	0.0%	24.4%	0.0%	0.0%	1.4%
	k__Bacteria;p__Cyanobacteria;c__Nostocophycideae;o__Stigonematales;f__Rivulariaceae	0	0.0%	0.0%	0.0%	0.0%	0.0%	0.0%	0.0%	0.0%	0.0%	0.5%	0.0%	0.0%	0.0%
	k__Bacteria;p__Cyanobacteria;c__Oscillatoriophycideae;o__Chroococcales;f__Xenococcaceae	0	0.6%	0.0%	1.4%	0.0%	0.0%	0.0%	0.0%	0.0%	0.3%	5.7%	0.0%	0.0%	0.0%
	k__Bacteria;p__Cyanobacteria;c__Oscillatoriophycideae;o__Oscillatoriales;f__Phormidiaceae	0	0.1%	0.0%	0.0%	0.0%	0.0%	0.0%	0.0%	0.0%	0.0%	1.6%	0.0%	0.0%	0.0%
	k__Bacteria;p__Cyanobacteria;c__Synechococcophycideae;o__Pseudanabaenales;f__Pseudanabaenaceae	0	0.2%	0.0%	0.0%	0.0%	0.0%	0.0%	0.0%	0.0%	0.0%	1.9%	0.0%	0.0%	0.0%
	k__Bacteria;p__Cyanobacteria;c__Synechococcophycideae;o__Synechococcales;f__Acaryochloridaceae	0	0.0%	0.0%	0.1%	0.0%	0.0%	0.0%	0.0%	0.0%	0.0%	0.3%	0.0%	0.0%	0.0%
	k__Bacteria;p__Cyanobacteria;c__Synechococcophycideae;o__Synechococcales;f__Chamaesiphonaceae	0	0.1%	0.5%	0.1%	0.0%	0.0%	0.0%	0.0%	0.0%	0.0%	0.8%	0.0%	0.4%	0.0%
	k__Bacteria;p__Firmicutes;c__Bacilli;o__Bacillales;f__Alicyclobacillaceae	0	0.3%	0.0%	0.0%	0.0%	4.0%	0.0%	0.1%	0.0%	0.0%	0.0%	0.0%	0.0%	0.0%
	k__Bacteria;p__Gemmatimonadetes;c__Gemmatimonadetes;o__Gemmatimonadales;f__Ellin5301	0	0.0%	0.5%	0.0%	0.0%	0.0%	0.0%	0.1%	0.0%	0.0%	0.0%	0.0%	0.1%	0.0%
	k__Bacteria;p__Proteobacteria;c__Alphaproteobacteria;o__;f__	0	0.0%	0.0%	0.0%	0.0%	0.0%	0.0%	0.0%	0.0%	0.3%	0.0%	0.0%	0.0%	0.0%
	k__Bacteria;p__Proteobacteria;c__Alphaproteobacteria;o__Caulobacterales;f__Caulobacteraceae	0	0.0%	0.0%	0.0%	0.0%	0.0%	0.0%	0.0%	0.0%	0.3%	0.0%	0.0%	0.0%	0.0%
	k__Bacteria;p__Proteobacteria;c__Alphaproteobacteria;o__Rhizobiales;f__Beijerinckiaceae	0	0.0%	0.0%	0.1%	0.0%	0.0%	0.0%	0.0%	0.0%	0.0%	0.0%	0.0%	0.0%	0.0%
	k__Bacteria;p__Proteobacteria;c__Alphaproteobacteria;o__Rhizobiales;f__Bradyrhizobiaceae	0	0.3%	0.5%	0.7%	0.6%	0.0%	0.0%	1.1%	0.0%	1.2%	0.0%	0.1%	0.0%	0.0%
	k__Bacteria;p__Proteobacteria;c__Alphaproteobacteria;o__Rhizobiales;f__Hyphomicrobiaceae	0	0.1%	0.0%	0.0%	0.2%	0.0%	0.0%	0.1%	0.0%	0.0%	0.5%	0.0%	0.1%	0.0%
	k__Bacteria;p__Proteobacteria;c__Alphaproteobacteria;o__Rhizobiales;f__Methylobacteriaceae	0	0.2%	0.2%	0.2%	0.0%	2.0%	0.0%	0.0%	0.0%	0.3%	0.0%	0.0%	0.0%	0.0%
	k__Bacteria;p__Proteobacteria;c__Alphaproteobacteria;o__Rhizobiales;f__Methylocystaceae	0	0.1%	0.0%	0.3%	0.0%	0.0%	0.0%	0.0%	0.0%	0.0%	0.3%	0.0%	0.0%	0.4%
	k__Bacteria;p__Proteobacteria;c__Alphaproteobacteria;o__Rhodospirillales;f__Acetobacteraceae	1	11.8%	4.3%	11.2%	4.8%	8.0%	4.2%	3.3%	29.3%	5.9%	18.7%	14.5%	35.7%	1.9%
	k__Bacteria;p__Proteobacteria;c__Alphaproteobacteria;o__Rhodospirillales;f__Rhodospirillaceae	0	0.0%	0.0%	0.0%	0.0%	0.0%	0.0%	0.0%	0.0%	0.0%	0.0%	0.1%	0.0%	0.0%
	k__Bacteria;p__Proteobacteria;c__Alphaproteobacteria;o__Rickettsiales;f__	0	0.0%	0.0%	0.0%	0.0%	0.0%	0.1%	0.1%	0.0%	0.0%	0.0%	0.0%	0.0%	0.0%
	k__Bacteria;p__Proteobacteria;c__Alphaproteobacteria;o__Rickettsiales;f__mitochondria	0	1.6%	0.0%	1.3%	0.0%	0.0%	0.0%	0.0%	0.0%	0.0%	18.2%	0.0%	0.0%	0.0%
	k__Bacteria;p__Proteobacteria;c__Alphaproteobacteria;o__Sphingomonadales;f__	0	0.0%	0.0%	0.0%	0.0%	0.0%	0.0%	0.0%	0.0%	0.5%	0.0%	0.0%	0.0%	0.0%
	k__Bacteria;p__Proteobacteria;c__Alphaproteobacteria;o__Sphingomonadales;f__Sphingomonadaceae	0	0.4%	0.2%	0.4%	0.9%	0.0%	0.2%	0.3%	0.0%	0.2%	2.4%	0.0%	0.2%	0.1%
	k__Bacteria;p__Proteobacteria;c__Betaproteobacteria;o__;f__	0	0.0%	0.0%	0.0%	0.0%	0.0%	0.1%	0.0%	0.0%	0.0%	0.0%	0.0%	0.0%	0.0%
	k__Bacteria;p__Proteobacteria;c__Betaproteobacteria;o__Burkholderiales;f__	1	4.6%	7.2%	0.4%	4.8%	0.0%	4.7%	10.7%	6.3%	12.9%	0.0%	7.1%	1.0%	0.4%
	k__Bacteria;p__Proteobacteria;c__Betaproteobacteria;o__Burkholderiales;f__Burkholderiaceae	0	0.1%	0.2%	0.0%	0.1%	0.0%	0.2%	0.0%	0.0%	0.2%	0.0%	0.0%	0.0%	0.0%
	k__Bacteria;p__Proteobacteria;c__Betaproteobacteria;o__Burkholderiales;f__Comamonadaceae	1	11.0%	22.3%	0.7%	7.1%	12.0%	28.0%	26.5%	9.2%	8.8%	0.0%	15.3%	2.1%	0.1%
	k__Bacteria;p__Proteobacteria;c__Betaproteobacteria;o__Burkholderiales;f__Oxalobacteraceae	2	17.4%	22.1%	4.2%	7.5%	0.0%	17.5%	22.6%	5.3%	37.3%	0.3%	3.2%	0.3%	88.5%
	k__Bacteria;p__Proteobacteria;c__Betaproteobacteria;o__Ellin6067;f__	0	0.0%	0.0%	0.0%	0.0%	0.0%	0.0%	0.0%	0.0%	0.3%	0.0%	0.0%	0.0%	0.0%
	k__Bacteria;p__Proteobacteria;c__Betaproteobacteria;o__Gallionellales;f__Gallionellaceae	0	0.7%	2.0%	0.0%	0.4%	0.0%	3.4%	0.2%	0.9%	0.2%	0.0%	1.0%	0.0%	0.0%
	k__Bacteria;p__Proteobacteria;c__Betaproteobacteria;o__IS-44;f__	0	0.0%	0.0%	0.0%	0.0%	0.0%	0.0%	0.0%	0.0%	0.0%	0.0%	0.0%	0.2%	0.0%
	k__Bacteria;p__Proteobacteria;c__Betaproteobacteria;o__Methylophilales;f__	0	0.0%	0.0%	0.0%	0.0%	0.0%	0.0%	0.0%	0.0%	0.0%	0.0%	0.0%	0.4%	0.0%
	k__Bacteria;p__Proteobacteria;c__Betaproteobacteria;o__Methylophilales;f__Methylophilaceae	0	1.5%	0.0%	0.0%	0.0%	0.0%	10.8%	0.1%	4.7%	0.7%	0.0%	2.2%	0.0%	0.0%
	k__Bacteria;p__Proteobacteria;c__Betaproteobacteria;o__Rhodocyclales;f__Rhodocyclaceae	0	0.9%	0.0%	0.1%	0.1%	0.0%	0.3%	7.2%	0.6%	0.5%	0.0%	1.8%	0.1%	0.0%
	k__Bacteria;p__Proteobacteria;c__Betaproteobacteria;o__SC-I-84;f__	0	0.0%	0.0%	0.1%	0.0%	0.0%	0.0%	0.0%	0.0%	0.0%	0.0%	0.0%	0.0%	0.0%
	k__Bacteria;p__Proteobacteria;c__Deltaproteobacteria;o__MBNT15;f__	0	0.0%	0.0%	0.0%	0.0%	0.0%	0.0%	0.0%	0.0%	0.0%	0.0%	0.0%	0.1%	0.0%
	k__Bacteria;p__Proteobacteria;c__Deltaproteobacteria;o__Myxococcales;f__Haliangiaceae	0	0.0%	0.0%	0.0%	0.0%	0.0%	0.0%	0.0%	0.0%	0.0%	0.0%	0.0%	0.1%	0.0%
	k__Bacteria;p__Proteobacteria;c__Deltaproteobacteria;o__Myxococcales;f__Polyangiaceae	0	0.0%	0.2%	0.1%	0.0%	0.0%	0.0%	0.0%	0.0%	0.0%	0.0%	0.0%	0.1%	0.0%
	k__Bacteria;p__Proteobacteria;c__Deltaproteobacteria;o__Spirobacillales;f__	0	0.0%	0.2%	0.0%	0.1%	0.0%	0.0%	0.0%	0.0%	0.0%	0.0%	0.0%	0.0%	0.0%
	k__Bacteria;p__Proteobacteria;c__Gammaproteobacteria;o__;f__	0	0.0%	0.0%	0.0%	0.0%	0.0%	0.0%	0.2%	0.0%	0.0%	0.0%	0.0%	0.0%	0.0%
	k__Bacteria;p__Proteobacteria;c__Gammaproteobacteria;o__Legionellales;f__Coxiellaceae	0	0.1%	0.0%	0.0%	0.0%	0.0%	0.0%	0.0%	0.0%	0.0%	0.0%	0.0%	0.7%	0.0%
	k__Bacteria;p__Proteobacteria;c__Gammaproteobacteria;o__Pseudomonadales;f__Pseudomonadaceae	0	0.0%	0.0%	0.1%	0.0%	0.0%	0.0%	0.0%	0.0%	0.0%	0.0%	0.0%	0.0%	0.0%
	k__Bacteria;p__Proteobacteria;c__Gammaproteobacteria;o__Thiotrichales;f__Thiotrichaceae	0	0.0%	0.0%	0.0%	0.0%	0.0%	0.0%	0.0%	0.0%	0.2%	0.0%	0.0%	0.0%	0.1%
	k__Bacteria;p__Proteobacteria;c__Gammaproteobacteria;o__Xanthomonadales;f__Sinobacteraceae	0	0.2%	0.0%	0.0%	0.1%	0.0%	0.1%	1.3%	0.0%	1.2%	0.0%	0.0%	0.0%	0.0%
	k__Bacteria;p__Proteobacteria;c__Gammaproteobacteria;o__Xanthomonadales;f__Xanthomonadaceae	0	0.0%	0.0%	0.0%	0.0%	0.0%	0.0%	0.0%	0.0%	0.0%	0.0%	0.0%	0.4%	0.0%
	k__Bacteria;p__Spirochaetes;c__Spirochaetes;o__Spirochaetales;f__Spirochaetaceae	0	0.3%	0.0%	0.0%	0.0%	4.0%	0.0%	0.0%	0.0%	0.0%	0.0%	0.0%	0.0%	0.0%
	k__Bacteria;p__WPS-2;c__;o__;f__	0	0.2%	0.0%	0.7%	0.0%	0.0%	0.0%	0.0%	0.0%	0.0%	0.3%	0.0%	0.8%	0.1%

Taxonomy Summary. Current Level:

View Figure (.pdf) View Legend (.pdf)

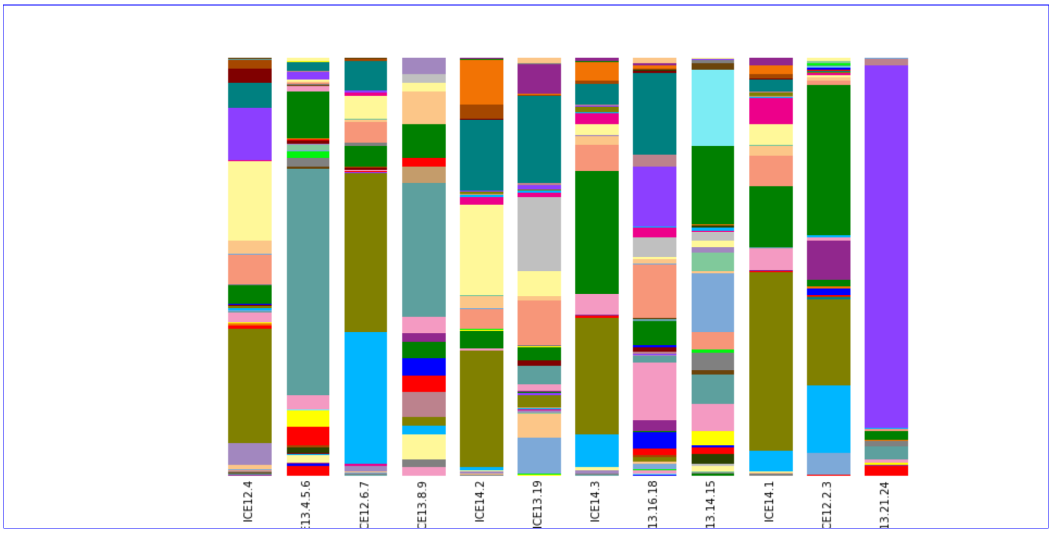

View Table (.txt)

Legend	Taxonomy	Total count	%	ICE12.4 %	ICE13.4.5.6 %	ICE12.6.7 %	ICE13.8.9 %	ICE14.2 %	ICE13.19 %	ICE14.3 %	ICE13.16.18 %	ICE13.14.15 %	ICE14.1 %	ICE12.2.3 %	ICE13.21.24 %
	k__Bacteria;p__Acidobacteria;c__Acidobacteriia;o__Acidobacteriales;f__Acidobacteriaceae;g__	0	0.3%	0.0%	2.3%	0.1%	0.0%	0.0%	0.0%	0.0%	0.0%	0.0%	0.0%	0.3%	0.4%
	k__Bacteria;p__Acidobacteria;c__Acidobacteriia;o__Acidobacteriales;f__Acidobacteriaceae;g__Granulicella	0	0.1%	0.0%	0.5%	0.0%	0.0%	0.0%	0.0%	0.0%	0.2%	0.0%	0.0%	0.0%	0.1%
	k__Bacteria;p__Acidobacteria;c__Acidobacteriia;o__Acidobacteriales;f__Acidobacteriaceae;g__Terriglobus	0	0.0%	0.0%	0.1%	0.0%	0.0%	0.0%	0.0%	0.0%	0.0%	0.0%	0.0%	0.0%	0.0%
	k__Bacteria;p__Acidobacteria;c__Solibacteres;o__Solibacterales;f__;g__	0	0.0%	0.0%	0.0%	0.0%	0.0%	0.0%	0.0%	0.0%	0.0%	0.5%	0.0%	0.0%	0.0%
	k__Bacteria;p__Actinobacteria;c__Actinobacteria;o__Actinomycetales;f__;g__	0	0.0%	0.2%	0.2%	0.0%	0.0%	0.0%	0.1%	0.0%	0.0%	0.0%	0.0%	0.0%	0.0%
	k__Bacteria;p__Actinobacteria;c__Actinobacteria;o__Actinomycetales;f__Frankiaceae;g__	0	0.0%	0.0%	0.0%	0.0%	0.0%	0.0%	0.1%	0.0%	0.0%	0.0%	0.0%	0.0%	0.0%
	k__Bacteria;p__Actinobacteria;c__Actinobacteria;o__Actinomycetales;f__Geodermatophilaceae;g__	0	0.0%	0.0%	0.0%	0.0%	0.0%	0.0%	0.0%	0.0%	0.3%	0.0%	0.0%	0.0%	0.0%
	k__Bacteria;p__Actinobacteria;c__Actinobacteria;o__Actinomycetales;f__Kineosporiaceae;g__	0	0.2%	0.0%	0.0%	0.0%	2.0%	0.0%	0.0%	0.0%	0.9%	0.0%	0.0%	0.0%	0.0%
	k__Bacteria;p__Actinobacteria;c__Actinobacteria;o__Actinomycetales;f__Microbacteriaceae;g__	0	0.1%	0.2%	0.0%	0.1%	0.0%	0.1%	0.1%	0.3%	0.0%	0.0%	0.1%	0.0%	0.0%
	k__Bacteria;p__Actinobacteria;c__Actinobacteria;o__Actinomycetales;f__Microbacteriaceae;g__Mycetocola	0	0.0%	0.5%	0.0%	0.1%	0.0%	0.0%	0.0%	0.0%	0.0%	0.0%	0.0%	0.0%	0.0%

	k__Bacteria;p__Actinobacteria;c__Actinobacteria;o__Actinomycetales;f__Microbacteriaceae;g__Salinibacterium	0	0.3%	0.2%	0.0%	0.1%	2.0%	0.1%	0.1%	0.1%	0.0%	0.3%	0.4%	0.1%	0.0%
	k__Bacteria;p__Actinobacteria;c__Actinobacteria;o__Actinomycetales;f__Nocardiaceae;g__	0	0.0%	0.0%	0.0%	0.0%	0.0%	0.0%	0.1%	0.0%	0.2%	0.0%	0.0%	0.0%	0.0%
	k__Bacteria;p__Actinobacteria;c__Actinobacteria;o__Actinomycetales;f__Pseudonocardiaceae;g__	0	0.0%	0.2%	0.0%	0.0%	0.0%	0.0%	0.1%	0.0%	0.0%	0.0%	0.0%	0.0%	0.0%
	k__Bacteria;p__Actinobacteria;c__Actinobacteria;o__Actinomycetales;f__Pseudonocardiaceae;g__Pseudonocardia	0	1.3%	0.2%	0.1%	0.2%	0.0%	0.0%	8.7%	0.0%	1.2%	0.0%	0.1%	5.1%	0.0%
	k__Bacteria;p__Actinobacteria;c__Actinobacteria;o__Actinomycetales;f__Sporichthyaceae;g__	0	0.7%	0.9%	0.0%	0.4%	0.0%	0.4%	5.7%	0.2%	0.5%	0.0%	0.3%	0.0%	0.0%
	k__Bacteria;p__Actinobacteria;c__Thermoleophilia;o__Gaiellales;f__;g__	0	0.0%	0.2%	0.0%	0.0%	0.0%	0.0%	0.1%	0.0%	0.0%	0.3%	0.0%	0.0%	0.0%
	k__Bacteria;p__Bacteroidetes;c__Cytophagia;o__Cytophagales;f__Cytophagaceae;g__Flectobacillus	0	0.7%	5.2%	0.0%	1.4%	0.0%	0.5%	0.7%	0.7%	0.0%	0.0%	0.1%	0.0%	0.0%
	k__Bacteria;p__Bacteroidetes;c__Cytophagia;o__Cytophagales;f__Cytophagaceae;g__Hymenobacter	0	0.9%	0.0%	1.8%	0.0%	6.0%	0.2%	0.0%	0.9%	0.0%	1.4%	0.1%	0.1%	0.1%
	k__Bacteria;p__Bacteroidetes;c__Cytophagia;o__Cytophagales;f__Cytophagaceae;g__Spirosoma	0	0.0%	0.0%	0.0%	0.0%	0.0%	0.0%	0.0%	0.0%	0.0%	0.5%	0.0%	0.0%	0.0%
	k__Bacteria;p__Bacteroidetes;c__Sphingobacteriia;o__Sphingobacteriales;f__;g__	0	0.0%	0.0%	0.0%	0.4%	0.0%	0.0%	0.2%	0.0%	0.0%	0.0%	0.0%	0.0%	0.0%
	k__Bacteria;p__Bacteroidetes;c__Sphingobacteriia;o__Sphingobacteriales;f__Sphingobacteriaceae;g__	1	5.3%	0.0%	0.2%	31.6%	2.0%	0.8%	0.2%	7.6%	0.2%	0.0%	5.0%	16.0%	0.0%
	k__Bacteria;p__Bacteroidetes;c__Sphingobacteriia;o__Sphingobacteriales;f__Sphingobacteriaceae;g__Pedobacter	0	0.0%	0.0%	0.0%	0.0%	0.0%	0.0%	0.3%	0.0%	0.0%	0.0%	0.0%	0.1%	0.0%
	k__Bacteria;p__Bacteroidetes;c__[Saprospirae];o__[Saprospirales];f__Chitinophagaceae;g__	2	15.9%	27.3%	0.1%	38.1%	2.0%	27.8%	2.9%	28.0%	1.0%	0.0%	42.7%	20.6%	0.0%
	k__Bacteria;p__Bacteroidetes;c__[Saprospirae];o__[Saprospirales];f__Chitinophagaceae;g__Sediminibacterium	0	0.1%	0.0%	0.0%	0.1%	0.0%	0.0%	0.7%	0.0%	0.0%	0.0%	0.0%	0.0%	0.0%
	k__Bacteria;p__Chlorobi;c__Ignavibacteria;o__Ignavibacteriales;f__Ignavibacteriaceae;g__	0	0.5%	0.0%	0.0%	0.0%	6.0%	0.0%	0.0%	0.0%	0.0%	0.0%	0.0%	0.0%	0.0%
	k__Bacteria;p__Chlorobi;c__SJA-28;o__;f__;g__	0	0.0%	0.0%	0.0%	0.0%	0.0%	0.0%	0.0%	0.0%	0.0%	0.0%	0.0%	0.4%	0.0%
	k__Bacteria;p__Chloroflexi;c__Ktedonobacteria;o__Thermogemmatisporales;f__Thermogemmatisporaceae;g__	0	0.0%	0.0%	0.2%	0.0%	0.0%	0.0%	0.0%	0.0%	0.0%	0.0%	0.0%	0.0%	0.0%
	k__Bacteria;p__Cyanobacteria;c__;o__;f__;g__	0	0.3%	0.0%	1.4%	0.0%	0.0%	0.0%	0.0%	0.0%	0.0%	2.2%	0.0%	0.0%	0.0%
	k__Bacteria;p__Cyanobacteria;c__Chloroplast;o__;f__;g__	0	0.1%	0.0%	0.4%	0.0%	0.0%	0.1%	0.0%	0.0%	0.5%	0.0%	0.1%	0.4%	0.1%
	k__Bacteria;p__Cyanobacteria;c__Chloroplast;o__Chlorophyta;f__;g__	0	1.2%	0.7%	4.4%	0.2%	4.0%	0.1%	0.0%	0.5%	1.5%	1.6%	0.1%	0.1%	1.6%
	k__Bacteria;p__Cyanobacteria;c__Chloroplast;o__Chlorophyta;f__Chlamydomonadaceae;g__	0	0.9%	0.2%	0.1%	0.0%	4.0%	0.0%	0.0%	0.0%	3.8%	0.5%	0.0%	1.6%	0.2%
	k__Bacteria;p__Cyanobacteria;c__Chloroplast;o__Chlorophyta;f__Chlamydomonadaceae;g__Acutodesmus	0	0.1%	0.5%	0.0%	0.0%	0.0%	0.0%	0.0%	0.0%	0.0%	0.0%	0.0%	0.4%	0.0%
	k__Bacteria;p__Cyanobacteria;c__Chloroplast;o__Chlorophyta;f__Chlamydomonadaceae;g__Chlamydomonas	0	0.5%	0.0%	0.1%	0.0%	4.0%	0.0%	0.2%	0.0%	0.3%	0.0%	0.1%	1.7%	0.0%
	k__Bacteria;p__Cyanobacteria;c__Chloroplast;o__Chlorophyta;f__Chlamydomonadaceae;g__Oophila	0	1.2%	0.0%	0.0%	0.1%	2.0%	0.0%	0.3%	0.3%	2.6%	0.0%	0.3%	9.4%	0.1%
	k__Bacteria;p__Cyanobacteria;c__Chloroplast;o__Chlorophyta;f__Trebouxiophyceae;g__	0	0.7%	0.2%	3.9%	0.0%	0.0%	0.0%	0.0%	0.0%	0.0%	3.3%	0.0%	0.0%	0.6%
	k__Bacteria;p__Cyanobacteria;c__Chloroplast;o__Chlorophyta;f__Trebouxiophyceae;g__Coccomyxa	0	0.0%	0.0%	0.1%	0.0%	0.0%	0.0%	0.0%	0.0%	0.0%	0.0%	0.0%	0.0%	0.0%
	k__Bacteria;p__Cyanobacteria;c__Chloroplast;o__Stramenopiles;f__;g__	0	3.7%	2.5%	3.4%	0.2%	4.0%	0.5%	1.7%	4.9%	13.9%	6.5%	5.2%	0.7%	0.9%
	k__Bacteria;p__Cyanobacteria;c__Chloroplast;o__Streptophyta;f__;g__	1	8.5%	0.2%	54.2%	0.0%	32.0%	0.1%	4.3%	0.0%	1.4%	7.0%	0.2%	0.1%	2.9%
	k__Bacteria;p__Cyanobacteria;c__Nostocophycideae;o__Nostocales;f__Nostocaceae;g__	0	0.1%	0.0%	0.5%	0.0%	0.0%	0.0%	0.0%	0.0%	0.0%	1.1%	0.0%	0.0%	0.0%
	k__Bacteria;p__Cyanobacteria;c__Nostocophycideae;o__Nostocales;f__Nostocaceae;g__Nostoc	0	0.7%	0.0%	2.2%	0.0%	0.0%	0.0%	0.0%	0.0%	0.0%	4.3%	0.0%	0.0%	1.4%
	k__Bacteria;p__Cyanobacteria;c__Nostocophycideae;o__Nostocales;f__Nostocaceae;g__Stigonema	0	0.2%	0.0%	1.5%	0.0%	0.0%	0.0%	0.0%	0.0%	0.0%	0.5%	0.0%	0.0%	0.0%
	k__Bacteria;p__Cyanobacteria;c__Nostocophycideae;o__Nostocales;f__Nostocaceae;g__Tolypothrix	0	0.4%	0.0%	0.0%	0.0%	0.0%	0.0%	0.0%	0.0%	0.0%	4.3%	0.0%	0.0%	0.0%
	k__Bacteria;p__Cyanobacteria;c__Nostocophycideae;o__Nostocales;f__Nostocaceae;g__Toxopsis	0	1.2%	0.2%	0.3%	0.0%	0.0%	0.0%	0.0%	0.0%	0.0%	14.1%	0.0%	0.0%	0.0%
	k__Bacteria;p__Cyanobacteria;c__Nostocophycideae;o__Stigonematales;f__Rivulariaceae;g__Calothrix	0	0.0%	0.0%	0.0%	0.0%	0.0%	0.0%	0.0%	0.0%	0.0%	0.5%	0.0%	0.0%	0.0%
	k__Bacteria;p__Cyanobacteria;c__Oscillatoriophycideae;o__Chroococcales;f__Xenococcaceae;g__	0	0.5%	0.0%	1.4%	0.0%	0.0%	0.0%	0.0%	0.0%	0.2%	4.3%	0.0%	0.0%	0.0%
	k__Bacteria;p__Cyanobacteria;c__Oscillatoriophycideae;o__Chroococcales;f__Xenococcaceae;g__Chroococcidiopsis	0	0.1%	0.0%	0.1%	0.0%	0.0%	0.0%	0.0%	0.0%	0.2%	1.4%	0.0%	0.0%	0.0%
	k__Bacteria;p__Cyanobacteria;c__Oscillatoriophycideae;o__Oscillatoriales;f__Phormidiaceae;g__Phormidium	0	0.1%	0.0%	0.0%	0.0%	0.0%	0.0%	0.0%	0.0%	0.0%	1.6%	0.0%	0.0%	0.0%
	k__Bacteria;p__Cyanobacteria;c__Synechococcophycideae;o__Pseudanabaenales;f__Pseudanabaenaceae;g__Leptolyngbya	0	0.2%	0.0%	0.0%	0.0%	0.0%	0.0%	0.0%	0.0%	0.0%	1.9%	0.0%	0.0%	0.0%
	k__Bacteria;p__Cyanobacteria;c__Synechococcophycideae;o__Synechococcales;f__Acaryochloridaceae;g__Acaryochloris	0	0.0%	0.0%	0.1%	0.0%	0.0%	0.0%	0.0%	0.0%	0.0%	0.3%	0.0%	0.0%	0.0%
	k__Bacteria;p__Cyanobacteria;c__Synechococcophycideae;o__Synechococcales;f__Chamaesiphonaceae;g__	0	0.1%	0.5%	0.1%	0.0%	0.0%	0.0%	0.0%	0.0%	0.0%	0.8%	0.0%	0.4%	0.0%
	k__Bacteria;p__Firmicutes;c__Bacilli;o__Bacillales;f__Alicyclobacillaceae;g__Alicyclobacillus	0	0.3%	0.0%	0.0%	0.0%	4.0%	0.0%	0.1%	0.0%	0.0%	0.0%	0.0%	0.0%	0.0%
	k__Bacteria;p__Gemmatimonadetes;c__Gemmatimonadetes;o__Gemmatimonadales;f__Ellin5301;g__	0	0.0%	0.5%	0.0%	0.0%	0.0%	0.0%	0.1%	0.0%	0.0%	0.0%	0.0%	0.1%	0.0%
	k__Bacteria;p__Proteobacteria;c__Alphaproteobacteria;o__;f__;g__	0	0.0%	0.0%	0.0%	0.0%	0.0%	0.0%	0.0%	0.0%	0.3%	0.0%	0.0%	0.0%	0.0%
	k__Bacteria;p__Proteobacteria;c__Alphaproteobacteria;o__Caulobacterales;f__Caulobacteraceae;g__	0	0.0%	0.0%	0.0%	0.0%	0.0%	0.0%	0.0%	0.0%	0.3%	0.0%	0.0%	0.0%	0.0%
	k__Bacteria;p__Proteobacteria;c__Alphaproteobacteria;o__Rhizobiales;f__Beijerinckiaceae;g__Beijerinckia	0	0.0%	0.0%	0.1%	0.0%	0.0%	0.0%	0.0%	0.0%	0.0%	0.0%	0.0%	0.0%	0.0%
	k__Bacteria;p__Proteobacteria;c__Alphaproteobacteria;o__Rhizobiales;f__Bradyrhizobiaceae;g__Bradyrhizobium	0	0.3%	0.5%	0.7%	0.6%	0.0%	0.0%	1.1%	0.0%	1.2%	0.0%	0.1%	0.0%	0.0%
	k__Bacteria;p__Proteobacteria;c__Alphaproteobacteria;o__Rhizobiales;f__Hyphomicrobiaceae;g__Devosia	0	0.0%	0.0%	0.0%	0.0%	0.0%	0.0%	0.0%	0.0%	0.0%	0.5%	0.0%	0.0%	0.0%
	k__Bacteria;p__Proteobacteria;c__Alphaproteobacteria;o__Rhizobiales;f__Hyphomicrobiaceae;g__Rhodoplanes	0	0.0%	0.0%	0.0%	0.2%	0.0%	0.0%	0.1%	0.0%	0.0%	0.0%	0.0%	0.1%	0.0%
	k__Bacteria;p__Proteobacteria;c__Alphaproteobacteria;o__Rhizobiales;f__Methylobacteriaceae;g__	0	0.2%	0.0%	0.2%	0.0%	2.0%	0.0%	0.0%	0.0%	0.0%	0.0%	0.0%	0.0%	0.0%
	k__Bacteria;p__Proteobacteria;c__Alphaproteobacteria;o__Rhizobiales;f__Methylobacteriaceae;g__Methylobacterium	0	0.0%	0.2%	0.0%	0.0%	0.0%	0.0%	0.0%	0.0%	0.3%	0.0%	0.0%	0.0%	0.0%
	k__Bacteria;p__Proteobacteria;c__Alphaproteobacteria;o__Rhizobiales;f__Methylocystaceae;g__	0	0.1%	0.0%	0.3%	0.0%	0.0%	0.0%	0.0%	0.0%	0.0%	0.3%	0.0%	0.0%	0.4%
	k__Bacteria;p__Proteobacteria;c__Alphaproteobacteria;o__Rhodospirillales;f__Acetobacteraceae;g__	1	11.8%	4.3%	11.2%	4.8%	8.0%	4.2%	3.3%	29.3%	5.9%	18.7%	14.5%	35.7%	1.9%
	k__Bacteria;p__Proteobacteria;c__Alphaproteobacteria;o__Rhodospirillales;f__Rhodospirillaceae;g__	0	0.0%	0.0%	0.0%	0.0%	0.0%	0.0%	0.0%	0.0%	0.0%	0.0%	0.1%	0.0%	0.0%
	k__Bacteria;p__Proteobacteria;c__Alphaproteobacteria;o__Rickettsiales;f__;g__	0	0.0%	0.0%	0.0%	0.0%	0.0%	0.1%	0.1%	0.0%	0.0%	0.0%	0.0%	0.0%	0.0%
	k__Bacteria;p__Proteobacteria;c__Alphaproteobacteria;o__Rickettsiales;f__mitochondria;g__Anomodon	0	1.5%	0.0%	0.0%	0.0%	0.0%	0.0%	0.0%	0.0%	0.0%	18.2%	0.0%	0.0%	0.0%
	k__Bacteria;p__Proteobacteria;c__Alphaproteobacteria;o__Rickettsiales;f__mitochondria;g__Syntrichia	0	0.1%	0.0%	1.3%	0.0%	0.0%	0.0%	0.0%	0.0%	0.0%	0.0%	0.0%	0.0%	0.0%
	k__Bacteria;p__Proteobacteria;c__Alphaproteobacteria;o__Sphingomonadales;f__;g__	0	0.0%	0.0%	0.0%	0.0%	0.0%	0.0%	0.0%	0.0%	0.5%	0.0%	0.0%	0.0%	0.0%
	k__Bacteria;p__Proteobacteria;c__Alphaproteobacteria;o__Sphingomonadales;f__Sphingomonadaceae;g__	0	0.2%	0.0%	0.2%	0.0%	0.0%	0.2%	0.0%	0.0%	0.2%	1.6%	0.0%	0.0%	0.1%
	k__Bacteria;p__Proteobacteria;c__Alphaproteobacteria;o__Sphingomonadales;f__Sphingomonadaceae;g__Sphingomonas	0	0.2%	0.2%	0.2%	0.9%	0.0%	0.1%	0.3%	0.0%	0.0%	0.8%	0.0%	0.2%	0.0%
	k__Bacteria;p__Proteobacteria;c__Betaproteobacteria;o__;f__;g__	0	0.0%	0.0%	0.0%	0.0%	0.0%	0.1%	0.0%	0.0%	0.0%	0.0%	0.0%	0.0%	0.0%
	k__Bacteria;p__Proteobacteria;c__Betaproteobacteria;o__Burkholderiales;f__;g__	1	4.6%	7.2%	0.4%	4.8%	0.0%	4.7%	10.7%	6.3%	12.9%	0.0%	7.1%	1.0%	0.4%
	k__Bacteria;p__Proteobacteria;c__Betaproteobacteria;o__Burkholderiales;f__Burkholderiaceae;g__	0	0.1%	0.2%	0.0%	0.1%	0.0%	0.2%	0.0%	0.0%	0.2%	0.0%	0.0%	0.0%	0.0%
	k__Bacteria;p__Proteobacteria;c__Betaproteobacteria;o__Burkholderiales;f__Comamonadaceae;g__	0	1.8%	3.2%	0.3%	0.5%	8.0%	3.0%	1.0%	2.1%	1.0%	0.0%	2.5%	0.7%	0.0%
	k__Bacteria;p__Proteobacteria;c__Betaproteobacteria;o__Burkholderiales;f__Comamonadaceae;g__Leptothrix	0	0.1%	0.0%	0.0%	0.1%	0.0%	0.3%	0.1%	0.1%	0.0%	0.0%	0.1%	0.0%	0.0%
	k__Bacteria;p__Proteobacteria;c__Betaproteobacteria;o__Burkholderiales;f__Comamonadaceae;g__Limnohabitans	0	0.0%	0.0%	0.0%	0.0%	0.0%	0.1%	0.1%	0.1%	0.0%	0.0%	0.0%	0.0%	0.0%
	k__Bacteria;p__Proteobacteria;c__Betaproteobacteria;o__Burkholderiales;f__Comamonadaceae;g__Methylibium	1	5.3%	18.9%	0.5%	5.6%	2.0%	21.4%	6.0%	2.7%	0.5%	0.0%	4.9%	0.7%	0.0%
	k__Bacteria;p__Proteobacteria;c__Betaproteobacteria;o__Burkholderiales;f__Comamonadaceae;g__Paucibacter	0	2.0%	0.0%	0.0%	0.0%	2.0%	0.0%	17.6%	0.0%	4.8%	0.0%	0.0%	0.0%	0.0%
	k__Bacteria;p__Proteobacteria;c__Betaproteobacteria;o__Burkholderiales;f__Comamonadaceae;g__Polaromonas	0	1.3%	0.2%	0.0%	0.7%	0.0%	1.9%	1.0%	2.5%	2.2%	0.0%	6.4%	0.4%	0.0%
	k__Bacteria;p__Proteobacteria;c__Betaproteobacteria;o__Burkholderiales;f__Comamonadaceae;g__Rhodoferax	0	0.2%	0.0%	0.0%	0.1%	0.0%	0.6%	0.4%	0.3%	0.2%	0.0%	0.2%	0.0%	0.1%
	k__Bacteria;p__Proteobacteria;c__Betaproteobacteria;o__Burkholderiales;f__Comamonadaceae;g__Rubrivivax	0	0.0%	0.0%	0.0%	0.0%	0.0%	0.1%	0.0%	0.0%	0.0%	0.0%	0.2%	0.0%	0.0%
	k__Bacteria;p__Proteobacteria;c__Betaproteobacteria;o__Burkholderiales;f__Comamonadaceae;g__Variovorax	0	0.3%	0.0%	0.0%	0.0%	0.0%	0.6%	0.3%	1.4%	0.0%	0.0%	0.9%	0.3%	0.0%
	k__Bacteria;p__Proteobacteria;c__Betaproteobacteria;o__Burkholderiales;f__Oxalobacteraceae;g__	1	9.8%	12.6%	1.9%	0.5%	0.0%	0.3%	1.2%	0.2%	14.3%	0.3%	0.0%	0.0%	86.8%
	k__Bacteria;p__Proteobacteria;c__Betaproteobacteria;o__Burkholderiales;f__Oxalobacteraceae;g__Collimonas	0	0.5%	0.0%	0.2%	0.0%	0.0%	0.1%	0.4%	0.1%	2.9%	0.0%	0.1%	0.0%	1.7%
	k__Bacteria;p__Proteobacteria;c__Betaproteobacteria;o__Burkholderiales;f__Oxalobacteraceae;g__Herminiimonas	1	6.7%	5.9%	2.1%	7.1%	0.0%	16.9%	20.9%	4.9%	19.6%	0.0%	3.0%	0.3%	0.0%
	k__Bacteria;p__Proteobacteria;c__Betaproteobacteria;o__Burkholderiales;f__Oxalobacteraceae;g__Janthinobacterium	0	0.4%	3.6%	0.0%	0.0%	0.0%	0.3%	0.1%	0.0%	0.5%	0.0%	0.1%	0.0%	0.0%
	k__Bacteria;p__Proteobacteria;c__Betaproteobacteria;o__Ellin6067;f__;g__	0	0.0%	0.0%	0.0%	0.0%	0.0%	0.0%	0.0%	0.0%	0.3%	0.0%	0.0%	0.0%	0.0%
	k__Bacteria;p__Proteobacteria;c__Betaproteobacteria;o__Gallionellales;f__Gallionellaceae;g__Gallionella	0	0.7%	2.0%	0.0%	0.4%	0.0%	3.4%	0.2%	0.9%	0.2%	0.0%	1.0%	0.0%	0.0%
	k__Bacteria;p__Proteobacteria;c__Betaproteobacteria;o__IS-44;f__;g__	0	0.0%	0.0%	0.0%	0.0%	0.0%	0.0%	0.0%	0.0%	0.0%	0.0%	0.0%	0.2%	0.0%
	k__Bacteria;p__Proteobacteria;c__Betaproteobacteria;o__Methylophilales;f__;g__	0	0.0%	0.0%	0.0%	0.0%	0.0%	0.0%	0.0%	0.0%	0.0%	0.0%	0.0%	0.4%	0.0%
	k__Bacteria;p__Proteobacteria;c__Betaproteobacteria;o__Methylophilales;f__Methylophilaceae;g__	0	1.5%	0.0%	0.0%	0.0%	0.0%	10.5%	0.1%	4.5%	0.7%	0.0%	2.2%	0.0%	0.0%
	k__Bacteria;p__Proteobacteria;c__Betaproteobacteria;o__Methylophilales;f__Methylophilaceae;g__Methylotenera	0	0.0%	0.0%	0.0%	0.0%	0.0%	0.3%	0.0%	0.2%	0.0%	0.0%	0.1%	0.0%	0.0%
	k__Bacteria;p__Proteobacteria;c__Betaproteobacteria;o__Rhodocyclales;f__Rhodocyclaceae;g__	0	0.9%	0.0%	0.1%	0.1%	0.0%	0.3%	7.2%	0.6%	0.5%	0.0%	1.8%	0.1%	0.0%
	k__Bacteria;p__Proteobacteria;c__Betaproteobacteria;o__SC-I-84;f__;g__	0	0.0%	0.0%	0.1%	0.0%	0.0%	0.0%	0.0%	0.0%	0.0%	0.0%	0.0%	0.0%	0.0%
	k__Bacteria;p__Proteobacteria;c__Deltaproteobacteria;o__MBNT15;f__;g__	0	0.0%	0.0%	0.0%	0.0%	0.0%	0.0%	0.0%	0.0%	0.0%	0.0%	0.0%	0.1%	0.0%
	k__Bacteria;p__Proteobacteria;c__Deltaproteobacteria;o__Myxococcales;f__Haliangiaceae;g__	0	0.0%	0.0%	0.0%	0.0%	0.0%	0.0%	0.0%	0.0%	0.0%	0.0%	0.0%	0.1%	0.0%
	k__Bacteria;p__Proteobacteria;c__Deltaproteobacteria;o__Myxococcales;f__Polyangiaceae;g__Aetherobacter	0	0.0%	0.2%	0.1%	0.0%	0.0%	0.0%	0.0%	0.0%	0.0%	0.0%	0.0%	0.1%	0.0%
	k__Bacteria;p__Proteobacteria;c__Deltaproteobacteria;o__Spirobacillales;f__;g__	0	0.0%	0.2%	0.0%	0.1%	0.0%	0.0%	0.0%	0.0%	0.0%	0.0%	0.0%	0.0%	0.0%
	k__Bacteria;p__Proteobacteria;c__Gammaproteobacteria;o__;f__;g__	0	0.0%	0.0%	0.0%	0.0%	0.0%	0.0%	0.2%	0.0%	0.0%	0.0%	0.0%	0.0%	0.0%
	k__Bacteria;p__Proteobacteria;c__Gammaproteobacteria;o__Legionellales;f__Coxiellaceae;g__	0	0.1%	0.0%	0.0%	0.0%	0.0%	0.0%	0.0%	0.0%	0.0%	0.0%	0.0%	0.7%	0.0%
	k__Bacteria;p__Proteobacteria;c__Gammaproteobacteria;o__Pseudomonadales;f__Pseudomonadaceae;g__Pseudomonas	0	0.0%	0.0%	0.1%	0.0%	0.0%	0.0%	0.0%	0.0%	0.0%	0.0%	0.0%	0.0%	0.0%
	k__Bacteria;p__Proteobacteria;c__Gammaproteobacteria;o__Thiotrichales;f__Thiotrichaceae;g__Thiothrix	0	0.0%	0.0%	0.0%	0.0%	0.0%	0.0%	0.0%	0.0%	0.2%	0.0%	0.0%	0.0%	0.1%
	k__Bacteria;p__Proteobacteria;c__Gammaproteobacteria;o__Xanthomonadales;f__Sinobacteraceae;g__	0	0.2%	0.0%	0.0%	0.1%	0.0%	0.1%	1.3%	0.0%	1.2%	0.0%	0.0%	0.0%	0.0%
	k__Bacteria;p__Proteobacteria;c__Gammaproteobacteria;o__Xanthomonadales;f__Xanthomonadaceae;g__	0	0.0%	0.0%	0.0%	0.0%	0.0%	0.0%	0.0%	0.0%	0.0%	0.0%	0.0%	0.4%	0.0%
	k__Bacteria;p__Spirochaetes;c__Spirochaetes;o__Spirochaetales;f__Spirochaetaceae;g__Treponema	0	0.3%	0.0%	0.0%	0.0%	4.0%	0.0%	0.0%	0.0%	0.0%	0.0%	0.0%	0.0%	0.0%
	k__Bacteria;p__WPS-2;c__;o__;f__;g__	0	0.2%	0.0%	0.7%	0.0%	0.0%	0.0%	0.0%	0.0%	0.0%	0.3%	0.0%	0.8%	0.1%
